# Electrochemical Monitoring of Heterogeneous Peroxygenase Reactions Unravels LPMO Kinetics

**DOI:** 10.1021/acscatal.3c05194

**Published:** 2024-01-10

**Authors:** Lorenz Schwaiger, Florian Csarman, Hucheng Chang, Ole Golten, Vincent G. H. Eijsink, Roland Ludwig

**Affiliations:** † Department of Food Science and Technology, Institute of Food Technology, University of Natural Resources and Life Sciences, Vienna (BOKU), Muthgasse 18, 1190 Vienna, Austria; ‡ Faculty of Chemistry, Biotechnology and Food Science, Norwegian University of Life Sciences (NMBU), P.O. Box 5003, NO-1432 Ås, Norway

**Keywords:** biocatalysis, electrochemical enzyme assay, heterogeneous substrates, lytic polysaccharide monooxygenase, peroxygenase activity, turnover stability

## Abstract

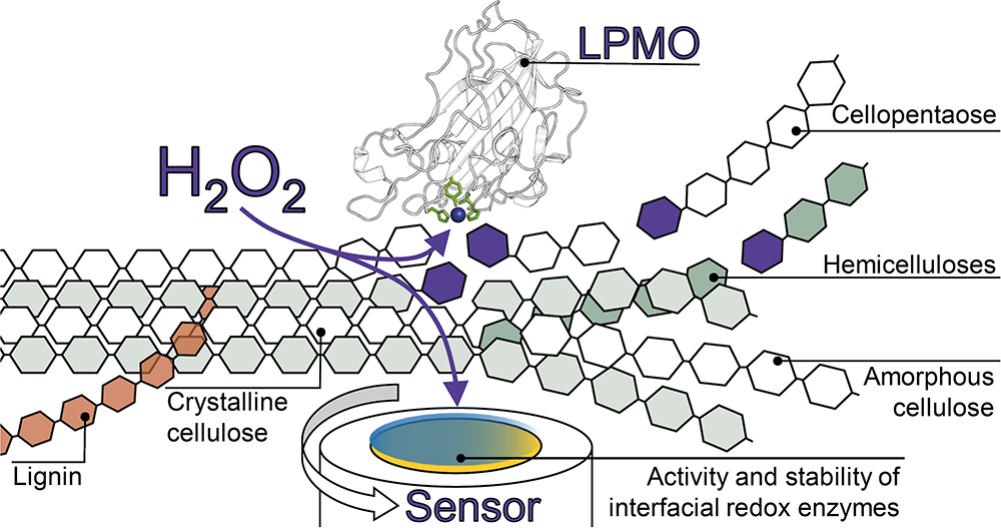

Biological conversion of plant biomass depends on peroxygenases and peroxidases acting on insoluble polysaccharides and lignin. Among these are cellulose- and hemicellulose-degrading lytic polysaccharide monooxygenases (LPMOs), which have revolutionized our concept of biomass degradation. Major obstacles limiting mechanistic and functional understanding of these unique peroxygenases are their complex and insoluble substrates and the hard-to-measure H_2_O_2_ consumption, resulting in the lack of suitable kinetic assays. We report a versatile and robust electrochemical method for real-time monitoring and kinetic characterization of LPMOs and other H_2_O_2_-dependent interfacial enzymes based on a rotating disc electrode for the sensitive and selective quantitation of H_2_O_2_ at biologically relevant concentrations. The H_2_O_2_ sensor works in suspensions of insoluble substrates as well as in homogeneous solutions. Our characterization of multiple LPMOs provides unprecedented insights into the substrate specificity, kinetics, and stability of these enzymes. High turnover and total turnover numbers demonstrate that LPMOs are fast and durable biocatalysts.

## Introduction

Peroxidases and peroxygenases are widespread enzymes that are formed from a variety of protein folds, utilize different prosthetic groups, and catalyze a myriad of reactions in biological and biotechnological processes. The unifying principle of these enzymes is that they use H_2_O_2_ as an oxidant. Many enzyme assays have been developed to study these important enzymes, but a recently discovered class of enzymes that perform the depolymerization of polysaccharides in plant cell walls and other biological structures overpowered available methods, frustrating their detailed kinetic characterization.

**Figure 1:**
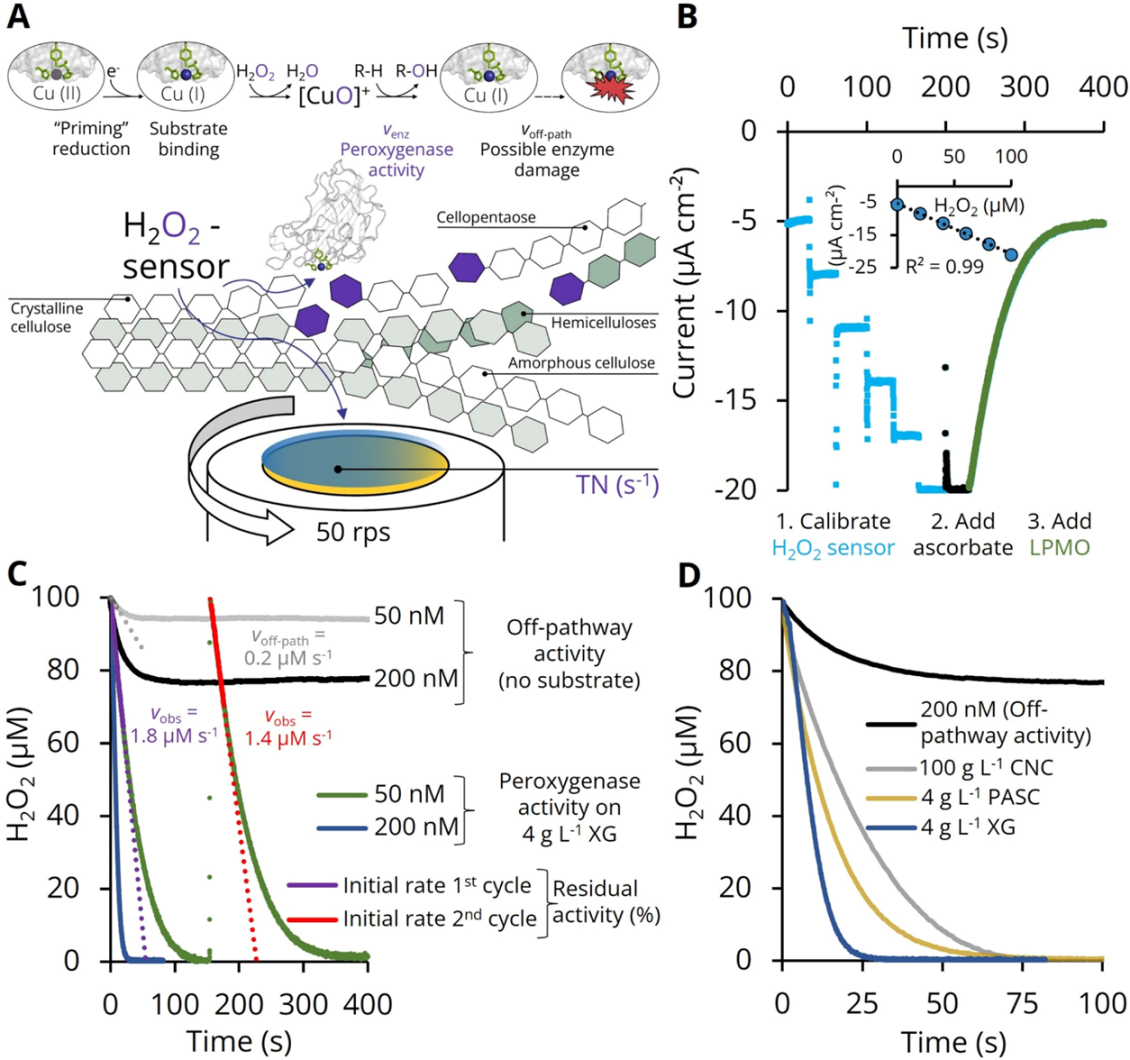
Real-time detection of peroxygenase activity. (A) A Prussian blue-modified rotating disc electrode (RDE) is used to amperometrically measure the H_2_O_2_ consumption associated with LPMO activity on soluble and dispersed substrates. (B) Each measurement consists of sensor calibration by titration with H_2_O_2_ (blue dots) in the presence of the substrate, followed by addition of the reducing agent (black dots) and, finally, addition of the LPMO (green dots). The standard curve (inset) is a linear regression of the H_2_O_2_ calibration data and is used to convert current density (μA cm^–2^) into H_2_O_2_ concentration (μM). (C) H_2_O_2_ time traces for peroxygenase reactions of 50 (green curve) and 200 nM (blue curve) *Nc*AA9C acting on 4 g L^–1^ xyloglucan (XG) or without substrate (off-pathway activity, gray and black curve) in 30 mM sodium acetate buffer pH 6.0 containing 100 mM KCl and 500 μM ascorbate at 30 °C. The initial rate for the enzymatic LPMO activity (*v*_enz_ = 1.6 μM s^–1^) is calculated by subtracting *v*_off-path_ (gray dots) from *v*_obs_ (purple dots) in the 1st reaction cycle. The residual activity after the 1st measurement is determined by adding the same amount of H_2_O_2_ again and calculating *v*_enz_ from the 2nd measurement cycle (red dots). (D) *Nc*AA9C (200 nM) acting on soluble xyloglucan or dispersed phosphoric acid-swollen cellulose (PASC) and crystalline nanocellulose (CNC) as substrates. The off-pathway consumption of H_2_O_2_ by 200 nM *Nc*AA9C in the absence of substrate is shown in black (same data set is shown in panel C). The TNs of these reactions are calculated from triplicate measurements.

Lytic polysaccharide monooxygenases (LPMOs) are abundant enzymes featuring an open, planar substrate binding site^1−3^ in combination with a surface-exposed catalytic copper site^4^ to depolymerize plant,^5^ insect,^6^ and marine^7^ polysaccharides such as cellulose,^8^ hemicelluloses,^5,9,10^ chitin,^3^ starch,^11^ or pectin.^12^ Their function extends beyond biomass degradation, and their role in physiological processes such as plant and microbial virulence is an emerging field of research.^13−15^ LPMOs cleave glycosidic bonds via site-selective C–H bond activation by using an oxygen species as a cosubstrate and electron donors like ascorbate,^3^ gallic acid,^4^ cysteine,^16^ or the auxiliary enzyme cellobiose dehydrogenase^17,18^ as an activator. Initially, it was thought that O_2_ functions as a cosubstrate,^3^ hence the perception of LPMOs as sluggish monooxygenases with low catalytic efficiencies.^19,20^ Later, it was discovered that H_2_O_2_ is the cosubstrate^21^ and that LPMOs are copper peroxygenases with, at least in some cases, catalytic efficiencies similar to heme peroxygenases.^21−25^ The peroxygenase reaction requires the reductive activation of the LPMO’s monocopper site and most likely involves homolysis of H_2_O_2_,^23,26−28^ yielding a caged hydroxyl radical,^21,26,28^ which aids in generating a copper-oxyl species. This reactive intermediate is powerful enough to abstract a hydrogen atom^26−29^ at the C1- or C4-position of the scissile glycosidic bond,^30^ which eventually leads to hydroxylation of the carbon and glycosidic bond cleavage (Figure 1A).

LPMOs are truly unique enzymes, not only because they are the only known copper peroxygenases but also because of their ability to act on insoluble recalcitrant substrates, which is of great industrial importance. The plethora of possible side reactions and autocatalytic enzyme inactivation^31^ have hampered the in-depth functional characterization of these enzymes. LPMO-catalyzed reactions are currently studied by discontinuous analysis of oxidized reaction products using high-performance liquid chromatography (HPLC) and mass spectrometry (MS) methods.^5,8,9,32,33^ This is an adequate approach to identify new substrates;^10−12^ however, the reactions are performed under the so-called monooxygenase conditions, the rates of which are limited by the *in situ* production of H_2_O_2_ from O_2_, resulting from the LPMO’s oxidase activity^34^ and abiotic oxidation of the reductant. Under such conditions, which are standard in the field, catalysis tends to be excessively slow (0.002–0.3 s^–1^),^3,19,21,23,35,36^ meaning that activities may be missed, while apparent rates do not provide any information about the true catalytic potential of the enzyme.

Using currently available methods to assess the much faster peroxygenase reaction rates is extremely difficult.^20,25,37^ A kinetic characterization method based on the reliable detection of the H_2_O_2_ consumption is not available due, in part, to the insoluble nature of key substrates, preventing the use of spectroscopic methods. Furthermore, steady-state concentrations of H_2_O_2_ in stable LPMO peroxygenase reactions are low, i.e., difficult to measure, while oxidative self-inactivation occurs at higher H_2_O_2_ concentrations. These limitations prompted us to develop a fast, sensitive, and robust electrochemical assay based on a new type of H_2_O_2_ sensor that combines the fast response times of a rotating disc electrode with the H_2_O_2_ selectivity that can be obtained by using Prussian blue as an electrocatalyst. The fast mixing by the rotating disc electrode minimizes mass transfer limitations that otherwise are prominent in reactions with interfacial enzymes bound to solid substrates.

We show that the new method can be used to measure the initial rates of H_2_O_2_ consumption of a variety of fungal auxiliary activity family 9 (AA9) LPMOs (Table S1) acting on any soluble or insoluble oligo- and polysaccharide. The method is generally applicable for H_2_O_2_-consuming enzymes, such as peroxygenases or peroxidases, as illustrated by monitoring a reaction of horseradish peroxidase acting on lignin. The new method allowed us to generate unprecedented insight into the peroxygenase reactions catalyzed by several well-studied LPMOs, including quantitative assessment of substrate specificity, pH dependence, and determination of total turnover numbers (TTNs).

## Results

### H_2_O_2_ Sensor Development and Operation

An electrochemical design based on a rotating disc electrode was selected to minimize mass transfer limitations and sensor response time. The sensitivity of the gold rotating disc electrode to H_2_O_2_ was enhanced by electrodepositing a thin layer of Prussian blue as an electrocatalyst (Figure S1). The resulting sensitivity of the working electrode is high enough to apply a low potential of 100 mV vs SHE for H_2_O_2_ detection, thereby preventing the unwanted electrooxidation of ascorbate and the reduction of O_2_ at the electrode.^38,39^ The increased selectivity of the sensor for H_2_O_2_ allows measurements in air-saturated buffers. The working electrode is rotating with an angular velocity of 50 s^–1^, thereby inducing forced convection in the liquid volume (4 mL) of the electrochemical reaction cell ensuring a constant transport of reactants to the electrode surface. Consequently, at high angular velocities, the mass transfer limitation of reactants is minimized, i.e., the steady-state current is controlled by forced convection rather than diffusion, leading to a greatly reduced response time and increased sensitivity. The rotating disc electrode quickly reaches steady-state currents (in less than 2 s) even in highly concentrated substrate solutions or suspensions (Figure S2). Therefore, the measurement of the H_2_O_2_ concentration is much faster than the enzymatic reaction and consumes less than 1% of the available H_2_O_2_ for the detection reaction (H_2_O_2_ + 2H^+^ + 2e^–^ → 2 H_2_O) over the measured period. The enzymatic activity is calculated from the initial rate (*v*_enz_), which is obtained by subtracting the off-pathway activity of LPMO that occurs in the absence of the substrate and leads to self-inactivation (*v*_off-path_) from the observed rate (*v*_obs_). This correction of *v*_obs_ by subtraction of *v*_off-path_ slightly underestimates the rate of the peroxygenase reaction because the off-pathway reaction is reduced in the presence of a substrate (Figure S3G). The calculated *v*_enz_ (μM s^–1^) is converted to enzyme turnover numbers (TN, s^–1^) by division with the enzyme concentration (μM) in the electrochemical cell. Turnover numbers are independent of the enzyme concentration in the measurements. The residual activity after the first measurement is determined by adding the same amount of H_2_O_2_ again and calculating the TN for the second measurement cycle. The validation process showed that the Prussian blue-based sensor is stable, has an excellent sensitivity between pH 4.0 and 8.0 of 220–260 nA μM^–1^ cm^–2^, and a limit of quantification of 4.4–7.5 μM H_2_O_2_ in various buffers and even in the presence of high concentrations (up to 100 g L^–1^) of insoluble carbohydrates (Table S2). As a preliminary experiment to test the developed sensor under extreme conditions, we performed a horseradish peroxidase-catalyzed oxidation of Kraft lignin. Kraft lignin is obtained by alkali treatment of lignin, partially soluble in water and contains several electroactive species. In this turbid solution of 100 g L^–1^ lignin, we were able to measure a TN of 3.0 s^–1^ for the horseradish peroxidase-catalyzed reaction without interferences (Figure S4). The principle of the H_2_O_2_ sensor, its operation, and its calibration are depicted in Figure 1A,B and described in the Experimental Procedures section.

### LPMO Activity

These measurements revealed high turnover numbers for the peroxygenase reaction of *Neurospora crassa* AA9C (*Nc*AA9C) at saturating ascorbate concentrations when acting on the soluble hemicellulose xyloglucan (31.9 s^–1^) or the dispersed celluloses phosphoric acid-swollen cellulose (PASC, 16.9 s^–1^) and crystalline nanocellulose (CNC, 10.5 s^–1^; Figure 1C,D and Table S3). A linear dose–response relationship is obtained with a 200 nM enzyme concentration (*v*_enz_ = 6.4 s^–1^), which is 4-fold higher than with 50 nM enzyme (*v*_enz_ = 1.6 s^–1^). Importantly, the linear dose–response relationship between the LPMO concentration and the TN calculated from the initial rate of H_2_O_2_ depletion allows the determination of the residual activity (%) of LPMO after the first measurement by adding H_2_O_2_ for a second time (Figures 1C, S3, and Table S3). Depending on the reaction conditions, LPMOs inactivate slowly as a result of oxidative damage. Various experiments were performed to determine appropriate (i.e., saturating) concentrations of the reductant and to show that the obtained catalytic rates are independent of the type of reductant (i.e., ascorbic acid or CDH) (Figures S5, S6, Tables S3, and S4). Furthermore, the H_2_O_2_ sensor was validated by using high-performance anion-exchange chromatography/pulsed amperometric detection (HPAEC-PAD) analysis of reaction products generated by *Nc*AA9C acting on cellopentaose (Figures S7 and S8), which showed a stoichiometry of 1:1.2 ± 0.1 (valid between 0.1 and 1 mM cellopentaose). Similarly, analysis of the products generated by *Nc*AA9F acting on PASC (Table S5) showed a stoichiometry of 1:1.1 ± 0.2 (valid up to 10 min). In summary, these experiments showed that the H_2_O_2_ sensor accurately reflects the glycosidic bond cleavage, with an approximate stoichiometry of 1 for glycosidic bonds cleaved and H_2_O_2_ consumed.

### Off-Pathway Reaction

Measurements with six AA9 LPMOs from *N. crassa* (*Nc*AA9C, *Nc*AA9F, and *Nc*AA9M), *Gloeophyllum trabeum* (*Gt*AA9B), Phanerochaete chrysosporium (*Pc*AA9D), and *Hypocrea jecorina* (*Hj*AA9B) acting on various substrates resulted in TNs between 1.2 and 62.5 s^–1^ (Figure S3). This is much higher compared to the rates typically observed in apparent monooxygenase reactions (0.002–0.3 s^–1^)^19,35^ and supports H_2_O_2_ as the kinetically relevant cosubstrate of LPMOs. Figure S3 also shows that in the absence of substrate, but in the presence of ascorbate and H_2_O_2_, the AA9 LPMOs consume H_2_O_2_ in an off-pathway peroxidase-like reaction^40^ faster (2–4 s^–1^) than they would react with O_2_. Depending on the respective LPMO and its substrate, this off-pathway activity is 2–15 times slower than the peroxygenase reaction. The fact that not every futile turnover results in enzyme inactivation is demonstrated by an average consumption of approximately 120 H_2_O_2_ molecules per LPMO molecule prior to enzyme inactivation (Figure S3G), comparable to results reported for *Tr*AA9A and *Nc*AA9C by Kuusk et al.^41^ Importantly, futile H_2_O_2_ turnover leads to increased ascorbate consumption and the probability of the off-pathway reaction is expected to be decreased by the presence of a substrate and would thus be substrate concentration-dependent, as was indeed observed for *Nc*AA9C. A 4 g L^–1^ xyloglucan concentration resulted in a twice higher degree of H_2_O_2_ conversion compared to a concentration of 0.25 g L^–1^ xyloglucan in the presence of a limiting amount of 2 μM ascorbate (Figure S9).

### Substrate Specificity and Enzyme Kinetics

**Figure 2:**
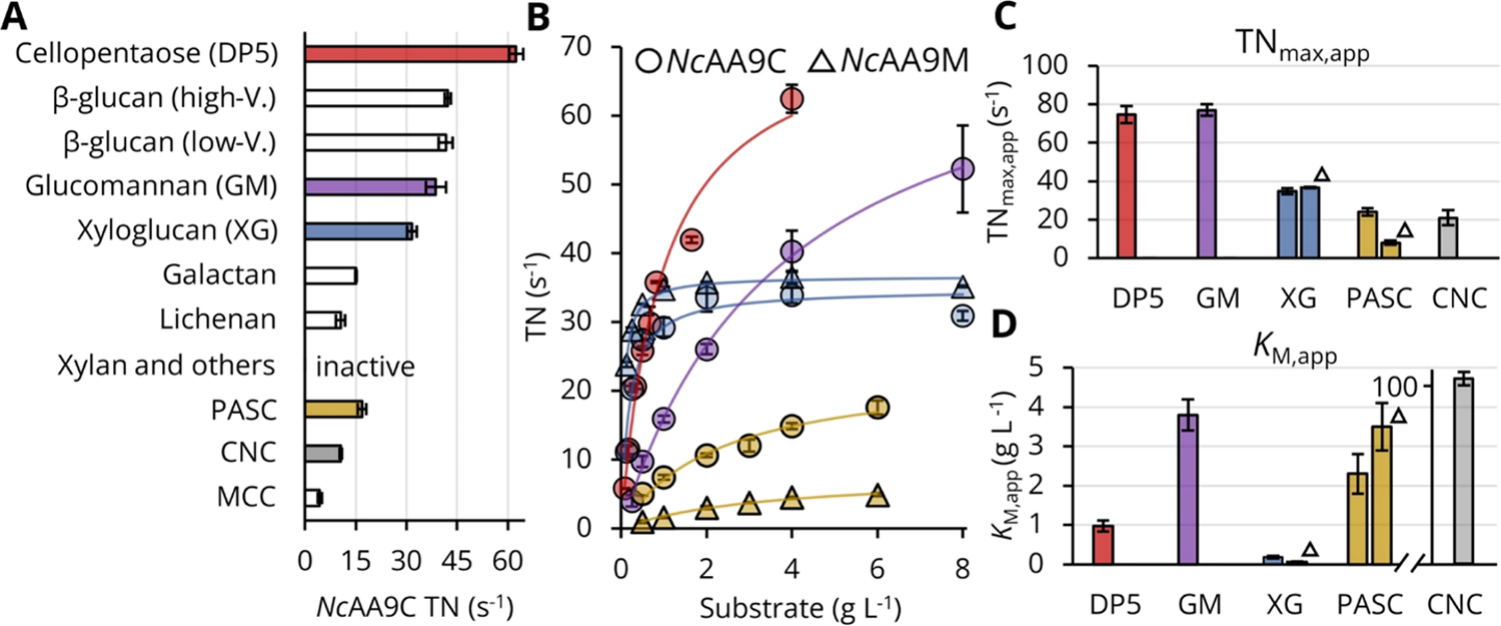
Steady-state kinetic characterization of *Nc*AA9C and *Nc*AA9M acting on soluble and dispersed substrates. (A) TNs for *Nc*AA9C acting on 4 g L^–1^ cellopentaose, hemicelluloses or PASC, or on 100 g L^–1^ CNC or MCC (Figure S10 and Table S6). *Nc*AA9C is inactive on xylan, arabinoxylan, mannan, or curdlan. (B) Substrate concentration-dependent activity of *Nc*AA9C (circles) and *Nc*AA9M (triangles) on selected substrates (shown in the same color code as in panel A; Figures S7 and S11–S14). (C, D) Apparent kinetic constants *K*_M,app_ and TN_max,app_ for *Nc*AA9C and *Nc*AA9M (triangles) calculated from panel B by curve fitting to a hyperbola using least-squares regression (Table S8). Data were measured in triplicate using standard assay conditions given in the Experimental Procedures section. The LPMO concentration in reactions with cellopentaose and hemicelluloses was 50 nM; for celluloses, the LPMO concentration was either 100 nM (*Nc*AA9C acting on PASC) or 200 nM (*Nc*AA9M on PASC, *Nc*AA9C on CNC).

We employed the H_2_O_2_ sensor to quantitatively investigate the substrate specificity of the six LPMOs at standard conditions. Their TNs differ greatly between substrates (Figures 2A and S3). While *Nc*AA9C can act on a broad substrate spectrum (cellopentaose, a wide variety of hemicelluloses, including galactan and cellulose; Figures 2A, S10, Tables S6 and S7), the other tested C4-oxidizing LPMOs (*Nc*AA9M, *Gt*AA9B, *Hj*AA9B) are only active on xyloglucan and cellulose (Figures 2, S3, and S11–S14). Most interestingly, the tested C1-oxidizing LPMOs (*Nc*AA9F, *Pc*AA9D) only act on cellulose (Figures S3 and S15). None of the six LPMOs acts on xylan, arabinoxylan, mannan, or curdlan (Figure S10). Previous qualitative assessment of the substrate specificity of some of the LPMOs in this study, including further information on their known properties, is given in Table S1.

The concentration-dependent activity of *Nc*AA9C and *Nc*AA9M on soluble and dispersed substrates was studied in detail (Figure 2B–D). Substrate concentration-dependent TNs were fit to a hyperbola, allowing the determination of the apparent steady-state enzyme–substrate complex dissociation constant (*K*_M,app_) and maximal turnover numbers (T*N*_max,app_). For example, *Nc*AA9C acting on cellopentaose gives a *K*_M,app_ of 1200 ± 200 μM and a TN_max,app_ of 75 ± 5 s^–1^. Both constants are similar to previously reported values obtained with a discontinuous method.^20^*Nc*AA9C converts the unsubstituted β-glucans cellopentaose and glucomannan faster than xyloglucan but requires higher concentrations to reach saturation. This is reflected by a 5- and 21-fold higher *K*_M,app_, respectively (Figure 2C,D and Table S8). Interestingly, compared to *Nc*AA9C, *Nc*AA9M shows a similar TN_max,app_ for xyloglucan but a 3-fold lower *K*_M,app_ (Figure 2C,D and Table S8). *Nc*AA9C is clearly more suited to act on cellulosic substrates than *Nc*AA9M. For PASC, *Nc*AA9C, which carries a cellulose-binding domain, shows a 3-fold higher TN_max,app_ and a 1.5-fold lower *K*_M,app_ compared to *Nc*AA9M, which lacks a cellulose-binding domain. This difference is even more prominent for CNC particles where we found only a very minor activity with *Nc*AA9M, which did not allow the calculation of apparent kinetic constants (Figure S3). The *K*_M,app_ of *Nc*AA9C for CNC is much higher than for PASC (40 times) or hemicelluloses, showing that this crystalline material contains less productive binding sites compared to more amorphous substrates. Looking at the obtained data, in general, it becomes obvious that LPMOs are more efficiently acting on soluble substrates. This higher efficiency is not only reflected in higher TN values but also in higher residual activities (Figure S3), where the latter is not only due to the availability of productive binding sites (reflected by the variation of *K*_M,app_) but also due to higher substrate diffusibility.

The data discussed above allow a never-before-seen, reliable functional comparison of LPMOs acting on natural substrates. One notable feature derived from comparing C4-oxidizing LPMOs is the large impact of the carbohydrate-binding module (fitted TN_max,app_ in Figure 2C,D and Table S8; measured TN in Figure S3). While the soluble hemicellulose xyloglucan is converted at comparable rates by all four tested C4-oxidizing LPMOs (25–35 s^–1^), indicating similar copper-active site environments and reactivities, the TN for cellulosic substrates varies by up to an order of magnitude between the single-domain LPMOs (*Nc*AA9M, *Hj*AA9B, and *Gt*AA9B) and *Nc*AA9C, which features a type 1 carbohydrate-binding module. The kinetic mapping of substrate specificities described above was accompanied by measuring residual enzyme activities, which showed a substrate-dependent and LPMO-dependent variation in turnover stability as summarized in Figure S3. For example, the C4-oxidizing LPMOs show a higher turnover stability than C1-oxidizing LPMOs (*Nc*AA9F and *Pc*AA9D) when acting on PASC and CNC. The C4-oxidizing LPMOs are also more stable when acting on the soluble substrate xyloglucan.

### LPMOs Are Durable Peroxygenases

**Figure 3:**
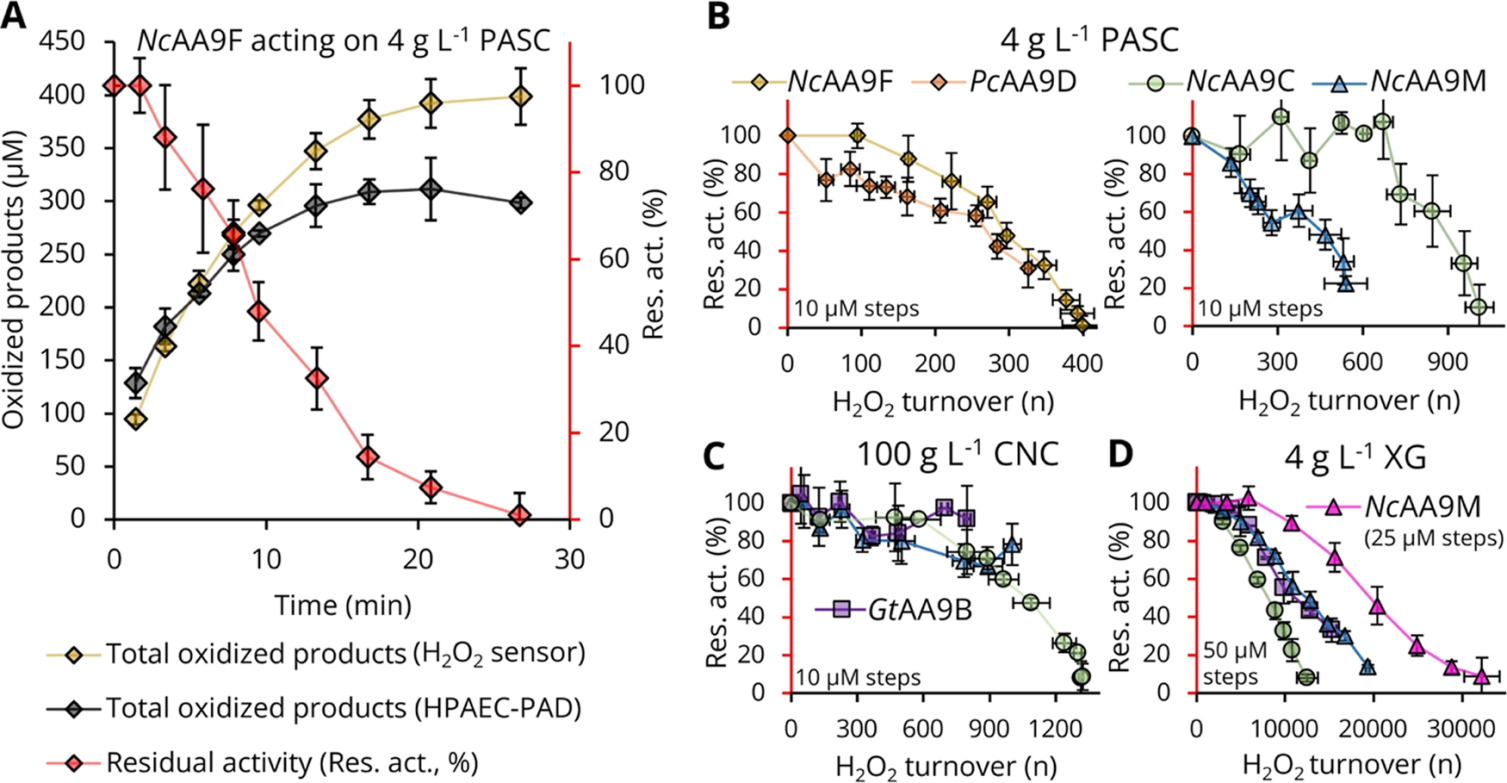
Determining the turnover stability of LPMOs. Reactions contained 5 mM ascorbate buffered to pH 6.0, 1 μM LPMO for PASC and CNC, and 50 nM LPMO for xyloglucan (XG). To determine TTNs of LPMOs for PASC and CNC, H_2_O_2_ was repeatedly titrated in 10 μM steps after consumption of the previous aliquot (Figures S16 and S18). To determine TTNs of LPMOs for xyloglucan, H_2_O_2_ was titrated in 25 or 50 μM steps (Figure S19). TTNs are summarized in Table S9. (A) Time course of *Nc*AA9F acting on PASC. The number of turnovers was calculated from the amount of titrated H_2_O_2_ (yellow diamonds), as well as from the off-line analysis of total oxidized products (gray diamonds, raw data in Figure S17 and Table S5) and compared to the residual activity (red diamonds). (B) Residual activity as a function of H_2_O_2_ turnovers for C1-oxidizing LPMOs (*Nc*AA9F and *Pc*AA9D, left panel) and C4-oxidizing LPMOs (*Nc*AA9C and *Nc*AA9M, right panel) acting on PASC. The H_2_O_2_ turnover and residual activities were extracted from the titration data sets of three independent repeats. The sampling of three independent experiments with slightly shifted titration and sampling times (Figures S16 and S18) necessitates horizontal error bars. (C) Residual activity as a function of H_2_O_2_ turnovers for C4-oxidizing LPMOs (*Nc*AA9C, green circles; *Nc*AA9M, blue triangles; *Gt*AA9B, purple squares) acting on CNC (raw data in Figure S16) and (D) residual activity in reactions with xyloglucan (raw data in Figure S19). The experiment for *Nc*AA9M was performed with both 25 μM (pink triangles) and 50 μM H_2_O_2_ (blue triangles) titrated concentrations; for the other two LPMOs, only 50 μM was applied.

Zooming in on turnover stability, we then used the H_2_O_2_ sensor technology to assess total turnover numbers (TTNs) for LPMOs. Since some of the LPMO reaction products (e.g., C4-oxidized sugars) are unstable and suitable standards are often not available, the assessment of TTNs with HPLC/MS methods is a difficult task. Multiple, subsequent titrations with H_2_O_2_ to a final concentration between 10 and 50 μM were performed to maintain the LPMO reaction over time and simultaneously monitor the residual LPMO activity. The reactions with C1-oxidizing *Nc*AA9F (Figure 3A; raw data in Figures S16 and S17) and *Pc*AA9D (Figure S18) were also monitored using HPAEC-PAD for the production of C1-oxidized products, and, again, there was a strong correlation between the amount of total oxidized products and the amount of consumed H_2_O_2_.

The TTNs for the LPMOs obtained for the cellulosic substrates PASC and CNC were in the order of 300–1300 cleavages per enzyme (Figure 3B–D and Table S9). One could expect that a combination of a low *K*_M,app_ and high TN, as is the case for xyloglucan (Figure 2C,D), would hamper off-pathway reactions and promote high TTNs. Indeed, reactions with xyloglucan resulted in TTNs that were 1–2 orders of magnitude higher compared to the TTNs obtained with PASC (Figure 3D and Table S9). The reduction of the H_2_O_2_ titration dosage from 50 to 25 μM H_2_O_2_ steps increased the TTN of *Nc*AA9M for xyloglucan from 19,300 to 32,200 (Figure 3D and Table S9), showing the importance of not overdosing H_2_O_2_ and illustrating that LPMOs may work very well at low H_2_O_2_ concentrations. The determined TTNs (Table S9) show that LPMOs are durable biocatalysts with high turnover stability if H_2_O_2_ concentrations are kept low and substrate concentrations are kept high.

### Multiple Influences of pH on LPMO Activity

**Figure 4:**
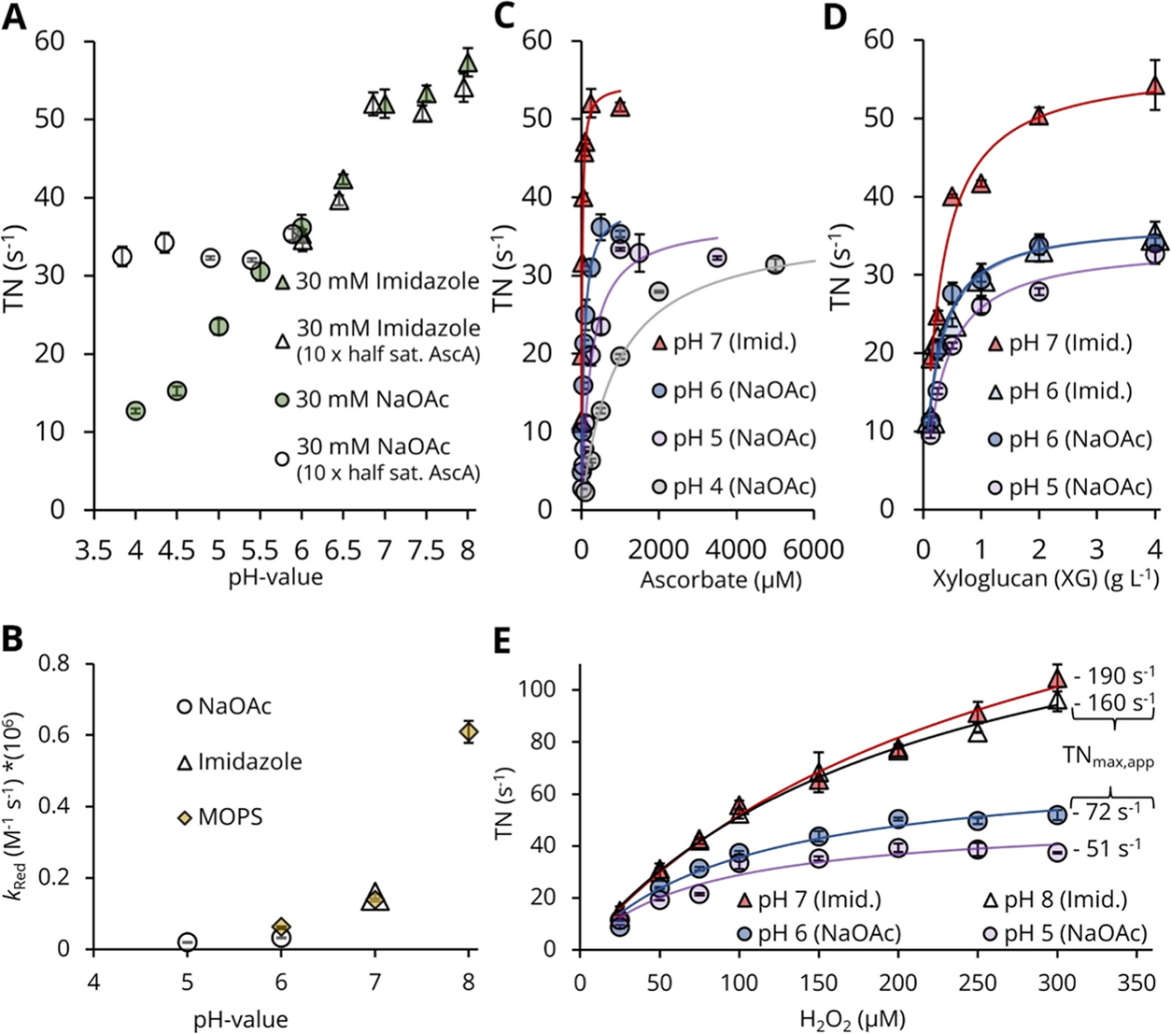
Factors governing *Nc*AA9C activity. All measurements were conducted with *Nc*AA9C in either 30 mM sodium acetate buffer (pH 4.0–6.0, dots) or 30 mM imidazole chloride buffer (pH 6.0–8.0, triangles) at 30 °C in triplicate, with 4 g L^–1^ of xyloglucan as a substrate. (A) pH profile of *Nc*AA9C with 500 μM ascorbate (green symbols) and a corrected pH profile with an ascorbate concentration (empty symbols) equalling ten times the pH-dependent half-saturating concentration shown in Table S10. The H_2_O_2_ concentration was 100 μM. (B) pH-dependency of the second-order rate of reduction of *Nc*AA9C by ascorbate in the absence of substrate, measured by stopped-flow spectrofluorometry in 50 mM sodium acetate, imidazole-HCl, or MOPS-HCl buffer at 30 °C. (C) Dependency of LPMO activity on the concentration of ascorbate at different pH values; the H_2_O_2_ concentration was 100 μM. (D) pH-dependent saturation kinetics for *Nc*AA9C with variation of the xyloglucan concentration; the H_2_O_2_ concentration was 100 μM. For the saturation kinetics experiments displayed in panels (D) and (E), the ascorbate concentration was 3 mM. (E) pH-dependent saturation kinetics for *Nc*AA9C with variation of the H_2_O_2_ concentration. The same data set is shown in Figure S22, including residual activities for each H_2_O_2_ concentration. Apparent kinetic constants derived from the data displayed in panels (C–E) are summarized in Table S10.

Building further on the acquired ability to resolve kinetic details of LPMOs, we studied the effect of pH on LPMO activity. To date, essentially nothing is known about the pH dependence of the LPMO peroxygenase reaction. Soluble xyloglucan was used to observe rates under a broad range of conditions (Figure 4 and Table S10). The activity of *Nc*AA9C shows a strong dependence on pH with a monotonic increase from pH 4.0 to 8.0 (Figure 4A). The low activity observed at acidic pH can be partially attributed to the increased protonation of ascorbic acid (p*K*_a_ = 4.1),^42^ which could hamper reductive LPMO activation. To substantiate the pH-dependency of the activation reaction, we performed fast kinetic experiments (Figures 4B and S20). The second-order rate constant for copper reduction increases exponentially with pH, from 20,000 M^–1^ s^–1^ at pH 5.0 to 600,000 M^–1^ s^–1^ at pH 8.0, indicating that the pH-dependency at low pH is dominated by a variation in the rate of reduction. In contrast, the second-order rate constant for the reoxidation of *Nc*AA9C–Cu(I) shows no pH-dependency (Figure S20). Notably, the obtained second-order rate constants for reduction and reoxidation at pH 7.0 are in line with data reported by Hall et al.^43^

The fast kinetic data are in accord with the pH-dependency of half-saturating ascorbate concentrations derived from steady-state analysis (Figure 4C and Table S10). After finding that the ascorbic acid/ascorbate concentration necessary to reach half-maximal activity is ∼50-fold higher at pH 4.0 than at pH 7.0 (890 and 17 μM, respectively), a reassessment of the pH profile using increased ascorbate concentrations showed essentially no pH-dependency of LPMO activity between pH 4.0 and 6.0 (TN = 30–35 s^–1^), while activity still increases with pH from 6.0 to 8.0 (TN = 35–55 s^–1^) (Figure 4A). Measurements of residual activities showed increased LPMO inactivation below pH 5.0, while turnover stability was essentially pH-independent above pH 5.0 (Figure S21). Further assessment of the pH-dependency of the kinetics of *Nc*AA9C acting on xyloglucan showed that substrate binding is essentially independent of the pH (Figure 4D) and steady-state experiments with varying xyloglucan concentrations resulted in T*N*_max,app_ values identical to those obtained when varying ascorbic acid (Table S10). Assessment of the effect of H_2_O_2_ on the pH-dependent activity (Figure 4E) revealed that the routinely used 100 μM H_2_O_2_ concentration is not saturating for *Nc*AA9C acting on xyloglucan.

### Low H_2_O_2_ Concentrations Limit Activity and Self-Inactivation

A closer look at the data for *Nc*AA9C acting on xyloglucan (Figures 4 and S22 and Table S10) shows that the TN_max,app_ values extrapolated for saturating H_2_O_2_ concentrations are among the highest ever reported for an LPMO (51 ± 5, 72 ± 6, 190 ± 18, and 162 ± 10 s^–1^ for pH 5.0, 6.0, 7.0, and 8.0, respectively; Table S10). Generally, a H_2_O_2_ concentration of 100 μM concentration was found to be optimal for the characterization of LPMOs, as it allowed for an acceptable rate of enzyme inactivation for the set of AA9 LPMOs studied. A closer look at the impact of the H_2_O_2_ concentration showed that in most cases, reactions could be speeded up by using higher concentrations at the expense of varying degrees of increased enzyme inactivation (Figures S22 and S23). For example, the H_2_O_2_ concentration necessary to reach half-maximal activity of *Nc*AA9C is approximately 100 μM at pH 5.0 and 6.0 and increases to 220 and 280 μM for pH 7.0 and 8.0, respectively (Figures 4D, S22, and Table S10). The saturation and inactivation behavior are strongly dependent on the substrate. For some LPMOs acting on soluble, and thus rapidly diffusing, xyloglucan, no apparent saturation was observed for H_2_O_2_ concentrations up to 300 μM and enzyme inactivation was slow; an example is *Gt*AA9B that lost only 18% of its activity compared to *Nc*AA9M that lost 51% of its activity after 6000 turnovers (Figure S23). On the other side of the spectrum, reactions with insoluble cellulose are slower and much more prone to enzyme inactivation, even at lower H_2_O_2_ concentrations (e.g., CNC; Figures S22 and S23). It would thus seem that protection against self-inactivation is naturally favored by not fully exploiting the catalytic potential of at least some LPMOs.

## Discussion

Application of this new electrochemical method will pave the way to study H_2_O_2_ consuming enzymes like peroxygenases and peroxidases, and probably also H_2_O_2_ producing oxidases, acting on polymeric, dispersed substrates that cannot be used in photometric or fluorimetric assays. The degradation of the most abundant biopolymers on Earth (cellulose, hemicellulose, chitin, lignin) depends on the action of a multitude of oxidoreductases, including many enzymes that produce or consume H_2_O_2_.^19^ Studying enzyme kinetics with heterogeneous substrates is often difficult or even impossible due to the lack of suitable methods and standards, and the present real-time detection method solves that problem. Not only does the rotating disc electrode-based sensor ignore optical dispersion such as reflected light but it also greatly minimizes mass transfer limitations that would otherwise reduce the availability of cosubstrates for substrate-bound enzymes. Importantly, it also allows the study of LPMOs acting on more realistic and biologically more relevant copolymeric substrates such as plant cell walls or putative not yet identified substrates such as microbial cell walls.^13−15^ As shown here, the method enables kinetic studies of LPMOs with greater speed and accuracy compared to previous methods, which will help unravel the catalytic landscape of these enzymes. Notably, the sensor system is available at a total purchasing cost of less than $20,000 and does not require special laboratory facilities.

The H_2_O_2_ sensor is relatively easy to prepare by electrochemical deposition of Prussian blue on a gold electrode and performs equally well in terms of sensitivity (220–260 nA μM^–1^ cm^–2^) compared to platinum-based electrodes or any newly developed H_2_O_2_ electrocatalyst (30–1700 nA μM^–1^ cm^–2^).^44−46^ The Nafion-coated Prussian blue electrocatalyst surpasses a standard platinum electrode by (i) a reduced influence of electroactive species in the matrix,^47^ (ii) its inactivity toward oxygen, which allows its use in aerated buffers,^38^ (iii) the good stability of the electrocatalyst in the measurement (as illustrated by the long-term experiments shown in Figure S19), and (iv) its low tendency for fouling by the adsorption of carbohydrates^48,49^ or lignin-derived polymeric material on the electrode surface,^50^ which is a common problem when working with lignocellulose samples. The Prussian blue-modified electrodes can be stored for up to a week. The response time of the rotating disc electrode in the electrochemical measurement cell is fast (<2 s) even in very concentrated solutions and suspensions, which is much better than the typical time of 10–200 s observed for static sensors in stagnant solutions.^38,51^ We show that the H_2_O_2_ sensor is working very well in high concentrations of up to 100 g L^–1^ of carbohydrates and lignin, shows no matrix effects, and maintains high accuracy together with a low limit of quantification in all tested conditions. This makes the sensor a suitable tool to monitor peroxidases and peroxygenases sensitively and even detect enzymes with a low activity. Most importantly, this sensor allowed the generation of a large set of kinetic constants for various LPMOs acting on soluble or dispersed substrates under physiologically relevant conditions.

Studies of six AA9 LPMOs acting on a variety of substrates showed measured TNs between 1.2 and 62.5 s^–1^, well comparable to results from the literature (4.0–124 s^–1^).^20,25,37^ The fitted T*N*_max,app_ value of *Nc*AA9C acting on xyloglucan is the highest ever reported (196 s^–1^) for an LPMO. The measured TNs with H_2_O_2_ are about 10^2^–10^3^ times higher compared to the rates observed in apparent monooxygenase reactions.^19,35^ Previous literature questioning the true peroxygenase nature of LPMO argued for a fast inactivation of the enzyme when using H_2_O_2_ as a cosubstrate.^52^ With the sensor, we could exactly determine the self-inactivation kinetics of different LPMOs in the absence and presence of substrate. On the one hand, an AA9-type LPMO can tolerate an average off-pathway turnover of 120 molecules of H_2_O_2_ before becoming inactivated; on the other hand, LPMOs can perform many more productive turnovers in the presence of substrate and H_2_O_2_. Using the H_2_O_2_ sensor, we measured TTNs much more precisely than with HPLC/MS-based methods since some of the LPMO reaction products (e.g., C4-oxidized sugars) are unstable^33,53^ and suitable standards are often not available. The TTN measurements show that the presence of a high concentration of a suitable substrate prevents the futile self-inactivating reaction pathway and that, under optimized reaction conditions, a total turnover number of at least 30,000 can be achieved. This shows that many, and likely all, LPMOs are efficient peroxygenases that are kinetically comparable to heme peroxygenases. For comparison, unspecific peroxygenases have reported TNs in the range of 30–1000 s^–1^ and TTNs between 40,000 and 100,000.^22^ An important finding of this study is that a high concentration of a good, rapidly converted substrate promotes turnover stability and increases the efficiency of ascorbate utilization, the latter reflecting the avoidance of off-pathway reactions that lead to the need for rereduction of the LPMO.

Besides revealing the kinetic features of LPMOs, this study investigated LPMOs in regard to substrate specificity and provided important functional insights. While substrate specificity of LPMOs has been investigated before,^9,54,55^ studies usually lack exact data for enzymatic activities. For example, we show that C1-oxidizing LPMOs are only active on cellulosic substrates, while all C4-oxidizing LPMOs have much higher activity on soluble hemicelluloses. As another example, the substrate affinity of C4-oxidizing LPMOs for soluble oligo- and polysaccharides can be clearly differentiated into LPMOs that do or do not tolerate xylose substitutions in the xyloglucan backbone.^56,57^ The data also clearly show the differences between LPMOs carrying a CBM (two-domain LPMOs) and single-domain LPMOs lacking a CBM. Two-domain LPMOs like *Nc*AA9C have higher TNs and a higher turnover stability due to a higher percentage of substrate-bound enzymes. Moreover, the study shows unequivocally that H_2_O_2_ is the kinetically relevant cosubstrate of LPMOs and verifies the reported stoichiometric ratio of 1 for each cleaved glycosidic bond.^21^

Prior to this study, only the pH dependence of reductant-driven LPMO reactions had been investigated,^35,58,59^ which basically means that the observed pH effects relate to reductant properties and not to LPMO properties and that nothing was known about the pH-dependency of the LPMO peroxygenase reaction. Using the sensor, we were able to assess the pH-dependency of the LPMO peroxygenase reaction under relevant reaction conditions. The electrochemical assay method allows a calibration of the H_2_O_2_ consumption at any pH between 4 and 8 and can thus generate accurate and precise data. The pH-dependency of LPMO activity results from two processes. The reductive activation efficiency of *Nc*AA9C by ascorbate monotonously increases with pH, while the H_2_O_2_ consumption clearly shows an alkaline optimum indicated by a strong increase between pH 6.0 and 8.0. The obtained H_2_O_2_ concentration to reach a half-maximal activity of *Nc*AA9C at pH 6.0 (105 μM) is in line with a recalculated *K*_M_,_H2O2_ of approximately 100 μM for reactions of *Nc*AA9C with cellopentaose (derived by combining kinetic data from two previous publications).^20,24^ In a biological context, the results of the kinetic analysis of *Nc*AA9C are remarkable in multiple ways. First, the alkaline pH-optimum of this LPMO is unexpected, considering that fungi tend to thrive at slightly acidic pH.^60,61^ Second, the H_2_O_2_ saturation data show that, at least for some LPMO–substrate combinations, the H_2_O_2_ concentrations required to reach maximum LPMO activity are higher than the expected low micromolar concentrations of H_2_O_2_ produced by wood-degrading fungi.^62^ It would thus seem that the catalytic potential of at least some LPMOs is not fully exploited in the natural environment. At the same time, reactions at lower pH and lower H_2_O_2_ concentrations are stable and have appreciable rates, which may be exactly what nature needs.

In conclusion, this study provides unparalleled insights into the catalytic properties of a widely spread group of powerful biomass-degrading interfacial redox enzymes. The electrochemical method provides a tool to map and discover new substrates for such enzymes and to kinetically characterize both productive and nonproductive (i.e., damaging) reactions. These results allow for the study of a plethora of known and putative biological functions of LPMOs, and other redox enzymes involved with H_2_O_2_, in biomass conversion and beyond.

## Experimental Procedures

### Chemicals

All chemicals used were of the highest purity available. Aqueous solutions were prepared in deionized water with an electrical resistivity of ≥18 MΩ cm at 25 °C. Cellobiose (C7252) was purchased from Merck. Cello-oligosaccharides and hemicelluloses were purchased from Megazyme (Wicklow, Ireland): cellotriose (O-CTR-50MG), cellotetraose (O-CTE-50MG), cellopentaose (O-CPE-20MG), β-glucan from barley (high viscosity, P-BGBH), β-glucans from barley (low viscosity, P-BGBL), glucomannan (GM) from konjac tubers (low viscosity, P-GLCML), xyloglucan from tamarind seeds (P-XYGLN), galactan from lupin seeds (P-GALLU), lichenan from icelandic moss (P-LICHN), arabinoxylan from wheat flour (medium viscosity, P-WAXYM), mannan (1,4-β-d-Mannan, P-MANCB), xylan from birchwood (partially acetylated, P-ACXYL), xylan from beechwood (P-XYLNBE-10G), and curdlan (P-CURDL). Phosphoric acid-swollen cellulose (PASC) was prepared according to a published protocol^63^ from microcrystalline cellulose (MCC) (*d* = 50 μm, 11365, Avicel PH-101, Merck). Crystalline nanocellulose (CNC, *d* = 10–20 nm × *l* = 300–900 nm, NG01NC0101) was purchased from Nanografi Nanotechnology (Ankara, Turkey). Kraft lignin (370959) and horseradish peroxidase (77332) were purchased from Merck.

### Instruments

Electrochemical measurements were performed using an Autolab potentiostat (PGSTAT101, Metrohm, Herisau, Switzerland) connected to a rotating disc electrode module (motor controller and rotating disc setup, AUT.RDE.S, Metrohm). Cyclic voltammetry (CV) and amperometric measurements at 100 mV vs SHE were performed in an electrochemical cell consisting of a three-electrode setup using a gold rotating disc electrode (RDE, RDE.AU50.S, *d* = 5 mm, Metrohm) as the working electrode, which was modified with Prussian blue films to increase the sensitivity and specificity toward H_2_O_2_ and suppress the O_2_ reduction reaction. A coiled platinum wire from BASi (West Lafayette, IN) was used as a counter electrode, and a Ag|AgCl electrode (3 M KCl, MF-2056, BASi) was used as the reference electrode. The reference electrode was used in combination with a glass double-junction (MF-2030, BASi) filled with 100 mM KCl to protect the reference electrode from poisoning by the complex substrate mixtures. The potential of the reference electrode was checked against the calomel lab master reference electrode (EF-1352, BASi) every week. All measurements were performed using a water-jacketed low volume cell (MR-1212, BASi) connected to an SE-12 heating circulator from Julabo (Seelbach, Germany) to maintain a constant temperature of 30 °C.

### Enzymes

All enzymes used in this study were recombinantly produced in *Pichia pastoris*, except *Hj*AA9B, which was produced by *H. jecorina* (anamorph *Trichoderma reesei*) and purified according to a published procedure.^64^ Three LPMOs from *N. crassa*, *Nc*AA9C, *Nc*AA9M, and *Nc*AA9F, were recombinantly produced using *P. pastoris* as the expression host and purified as described previously using a combination of hydrophobic interaction chromatography and anion-exchange chromatography.^34,65^ The same production procedure and two-step purification process were employed to purify an LPMO from *G. trabeum* (*Gt*AA9B)^56^ and an LPMO from *P. chrysosporium* (*Pc*AA9D).^66^ To ensure full copper saturation of the active site, all LPMOs were incubated for 1 h at 4 °C in the presence of a 3-fold molar excess of CuSO_4_. The residual Cu^2+^ was removed using desalting columns (HiPrep 26/10 Desalting, Cytiva) rebuffering the LPMO to 50 mM tris-HCl buffer, pH 7.0 containing 100 mM NaCl. This procedure did not result in a significant increase of activity, which demonstrates that the addition of CuSO_4_ to the medium is sufficient to produce these LPMOs in a fully functional state. Finally, a concentration step with centrifugal filter units from Sartorius (TVS15RH01, 10 kDa molecular weight cutoff) was applied. The purity of the enzyme preparations was verified by SDS-PAGE. Cellobiose dehydrogenase from *N. crassa* (*Nc*CDHIIA) was produced and purified as previously described.^67^ Details on the known properties of the various LPMOs, as well as their UniProt accession numbers, are provided in Table S1.

### Preparation of H_2_O_2_ Sensor Electrodes

Catalytic rates shown in this study were measured with a Prussian blue-modified gold rotating disc electrode (RDE), which is termed the H_2_O_2_ sensor in this study. Preparation of the H_2_O_2_ sensor started by cleaning the gold RDE by dipping it into 10 M NaOH for 1 min followed by rinsing thoroughly with deionized H_2_O. Next, the gold RDE was polished to a mirror finish with aqueous alumina particles (0.05 μm) on MicroCloth (Buehler, Lake Bluff, IL). Residual polishing particles were removed by immersing the electrode in 30 mL of deionized H_2_O in a sonicated water bath for 5 min. Afterward, the smooth surface of the gold RDE was gently roughened on a polishing pad (MicroCut Plain 1200/P2500; Buehler) to increase the surface area and improve the adhesion of the subsequently deposited Prussian blue film. Prussian blue was deposited on the electrode surface by performing cyclic voltammetry in a solution containing 1 mM FeCl_3_, 1 mM K_3_[Fe(CN)_6_], 0.1 M KCl, and 0.1 M HCl. Eight to 12 potential scans were performed between 600 and 900 mV vs SHE with a scan rate of 20 mV s^–1^ (Figure S1A) without rotating the RDE. The number of scans varied for each electrode as too many scans produced poor-quality Prussian blue-modified electrodes. Deposition was considered successful if one could observe a slightly greenish-blue film evenly covering the electrode surface. Subsequently, the Prussian blue-coated electrode was rinsed with deionized H_2_O and activated by running 20 potential scans between 160 and 590 mV vs SHE at a scan rate of 50 mV s^–1^ in a solution of 0.1 M HCl containing 0.1 M KCl (Figure S1B). To obtain H_2_O_2_ sensors with high sensitivity and a fast response time, a cutoff criterion was applied. H_2_O_2_ sensors with anodic peak current densities between 1200 and 2400 μA cm^–2^ were considered to perform well, whereas sensors with a higher current density were not used. Subsequently, the activated H_2_O_2_ sensor was again rinsed with deionized H_2_O, then dried in a stream of N_2_, and finally coated with Nafion (Product number: 70160, 5% in aliphatic alcohols, Merck, Darmstadt) by pipetting 7 μL of the undiluted Nafion solution onto the surface of the electrode, followed by an overnight curing step at room temperature (22 °C, 12–16 h). After the curing step, the H_2_O_2_ sensor was conditioned in 30 mM sodium acetate, pH 6.0, containing 100 mM KCl (standard working buffer), applying 20 cycles between 160 and 590 mV vs SHE at a scan rate of 50 mV s^–1^ (Figure S1B). Finally, the performance of the H_2_O_2_ sensor was assessed by determining the amperometric response at 100 mV vs SHE and at a 50 s^–1^ angular velocity (3000 rpm of the RDE) upon titrating five aliquots of H_2_O_2_ to a final concentration of 100 μM in standard working buffer containing 4 g L^–1^ of xyloglucan. H_2_O_2_ sensors showed a linear response up to 300 μM, and only H_2_O_2_ sensors with a sensitivity between 150 and 300 nA μM^–1^ cm^–2^ (typically 250 nA μM^–1^ cm^–2^; Table S2) were used for studying LPMO catalysis. As a second selection criterion, only H_2_O_2_ sensors with a response time below 2 s were used (Figure S2A). Typically obtained qualification data of H_2_O_2_ sensors are shown in Figure S2B,C. H_2_O_2_ sensor performance data for different substrates are shown in Table S2. The probability of producing sensitive and fast-responding H_2_O_2_ sensors, i.e., sensors meeting the selection criteria, was 90%. Since the H_2_O_2_ sensor is very stable, it can be employed in long-time measurements for up to 10 h (Figure S19) or used repeatedly for more than 100 short-time measurements.

### Preparation of Solutions for the H_2_O_2_ Sensor

An approximately 50 mM H_2_O_2_ stock solution was prepared by diluting a commercially available 30% (w/w) of H_2_O_2_ solution (Merck, Darmstadt) in deionized H_2_O in a ratio of 1:200. This 50 mM H_2_O_2_ stock was further diluted 1:5 in deionized H_2_O directly before each measurement series to obtain a concentration of approximately 10 mM. The accurate H_2_O_2_ concentration of this solution was determined directly before each measurement series in a 10 mm quartz cuvette using the molar absorption coefficient at 240 nm (ε_240_ = 43.6 M^–1^ cm^–1^) and was used for calculating exact concentrations, further dilutions, and injection volumes.

A 500 mM ascorbic acid stock solution was used for all measurements and prepared fresh every day. If the final ascorbic acid concentration in experiments exceeded 500 μM, the stock solution was prepared in the appropriate buffer and pH. Before the experiments, the 500 mM stock solution was diluted twice at 1:50 (1:2500 final dilution) to determine the ascorbic acid concentration using a 10 mm quartz cuvette and by measuring the absorption at 250.7 nm using a pH-independent molar absorption coefficient of 8250 M^–1^ cm^–1^.^68^ Typically, an absorption of 1.65 at 250.7 nm was found, in accordance with the expected value.

For all measurements, a 50 μM stock of the used LPMO was prepared in 30 mM sodium acetate buffer, pH 6.0 and 100 mM KCl. The LPMO concentration was determined by measuring absorption at 280 nm in 10 mm quartz cuvettes and using the following calculated molar absorption coefficients: *Nc*AA9C: ε_280_ = 46,910 M^–1^ cm^–1^, *Nc*AA9M ε_280_ = 51,715 M^–1^ cm^–1^, *Nc*AA9F: ε_280_ = 51,130 M^–1^ cm^–1^, *Gt*AA9B: ε_280_ = 55,475 M^–1^ cm^–1^, *Hj*AA9B: ε_280_ = 59,360 M^–1^ cm^–1^, *Pc*AA9D ε_280_ = 37,485 M^–1^ cm^–1^.

All substrates used within this study were either dissolved or suspended in 30 mM sodium acetate buffer, pH 6.0 containing 100 mM KCl as a supporting electrolyte, except when altering the pH. For pH-profiles, xyloglucan was used as substrate either dissolved in 30 mM sodium acetate buffer, pH 4.0–6.0, containing 100 mM KCl, or 30 mM imidazole chloride buffer, pH 6.0–8.0 containing 100 mM KCl. Cellopentaose (DP5) was dissolved at least 1 h before the measurement. Hemicelluloses were dissolved overnight on an orbital shaker at 30 °C. After determining the dry weight (g g^–1^) of the PASC stock suspension, a diluted PASC suspension of 8 g L^–1^ was prepared in 30 mM sodium acetate buffer, pH 6.0 containing 100 mM KCl and diluted further to 0.25–6 g L^–1^ in the same buffer. All cellulose substrates were suspended overnight on an orbital shaker at 30 °C. CNC was suspended overnight to concentrations of 10–100 g L^–1^ in 30 mM sodium acetate buffer, pH 6.0 containing 100 mM KCl. MCC was suspended overnight in a concentration of 100 g L^–1^ in 30 mM sodium acetate buffer, pH 6.0 containing 100 mM KCl. After dissolving or suspending the respective substrates, the pH was verified in each solution or suspension.

### H_2_O_2_ Sensor Experiments

The measurement of peroxygenase or off-pathway peroxidase-like activity consisted of three essential steps (Figure 1B):

(1) Each measurement was started by polarizing the Prussian blue-modified rotating disc electrode until a constant equilibrium current was reached, which was recorded for 60 s. This is termed the pre-experimental baseline. This baseline was used to correct for any drift of the signal during the measurement and is further discussed in the Data Processing section. Next, the H_2_O_2_ sensor was calibrated (i.e., generation of a standard curve) by titrating H_2_O_2_ in defined amounts (e.g., 5 × 20 μM H_2_O_2_ for an experiment with a 100 μM initial H_2_O_2_ concentration). The calibration of the sensor was done for every single measurement to exclude any systematic influence of a change in the sensitivity of the electrode. Upon the addition of H_2_O_2_, the system equilibrated within less than 2 s to a more negative current, which was recorded for 30 s to verify the stability of the system at the final H_2_O_2_ concentration.
(2) After calibrating the sensor, the reductant was added and the current signal was recorded for another 30 s to verify the stability of the signal in the presence of ascorbate. The substrate-dependent stability of the current signal in the presence of 100 μM H_2_O_2_ and 500 μM ascorbate is shown in Figure S10 and Table S7.
(3) Finally, the reaction was started by adding LPMO. Note that, when studying the LPMO activity in the presence of cellulosic substrates (PASC, CNC, and MCC), the LPMO was added to the reaction 30 s before the addition of ascorbate to allow the LPMO to bind before starting the reaction. After starting the reaction (by adding the LPMO, or, for cellulosic substrates, by adding the reductant), the signal was followed until reaching the level of the pre-experimental baseline (corresponding to 0 μM H_2_O_2_) and further recorded for another 30 s. The signal recorded during these final 30 s is termed the postexperimental baseline. If the residual LPMO activity (%) was to be quantified, a fresh dosage of H_2_O_2_ was immediately added once the postexperimental baseline was reached as illustrated in Figure 1C. This is further discussed in the Calculation of Turnover Numbers and Residual Activities section.

This procedure was applied to study any LPMO acting on any substrate, and parameters like pH, H_2_O_2_ concentration, ascorbate concentration, and substrate concentration were varied. All substrates and buffer solutions were incubated in a water bath set to 30 °C prior to performing measurements. The temperature of the substrate solution in the measurement cell was verified before starting a new experiment. After each measurement, the three electrodes as well as the measurement cell were thoroughly rinsed with deionized H_2_O and subsequently dried in an air stream.

### Standard Assay Conditions

The assay conditions to measure turnover numbers (TN) were optimized in a series of preliminary experiments. We used the following standard assay conditions unless otherwise noted: the optimized assays were performed by operating the amperometric H_2_O_2_ sensor at an applied potential of 100 mV vs SHE and an angular velocity of the RDE of 50 s^–1^. Every second, 12.5 data points were collected. The H_2_O_2_ sensor was operated in 4 mL of 30 mM sodium acetate buffer, pH 6.0, containing 100 mM KCl for improved conductivity in the temperature-controlled (30 °C) electrochemical cell. Substrate concentrations were 4 g L^–1^ for hemicelluloses, cellopentaose, or PASC and 100 g L^–1^ for CNC and MCC, and the initial concentration of H_2_O_2_ was 100 μM. LPMO concentrations varied according to the studied substrate and therefore will be indicated in figures and table legends. Typically, the following concentrations were used: 50 nM LPMO for hemicellulosic substrates, 100 nM LPMO for cellopentaose and PASC, and 200 nM LPMO for PASC, CNC, and MCC.

Reaction rates also depended on the concentration of the reductant, and thus it was important to determine saturating reductant concentrations. We, therefore, determined the amount of ascorbate required to achieve saturation for all studied LPMOs at pH 6.0 (Table S4). Figure S5 shows that saturation is achieved for all LPMOs acting on xyloglucan or PASC with 500 μM ascorbate but not for *Nc*AA9F and *Hj*AA9B acting on PASC and xyloglucan, respectively. For these two LPMOs, 2 mM ascorbate was used to achieve full saturation. Any deviation from these conditions is indicated in the figure and table legends. A control experiment with saturating amounts of cellobiose dehydrogenase from *N. crassa* (*Nc*CDHIIA) (Figure S6) to reduce *Nc*AA9C acting on 4 g L^–1^ of xyloglucan showed that the measured maximum catalytic rate of 31.9 ± 0.5 s^–1^ equals the rate obtained when using ascorbate as a reductant (31.6 ± 1.5 s^–1^; averaged over 3 different experiments using 50–200 nM *Nc*AA9C) (Table S3). This supports the notion that the experimentally obtained reaction rates are independent of the reductant.

To study the effect of different substrates, the cosubstrate H_2_O_2_, and the reductant ascorbate on catalysis, their concentrations were varied. The final substrate concentrations in experiments shown in Figure 2 were 0.125–8 g L^–1^ (DP5, hemicelluloses, PASC) and 10–100 g L^–1^ (CNC). The ascorbate concentration was varied between 0.005 and 12 mM to determine the pH-dependent concentration needed to achieve full saturation at pH 4.0–7.0 (Figure 4). To study the influence of pH (5.0–8.0) on catalysis by *Nc*AA9C at varying xyloglucan and H_2_O_2_ concentrations, shown in Figure 4, the ascorbate concentration was 3 mM, which is at least 10 times higher than the highest half-saturating concentration of ascorbate in the pH 5.0–8.0 range (at pH 5.0; Table S10). The H_2_O_2_ concentration was varied between 25 and 300 μM, while the xyloglucan concentration was kept constant at 4 g L^–1^. All measurements were performed in independent triplicate. The displayed H_2_O_2_ time traces were averaged over three independent measurements.

### Data Processing

Raw data obtained in amperometric measurements are current (nA) versus time (s). To exclude any systematic influence from current signal drift during the measurements, which would affect the H_2_O_2_ calibration and the calculation of initial rates, a system baseline was defined between the pre-experimental baseline and the postexperimental baseline. The pre-experimental baseline is reached once the current signal stays constants. Typically, this can be achieved by polarizing the electrode for 60 s at the start of the measurement. The postexperimental baseline is the constant current signal reached after performing a measurement. The small difference between these two baselines, i.e., the slope of signal change, is used to define a system baseline to correct the data set for a drift in signal.

Next, the currents for each H_2_O_2_ titration step after reaching equilibrium, i.e., a stable signal, were averaged over 20 s and used to calculate a linear calibration function (i.e., a H_2_O_2_ standard curve; R^2^ ≥ 0.9996; Table S2; see also Figure 1B in the main text). The slope and intercept of the calibration function were used to convert current (nA) into H_2_O_2_ concentration (μM) to obtain H_2_O_2_ time traces for LPMO-catalyzed reactions.

### Calculation of Turnover Numbers and Residual Activities

Two different LPMO reactions can be observed with the H_2_O_2_ sensor, one being the peroxygenase reaction that occurs in the presence of a suitable substrate (e.g., Figure 1B, green time trace). The same data set converted to H_2_O_2_ concentration vs time is shown in Figure 1C (green time trace). The peroxygenase reaction is characterized by fast and stable consumption of the H_2_O_2_ (in this case, 100 μM). The second reaction that happens in the absence of a bound substrate is an off-pathway reaction in which the reduced LPMO consumes H_2_O_2_ and becomes oxidized. This reaction leads to enzyme self-inactivation, and the consumption of H_2_O_2_ ceases after 20–50 s. This off-pathway reaction has also been termed as a peroxidase-like activity.^40^

To obtain initial rates (μM s^–1^), the slope of the initial linear phase of the H_2_O_2_ time trace was fitted with linear regression. Depending on the rate of the reaction, a time interval of 10–100 s was used to determine the initial rates. In Figure 1C, the first 20 s of the green time trace is fitted (1st cycle, linear fit as a purple dotted line). Typically, the linearity coefficients obtained for the initial phase of the peroxygenase reaction were R^2^ = 0.98 or higher, while the linear fit of the first 10 s of the off-pathway reaction resulted in an R^2^ of 0.92 or higher. For a 10 s time interval, 125 data points were obtained.

The initial rate (μM s^–1^) of an LPMO acting on any substrate is obtained as the total observed rate zero-order rate (*v*_obs_). Therefore, to obtain the true enzymatic rate (*v*_enz_) of an LPMO acting on a polysaccharide, two additional factors have to be considered: (i) the rate for the off-pathway consumption of H_2_O_2_ by the LPMO that takes place in the absence of, or, when not bound to, substrate (*v*_off-path_), and the progress of this reaction, if reported, is labeled explicitly in figures and tables (off-pathway activity, no or 0 g L^–1^ substrate). (ii) The rate of the substrate-dependent nonenzymatic consumption of H_2_O_2_ in the presence of ascorbate (*v*_sb_). Importantly, it is currently not possible to determine the extent to which the off-pathway activity of LPMO occurs in the presence of different substrates, and it is possible that this leads to an underestimation of the peroxygenase reaction rates in some cases. The rate for the off-pathway activity and the substrate-dependent background was routinely determined for each measurement series, for example, when conditions deviated from the standard assay conditions (e.g., different H_2_O_2_ and ascorbate concentrations and pH). The enzymatic activity of LPMO acting on a polysaccharide (*v*_enz_) was calculated from the total observed initial rate *v*_obs_ according to the equation
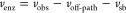


The data set used to determine the substrate-dependent background (*v*_sb_) for all studied substrates is shown in Figure S10, and the obtained slopes for *v*_sb_ are shown in Table S7. It is worth mentioning that, in general, the contribution of *v*_sb_ to *v*_obs_ is low compared to *v*_enz_, except for some hemicellulosic substrates. The reason for this effect of some of the hemicellulosic substrates is unknown but may be caused by the presence of metal ions. The rate of the off-pathway activity of LPMO (*v*_off-path_) is very similar for all studied LPMOs (as discussed in the main text), and its relative contribution to *v*_obs_ depends on the catalytic rate observed for a particular substrate. If *v*_obs_ was less than 4-fold higher than the standard deviation of *v*_off-path_, the studied LPMO was considered as inactive on a certain substrate. *v*_off-path_ was determined routinely for measurements with different H_2_O_2_ concentrations and used for the calculation of *v*_enz_. Since it must be expected that *v*_off-path_ in a reaction with substrate is lower compared to a reaction without substrate, the peroxygenase rates may be slightly underestimated by this conservative approach.

Finally, the fully corrected initial rate *v*_enz_ (μM s^–1^) was converted to a turnover number (TN, s^–1^) that is independent of the LPMO concentration. As an example, the obtained TNs for *Nc*AA9C catalysis shown in Figure 1C,D are reported in Table S3. Importantly, the values shown in Table S3, for both the peroxygenase activity and the off-pathway activity, show a linear dosage response to increasing LPMO concentrations, resulting in very similar TNs for reactions conducted with 50, 100, and 200 nM *Nc*AA9C.

This linear relationship allows us to determine the residual activity left after the first reaction cycle was completed. Residual activities were determined by adding the same amount of H_2_O_2_ a second time to the reaction cell once the first cycle was fully completed. The first cycle of the experiment is completed once the amount of H_2_O_2_ present, in the case of Figure 1C 100 μM, was consumed and the postexperimental baseline (0 μM H_2_O_2_) was reached. After starting the second cycle by adding 100 μM H_2_O_2_, the progress of the reaction was recorded until the postexperimental baseline (0 μM H_2_O_2_) was reached again. The time trace of 50 nM *Nc*AA9C acting on 4 g L^–1^ of xyloglucan shown in Figure 1C shows the linear fit of the initial rates of the first cycle (20 s fit interval in purple) and the second cycle (20 s fit interval in red). Residual activities were calculated from the ratio of the initial rates for the first and second reaction cycles and are reported in %. The observed rates (*v*_obs_) for both cycles were only corrected with the substrate-dependent background (*v*_sb_) and not with the rate for the off-pathway activity of LPMO because it is not possible to determine the rate for this reaction after the first reaction cycle was completed. The obtained residual activity for the experiment shown in Figure 1C is 77% (note that residual activities may approach 100% if lower amounts of H_2_O_2_ are used, as shown by other experiments reported here). The progress and rate of the off-pathway reaction of LPMOs, if reported, are labeled explicitly in figures and tables (off-pathway activity, no or 0 g L^–1^ substrate).

### Measurement of Total Turnover Numbers

To determine the total turnover number (TTN) of LPMOs, the measurements were performed at a working electrode potential of 100 mV vs SHE and an angular velocity of the RDE of 50 s^–1^ in 4 mL buffer at 30 °C. The concentration of the reductant ascorbate was 5 mM. If the substrate was 4 g L^–1^ of xyloglucan, 50 nM LPMO was added and H_2_O_2_ was titrated in 50 μM steps (for *Nc*AA9M also, 25 μM steps were used). For PASC (4 g L^–1^) and CNC (100 g L^–1^), 1 μM LPMO was added and H_2_O_2_ was titrated in 10 μM steps. The frequency of titration steps depended on the rate of H_2_O_2_ consumption. As shown in Figures S16, S18, and S19, H_2_O_2_ was titrated again to the reaction once the baseline (i.e., 0 μM H_2_O_2_) was reached, indicating full consumption of the substrate added in the previous titration step. When samples were collected during the titration experiment to quantify oxidized products off-line using HPAEC-PAD analysis (done for *Nc*AA9F and *Pc*AA9D), the reaction volume was increased to 12 mL to be able to sample 500 μL of aliquots up to 10 times during the reaction. In this case, the amount of H_2_O_2_ titrated was recalculated after each sampling to maintain 10 μM additions. The enzymatic reaction in the collected samples was stopped by incubating at 90 °C for 10 min. All measurements were performed in independent triplicate; however, the displayed titration raw data in Figures S16, S18, and S19 are from a single experiment.

### Calculation of Total Turnover Numbers

The same data processing procedure, as described above for determining turnover numbers, was applied to convert the obtained raw data during total turnover experiments into H_2_O_2_ consumption over time. The data were recorded every 0.2 s (5 data points per second). The total turnover numbers were calculated by summing up the amount of H_2_O_2_ consumed during consecutive titration steps, i.e., counting consecutive H_2_O_2_ injections until the consumption of cosubstrate leveled off. This data set was corrected with the substrate-dependent background consumption of H_2_O_2_. For each individual titration step, the time interval to reach the baseline was calculated and subsequently the background consumption of H_2_O_2_ in this time window was subtracted from the individual titration steps. The shown data sets for total turnover numbers therefore include productive and futile turnovers, eventually leading to self-inactivation. The residual activities were estimated based on fitting the short initial slopes of individual titration steps, taking the substrate-dependent background into account, as described in the Calculation of Turnover Numbers and Residual Activities section. The residual activity (%) was calculated using the initial rate derived from the first titration step as 100%. Residual activities were determined for approximately every fifth titration step, except for the experiments using xyloglucan, where the residual activity for each step was calculated.

### Validation of the H_2_O_2_ Sensor

The performance of the H_2_O_2_ sensor was validated by comparison with HPAEC-PAD measurements of product formation, using two experimental approaches. In one approach, the consumption of cellopentaose by *Nc*AA9C was quantified and used to calculate the stoichiometry of the substrate and cosubstrate (H_2_O_2_) consumption. In the other, experimentally more challenging approach, the formation of C1-oxidized products on PASC by *Nc*AA9F (C1-oxidizing) and *Pc*AA9D (C1-oxidizing) was quantified and compared to H_2_O_2_ consumption.

The correlation between cellopentaose consumption and H_2_O_2_ consumption was measured in conversion experiments, with *Nc*AA9C acting on cellopentaose at different concentrations (0.1, 0.2, 0.4, 1.0 mM), which were stopped after 130 s (1 mM) or 200 s by incubating a 100 μL aliquot of the reaction at 90 °C for 10 min. Before adding ascorbate (500 μM), a sample containing all other components of the reaction (100 nM *Nc*AA9C, 0.1–1 mM cellopentaose and 100 μM H_2_O_2_) was retrieved to benchmark the starting amount of cellopentaose present in each of the reactions. This initial cellopentaose concentration was used to calculate the amount of cellopentaose consumed. Before measuring cellopentaose concentrations, samples from the reactions with the lower cellopentaose concentrations of 0.1 and 0.2 mM were diluted 10-fold, whereas samples from reactions with the higher cellopentaose concentrations of 0.4 and 1 mM were diluted 50-fold. The amount of residual cellopentaose present in the reactions was quantified using a linear standard curve covering the 6.0–36.2 mM (5–30 mg L^–1^) concentration range. The decrease in the cellopentaose concentration was correlated with the consumed H_2_O_2_ measured with the H_2_O_2_ sensor, and from that, the cellopentaose:H_2_O_2_ stoichiometry was calculated. All analyzed samples were directly obtained from individual measurements with the H_2_O_2_ sensor, and the average and standard deviation were calculated from independent triplicate.

The formation of C1-oxidized products generated in TTN experiments with *Nc*AA9F or *Pc*AA9D acting on PASC was measured in 10 subsequently taken 500 μL samples retrieved from a total reaction volume of 12 mL. Directly after sampling, the LPMO was inactivated by incubation at 90 °C for 10 min. Exemplary sampling points are indicated in some of the figures (Figures S16 and S18). Control samples were obtained from the same reaction by sampling before the addition of ascorbate; these samples were incubated at 30 °C for the time of the experiment followed by enzyme inactivation by incubation at 90 °C for 10 min. The control samples, analyzed as described below, contained abundant native products and only very minor amounts of oxidized products. To quantify the total amount of C1-oxidized products, 25 μL of the collected samples were diluted to a final volume of 100 μL with 30 mM sodium acetate buffer pH 6.0 and subjected to treatment with *Thermobifida fusca* Cel6A to depolymerize the remaining cellulose and degrade soluble oxidized cello-oligomers. All samples were incubated with 5 μM purified *Tf*Cel6A^69^ at 40 °C for 48 h in sealed Eppendorf tubes and subsequently analyzed using HPAEC-PAD. *Tf*Cel6A degrades the insoluble cellulose, which contains oxidized sites, and converts native and oxidized cello-oligosaccharides to shorter native and oxidized oligomers and glucose. The formed oxidized products end up as a mixture of DP2ox and DP3ox, which are summed up to total oxidized products, representing soluble and insoluble oxidized products. All analyzed samples were obtained directly during the continuous H_2_O_2_ titration experiments done to determine the total turnover numbers. Thus, we can directly correlate the amount of H_2_O_2_ productively used to form C1-oxidized products in the same reaction. All samples were generated as independent triplicate.

### HPAEC-PAD Analysis of Cellopentaose and C1-Oxidized Degradation Products

Cellopentaose as well as C1-oxidized cellobiose and cellotriose were quantified using HPAEC-PAD performed as described recently.^10,20,36^ We used a Dionex ICS5000 system (Dionex, Sunnyvale, CA) equipped with a CarboPac PA200 analytical column (3 × 250 mm) and a CarboPac PA200 guard column (3 × 50 mm). For the analysis of C1-oxidized oligomers, we used the following program: eluent A consisted of 0.1 M NaOH, and analytes were eluted using a stepwise 26 min gradient of increasing amounts of eluent B (0.1 M NaOH + 1 M sodium acetate) as described by.^36^ The following program was applied using a flow-rate of 0.5 mL min^–1^: 0–5.5% B over 3 min, 5.5–15% B over 6 min, 15–100% B over 11 min, 100–0% B over 6 s, 0% B over 6 min. For analysis of cellopentaose, we used the following eluents: eluent A consisted of 0.150 M NaOH, and analytes were eluted with a flow-rate of 0.5 mL min^–1^ using a linear gradient of increasing amounts of eluent B (0.150 M NaOH + 0.5 M sodium acetate). The following program was applied using a flow-rate of 0.5 mL min^–1^: 0–28.8% B over 25 min, 28.8–100% B over 6 s, 100% B over 5 min, 100–0% B over 6 s, 0% B over 10 min. Chromatograms were analyzed using the Chromeleon 7.0 software (Thermo Fisher Scientific, Waltham, MA). Five cellopentaose standards between 5 and 30 mg L^–1^ (6.0 and 36.2 μM) were used for quantitation. Additional standards were 20 mg L^–1^ of cellobiose (DP2) and 20 mg L^–1^ of cellotriose (DP3), as well as in-house-prepared standards for the C1-oxidized dimer, cellobionic acid, and trimer, cellotrionic acid, produced as described by.^70^ C1-oxidized standards were used in the following concentration range: 2.5–100 μM. In brief, these standards were generated by incubating 0.1 g L^–1^ of cellobiose or cellotriose with 1 μM cellobiose dehydrogenase from *Myriococcum thermophilum* (*Mt*CDH; GenBank ID EF492052.3,^71^) in 50 mM sodium acetate buffer, pH 5.0, at 40 °C for 20 h.

### Fast Kinetic Measurements with a Stopped-Flow Spectrofluorimeter

An SFM4000 stopped-flow equipped with an MOS 200 M dual spectrophotometer (BioLogic Science Instruments, Seyssinet-Pariset, France) in a fluorescence mode was used to investigate the reduction and reoxidation of the copper site in *Nc*AA9C. The feasibility of using stopped-flow fluorescence measurements to determine first-order reaction rates for the reduction of LPMO-Cu(II) to LPMO-Cu(I), and vice versa, reoxidation of LPMO-Cu(I) in the presence of H_2_O_2_ were demonstrated by Bissaro and colleagues.^28,70^ Stopped-flow fluorescence measurements were conducted using an excitation wavelength of 280 nm (λEx), and emitted light was collected above 320 nm (λEm). The reduction of the active site from Cu(II) to Cu(I) can be performed with ascorbate or l-cysteine. We used ascorbate in two-syringe single mixing experiments aimed at determining reduction rates and l-cysteine in three-syringe dual mixing experiments aimed at measuring the rate of reoxidation by H_2_O_2_. The stopped-flow apparatus was operated with temperature control at 25 °C using only solutions and materials that had been prepared in an anaerobic chamber. All solutions were purged with N_2_ for 1 h and subsequently stored in a Whitley AT95TG anaerobic workstation (Don Whitley Scientific, West Yorkshire, U.K.) for at least 16 h before the experiment, together with all labware used. The lid on these solutions was loosened once moved to the anaerobic chamber to allow for gas exchange. The LPMO solution was gently sparged with N_2_ for 2 min in a 50 mL tube just before moving the enzyme to the anaerobic chamber. Oxygen-free buffer was used to flush the stopped-flow apparatus and to remove air bubbles. The reactants used in the stopped-flow experiments (LPMO, ascorbate, and H_2_O_2_ solutions with different concentrations) were prepared in sealed 5 mL syringes in the anaerobic chamber before being transferred to the stopped-flow syringe handling unit. Apparent first-order rates for reduction were obtained by mixing 5 μM *Nc*AA9C with increasing concentrations of reductant (25–1600 μM) and subsequently fitting the measured time traces to a single exponential function. To maintain the pH in these solutions, the ascorbate stock solution and LPMO stock solution were prepared in buffers. A 50 mM sodium acetate buffer was used in the range of pH 5.0–6.0, and a 50 mM imidazole chloride buffer was used in the range of pH 6.0–8.0. For determining the second-order rate of the reoxidation reaction, the LPMO-Cu(I) state was formed using the delay line of the stopped-flow in sequential mode by mixing equimolar amounts of LPMO-Cu(II) with l-cysteine (5 μM final concentration for 30 s). This was carried out in a 2.5 mM imidazole chloride buffer at pH 7.0 to allow for a quick pH-jump to the respective pH to determine pH-dependent reoxidation rates. Next, the LPMO-Cu(I) from the delay line was mixed with different H_2_O_2_ solutions in the concentration range of 25–800 μM to determine the second-order reoxidation rate. The used H_2_O_2_ solutions were prepared in 100 mM sodium acetate buffer, pH 5.0–6.0 or 100 mM imidazole chloride buffer, pH 6.0–8.0 to yield a final buffer concentration of 50 mM. For pH 7.0, one experiment using 100 mM MOPS buffer was included.

## Data Availability

All data are provided in the manuscript and the Supporting Information.

## Supporting Information Available

Figures, tables, and references providing additional data on sensor calibration, response time, enzyme activities, substrates, cosubstrates, turnover stability, and residual activity (PDF)
