# Supplementary material for: Electrochemical Monitoring of Heterogeneous Peroxygenase Reactions Unravels LPMO Kinetics
Source: ACS Catal. 2024 Jan 10;14(2):1205–19. doi: 10.1021/acscatal.3c05194 (PMC10804366; doi:10.1021/acscatal.3c05194)
Supplement: Supplementary file 1 — cs3c05194_si_001.pdf [file cs3c05194_si_001.pdf]

Supporting Information for

Electrochemical monitoring of heterogeneous peroxygenase reactions unravels LPMO kinetics

Lorenz Schwaiger[1], Florian Csarman[1], Hucheng Chang[1], Ole Golten[2], Vincent G.H. Eijsink[2]
and
Roland Ludwig[1]*

[1] University of Natural Resources and Life Sciences, Vienna, Department of Food Science and Technology, Institute of Food Technology, Muthgasse 18, 1190 Vienna, Austria

[2] Norwegian University of Life Sciences (NMBU), Faculty of Chemistry, Biotechnology and Food Science, P.O. Box 5003, 1432 Ås, Norway

*Corresponding author: roland.ludwig@boku.ac.at

This PDF file includes:

List of Abbreviations	page	2
Supplementary Figures S1 to S23	page	3
Supplementary Tables S1 to S10	page	35
Supplementary References	page	47

List of Abbreviations

AA9	auxiliary activity family 9 (fungal lytic polysaccharide monooxygenases)
Ag|AgCl	silver|silver chloride reference electrode
CDH	cellobiose dehydrogenase
CMC	carboxymethyl cellulose
CNC	crystalline nanocellulose
CV	cyclic voltammetry
*Gt*AA9B	*Gloeophyllum trabeum* AA9 (UniProt ID: F8T947)
*Hj*AA9B	*Hypocrea jecorina* AA9 (UniProt ID: Q7Z9M7)
LPMO	lytic polysaccharide monooxygenase (in this text typically a member of the AA9 family)
MCC	microcrystalline cellulose
*Nc*AA9C	*Neurospora crassa* AA9 (UniProt ID: Q7SHI8)
*Nc*AA9F	*Neurospora crassa* AA9 (UniProt ID: Q8WZQ2)
*Nc*AA9M	*Neurospora crassa* AA9 (UniProt ID: Q7SA19)
*Nc*CDH	*Neurospora crassa* CDHIIA (class II CDH, UniProt ID: Q7RXM0)
PASC	phosphoric acid swollen cellulose
*Pc*AA9D	*Phanerochaete chrysosporium* AA9 (UniProt ID: H1AE14)
RAC	regenerated amorphous cellulose
RDE	rotating disc electrode
SHE	standard hydrogen electrode
*Tf*Cel6A	cellobiohydrolase 6A from *Thermobifida fusca* (UniProt ID: P26222)
TN	turnover number
TTN	total turnover number

Supplementary Figures

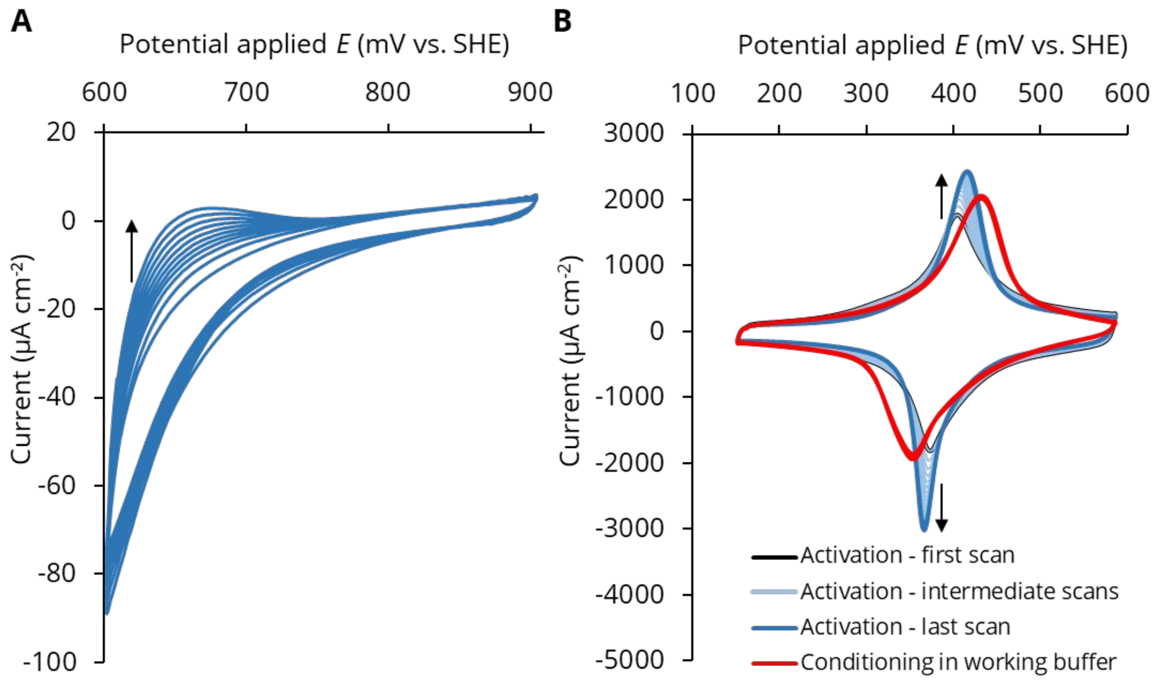

Figure S1. Preparation of Prussian blue-modified gold electrodes via electrochemical deposition. (**A**) The Prussian blue layer was deposited on a gold rotating disc electrode (RDE) in a solution containing 1 mM $FeCl_3$, 1 mM potassium ferricyanide ($K_3[Fe(CN)_6]$), 100 mM KCl and 100 mM HCl by 12 potential sweep cycles between 600 and 900 mV vs. SHE at a scan rate of 20 mV s^{-1}. The potential window used to deposit Prussian blue was chosen above the redox potential (380 mV. vs SHE) of the catalyst to facilitate a more gentle deposition process, as reported previously by [38,39]. The formation of a small peak at around 650 mV vs. SHE (indicated by an arrow) shows that deposited Prussian blue is being oxidized, i.e. it shows the onset of the anodic peak displayed in panel B. Note that, the obtained current densities during deposition (compared to activation in panel B) are two orders of magnitude lower because the cycling is performed at a much higher potential compared to the redox potential of the catalyst. (**B**) Activation of the deposited Prussian blue layer by cycling the RDE 20 times (see the Experimental Procedures section for further explanation) between 160 mV and 590 mV vs. SHE at a scan rate of 50 mV s^{-1} in a solution containing 100 mM KCl and 100 mM HCl. After activation, the H_2O_2 sensor was dried with N_2 and coated with a Nafion layer. The H_2O_2 sensor was stored overnight at ambient atmosphere (25 °C, covered with a glass beaker) and then conditioned in the standard assay buffer under the same conditions used during the activation procedure (scan rate 50 mV s^{-1}, 50 cycles; red voltammograms).

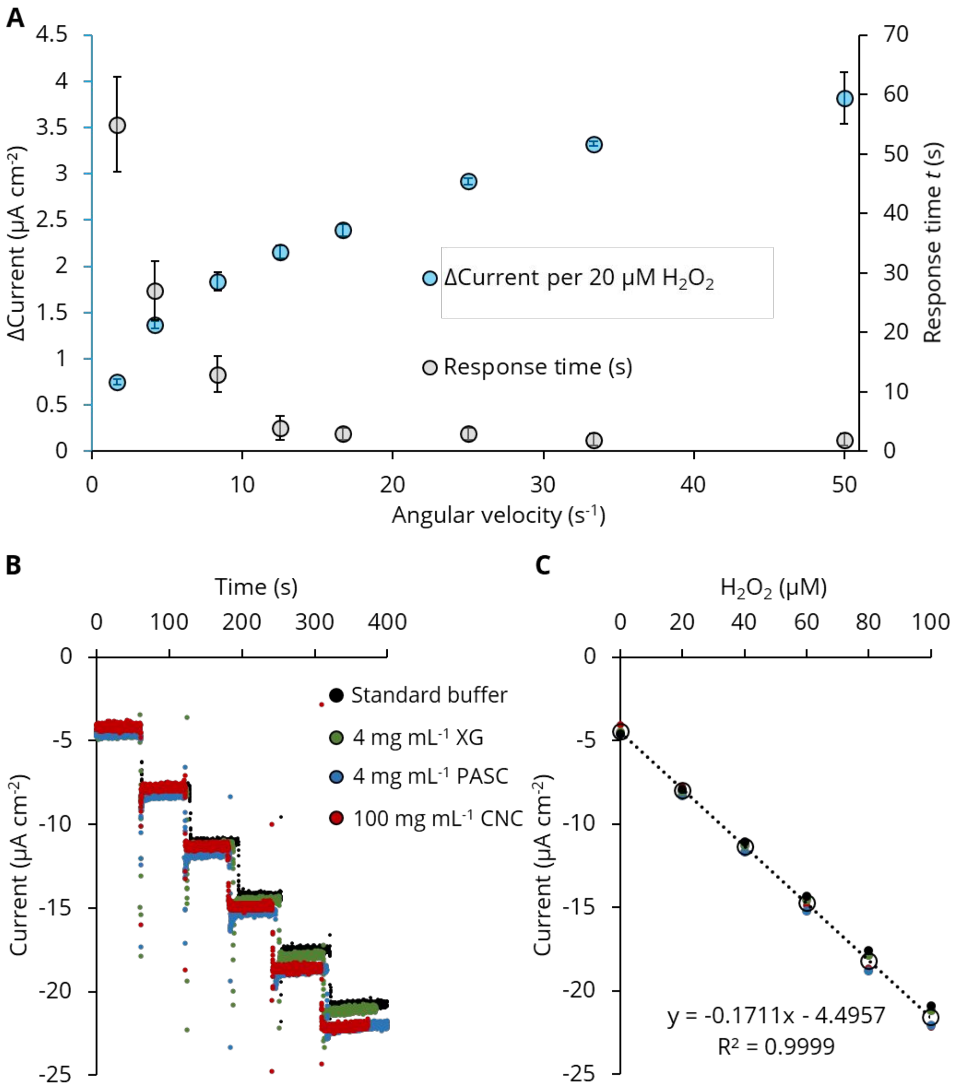

Figure S2. Performance characteristics of the H_2O_2 Sensor. (**A**) Current response (blue) and response time (>95% signal transition completed after H_2O_2 addition, gray) are plotted against the angular velocity of the electrode (s^{-1}). The experiment was performed in the presence of 4 g L^{-1} xyloglucan dissolved in 30 mM sodium acetate buffer, pH 6.0 containing 100 mM KCl. The data points of current response and response time were extracted after titrating 20 µM H_2O_2 into the system and waiting for approximately 100 s to average the current. As the angular velocity increases, the current response increases and the response time of the sensor decreases. All other experiments were performed at an angular velocity of 50 s^{-1} (3000 rpm) for optimal sensor performance. (**B**) Comparison of H_2O_2 sensor response in four different matrices (substrates resulting in a low or high viscosity solution or suspension). The buffer was 30 mM sodium acetate, pH 6.0, containing 100 mM KCl as supporting, inert electrolyte. xyloglucan, PASC, or CNC were added to test matrix effects. H_2O_2 was injected 5-times in 20 µM steps reaching a final concentration of 100 µM. These experiments show that the fast response time of the sensor is maintained in different matrices. The equilibrium current is reached within 2 s and recorded for 65 s to obtain the calibration function. The calibration raw data also show that the sensitivity of the electrode may depend on the substrate being studied, so each measurement was calibrated to exclude any influence of a changed sensitivity on the measured initial rates. (**C**) Currents from panel B and currents derived from three additional identical independent

measurements were averaged over 20 s and used to generate calibration cures. These curves show minimal differences in sensitivity between buffer, soluble xyloglucan and the insoluble cellulose substrates (PASC and CNC). From the obtained linear calibration functions, the sensitivity and the limit of detection for H_2O_2 sensors in different matrices was calculated. Sensitivities were calculated from the slope of the calibration function (nA μM^{-1} cm^{-2}) and the limit of quantitation was calculated by dividing 10x the standard deviation of the baseline (nA cm^{-1}, 0 μM H_2O_2) with the sensitivity of the electrode. Exemplary calibration data from a measurement series with different xyloglucan, PASC and CNC concentrations is given in **Table S2**.

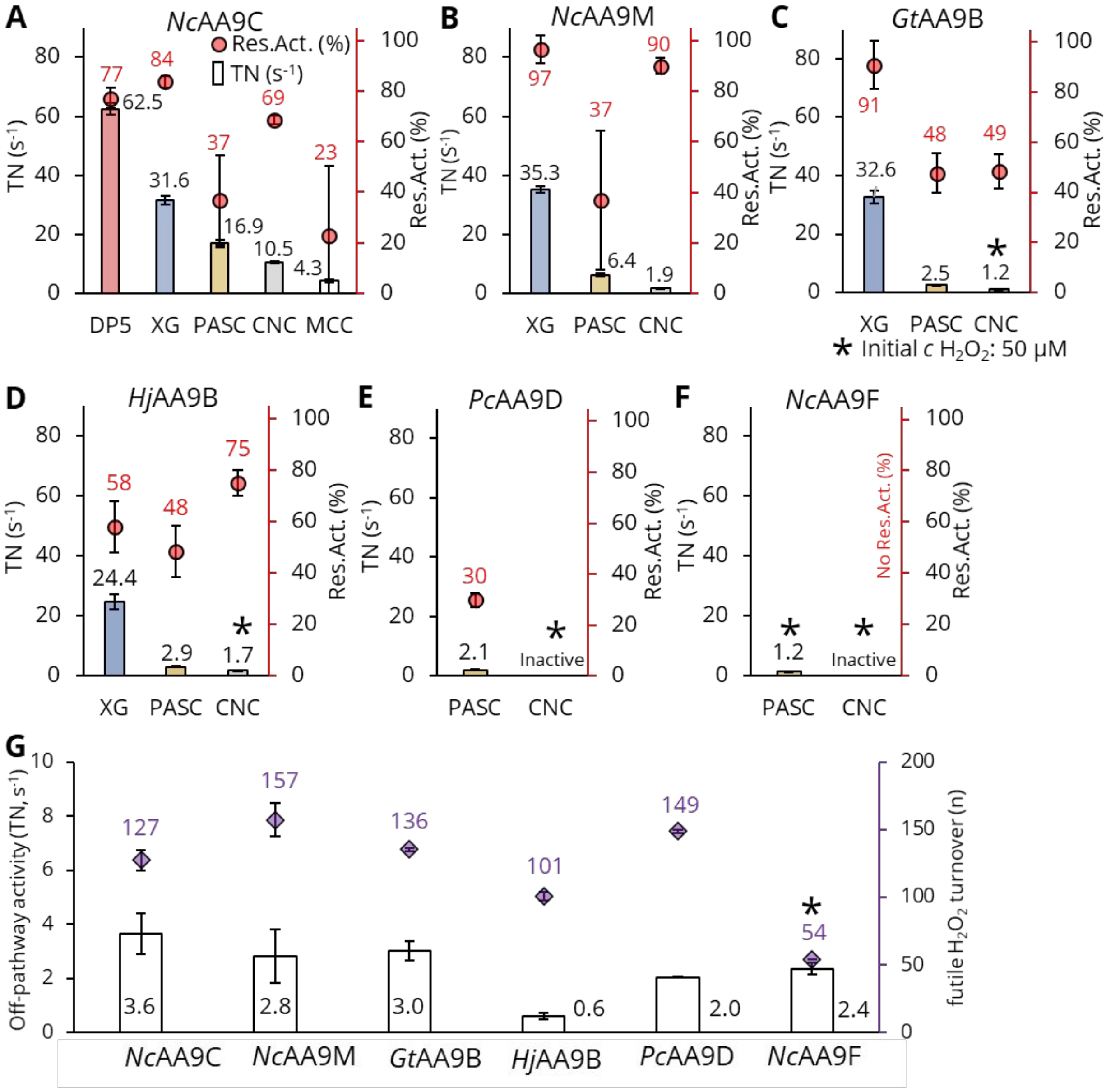

Figure S3. Substrate-dependent activity (TN, s^{-1}) and stability (Res. Act., %), off-pathway activity and futile H_2O_2 consumption (n) of the studied LPMOs. All measurements were performed using standard assay conditions as defined in the section "Standard assay conditions" in Materials and Methods and were performed in independent triplicates. For soluble substrates (xyloglucan and glucomannan) the LPMO concentration was 50 nM, for celluloses 200 nM. The rates for the off-pathway activity of LPMOs shown in panel G were determined using 200 nM LPMO. Exemplary raw data are shown in **Figures S7A, S11, S13-15**. Data obtained from experiments performed under other conditions than in the standard assay are indicated by an asterisk. For these measurements, the initial H_2O_2 concentration was 50 μM, whereas all other measurements were conducted with 100 μM H_2O_2.

The turnover numbers and residual activities were calculated as defined in the "Calculation of turnover numbers and residual activities" in the materials and methods section. In short, the observed initial initial rate (v_{obs}) was corrected with the substrate-dependent background (v_{sb})

and the rate for the peroxidase-like off-pathway activity ($v_{off\text{-}path}$). The peroxidase-like activity was only corrected for the v_{sb} measured in the buffer. For the determination of residual activities, once the initial amount of H_2O_2 added to the reaction was fully consumed, indicated by the H_2O_2 time trace reaching the baseline (0 µM H_2O_2), 100 µM H_2O_2 was added a second time. This second time trace was recorded until the baseline was reached again. The exact time of the second addition of H_2O_2 depended on the progress of the H_2O_2 time trace. For the calculation of residual activities, the v_{obs} for the first and second H_2O_2 addition was only corrected with the background for the respective substrate (v_{sb}) followed by calculating the ratio of the corrected first and second initial rates. Residual activities (%), as shown here, reflect the amount of activity left after consuming 100 µM H_2O_2 (exceptions are marked with an asterisk; here only 50 µM were consumed). (**A-F**) **Comparison of reaction rates and residual activities of LPMOs acting on different substrates.** In the case of studying catalysis in the presence of CNC and/or PASC, *Gt*AA9B, *Hj*AA9B, *Pc*AA9D and *Nc*AA9F showed rapid inactivation at 100 µM H_2O_2. Therefore, a lower concentration of H_2O_2 (50 µM, marked with an asterisk, panels **C-F**) was used. However, fast inactivation, even at 50 µM H_2O_2, did not allow for the determination of reaction rates for *Pc*AA9D (**E**) and *Nc*AA9F (**F**) acting on CNC. It is known that both LPMOs are active on crystalline cellulose substrates, but the observed initial rates (v_{obs}, including substrate background and off-pathway activity) were below the limit of the analysis, which was defined as the total observed rate (v_{obs}) being at least 4-times higher than the standard deviation of the rate of the peroxidase-like activity ($v_{off\text{-}path}$). Underpinning fast inactivation, the progress of the reaction started to level off well before the H_2O_2 time trace reached the baseline. Therefore, these LPMOs are marked as inactive on CNC. In the case of *Nc*AA9F acting on PASC (**F**), v_{obs} was 18-times higher than the standard deviation of the peroxidase-like activity ($v_{off\text{-}path}$) and the baseline was reached after 200 s, but the rate for the second reaction cycle was equal to the substrate-dependent background (v_{sb}) measured for PASC, therefore no residual activity is reported. Obviously, both enzymes could not withstand higher H_2O_2 concentrations or the employed substrates could not provide sufficient protection against inactivation. Similarly, the same criteria were applied to screen LPMO activity (initial H_2O_2 concentration was 100 µM, data not shown) on the substrates listed in the main text, but only *Nc*AA9C showed activity on substrates besides xyloglucan, PASC and CNC.

(**G**) Off-pathway activity and futile H_2O_2 turnover of LPMOs (200 nM) determined in buffer using standard assay conditions. v_{enz} was determined by linear regression of the first 10 s of the reactions ($R^2 = 0.92$ or higher) and converted to turnover numbers. Importantly and expectedly, futile H_2O_2 turnover leads to fast self-inactivation, as shown by the rapid leveling-off of the H_2O_2 consumption time trace (see black time trace in **Figure 1C** of the main manuscript). To illustrate this the average number of H_2O_2 molecules (n) consumed in this futile reaction prior to enzyme inactivation is also shown (purple data points; secondary y-axis). The number of H_2O_2 molecules consumed per LPMO molecule was calculated by determining the difference between the starting concentration of H_2O_2 and the concentration detected after 200 s. At this time point, the time traces in the absence of substrate have levelled off fully, which indicates complete enzyme inactivation. On average, about 120 futile turnovers lead to full inactivation of AA9 LPMOs in the absence of substrate. For comparison, the collective data for productive reactions show that productive consumption of 100 µM H_2O_2 results in an average 500 (200 nM LPMO) to 2000 (50 nM LPMO) turnovers while maintaining between 23 and 97% of the initial enzyme activity. Clearly, and expectedly, the peroxidase off-pathway reaction, with its risk of causing enzyme inactivation, is drastically inhibited in the presence of substrate. Thus, the correction of the observed LPMO reaction rate, v_{obs}, with the rate of the off-pathway

reaction, $v_{off\text{-}path}$, which was applied in all of the experiments described, might lead to slight underestimation of the rate of the peroxygenase reaction, v_{enz}.

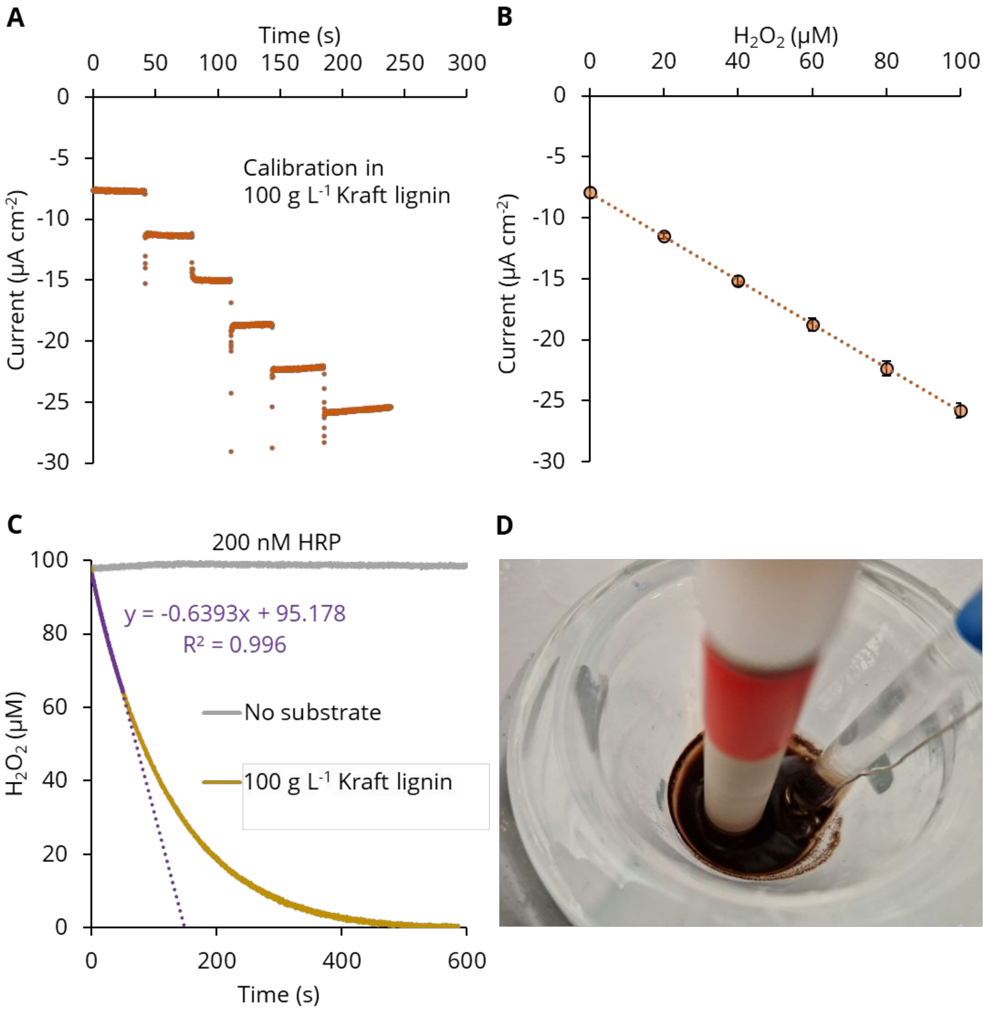

Figure S4. Revealing the activity of 200 nM horseradish peroxidase (HRP) acting on 100 g L^{-1} Kraft lignin. The experiment was conducted using standard assay conditions (30 mM sodium acetate containing 100 mM KCl, pH 6.0, 30°C, electrode rotation at 50 s^{-1}). Kraft lignin was suspended, and partially dissolved, by incubation overnight in the reaction buffer, on an orbital shaker at 30 °C and the pH was adjusted to 6.0 afterwards. (**A**) Exemplary calibration of the H_2O_2 sensor in the presence of 100 g L^{-1} Kraft lignin. Importantly, the stability of the signal and the fast response time of the H_2O_2 sensor are not affected by the presence of lignin components. The calibration function of this experiment is shown in (**B**), averaged over 3 independent measurements. (**C**) H_2O_2 consumption time trace for 200 nM HRP acting on 100 g L^{-1} Kraft lignin. The reaction in the absence of substrate (gray time trace), shows no consumption of H_2O_2 by HRP. The first 50 s of the H_2O_2 time trace in the presence of substrate were fit using linear regression (fit shown as purple dotted line) to determine the initial rate. Since HRP showed no off-pathway consumption of H_2O_2, the obtained enzymatic rate for H_2O_2 turnover was only corrected with the rate of substrate dependent consumption of H_2O_2 (0.05 s^{-1}). Three independent measurements showed that HRP converts Kraft lignin with a turnover number of 3.0 ± 0.1 s^{-1}. Although it is known that HRP can act on lignin components [72] to the best of our knowledge, this is the first time that HRP activity on a real lignin substrate is shown with a continuous method. (**D**) Image of the electrochemical cell and the H_2O_2 sensor rotating at 50 s^{-1} in the presence of 100 g L^{-1} Kraft lignin.

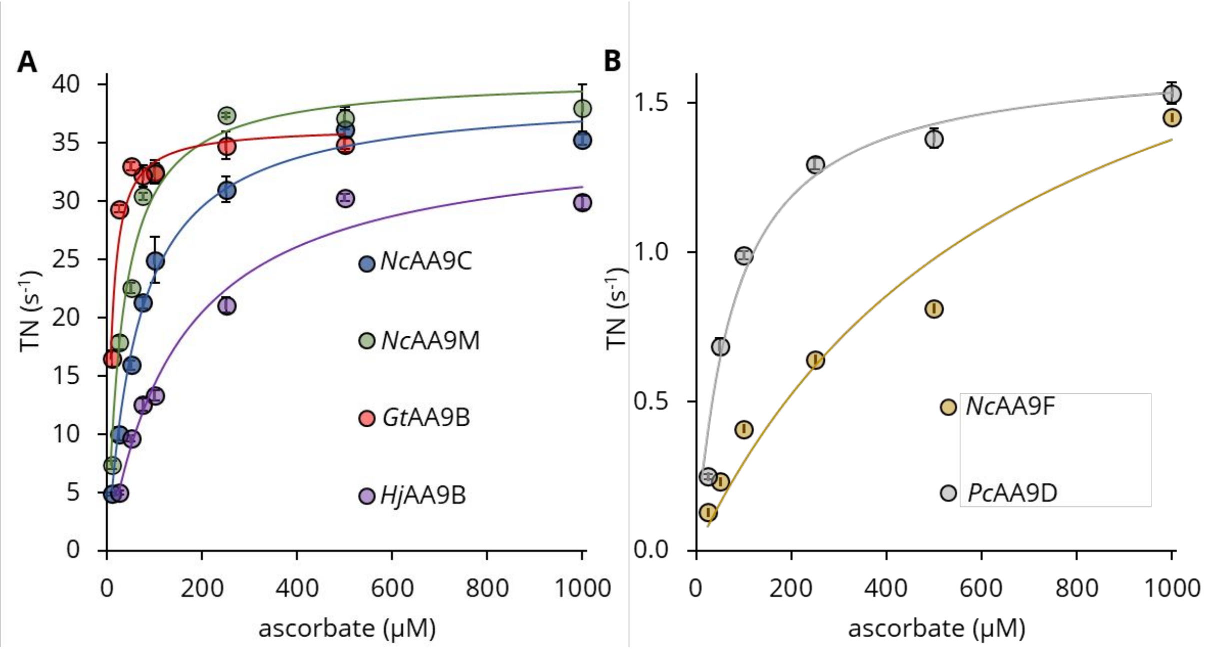

Figure S5. Ascorbate concentration-dependency of LPMO catalyzed reactions. Ascorbate concentration-dependent H_2O_2 turnover by LPMOs was studied using standard assay conditions and either xyloglucan (**A**) or PASC (**B**) as substrate. The ascorbate concentration necessary to reach saturating conditions depends on the respective enzyme. In accordance with the saturation curves depicted in this figure, we used 500 µM ascorbate as standard concentration in the measurements for all LPMOs except *Nc*AA9F and *Hj*AA9B (2 mM ascorbate). The ascorbate concentrations required to achieve half saturation for all LPMOs are shown in **Table S4**.

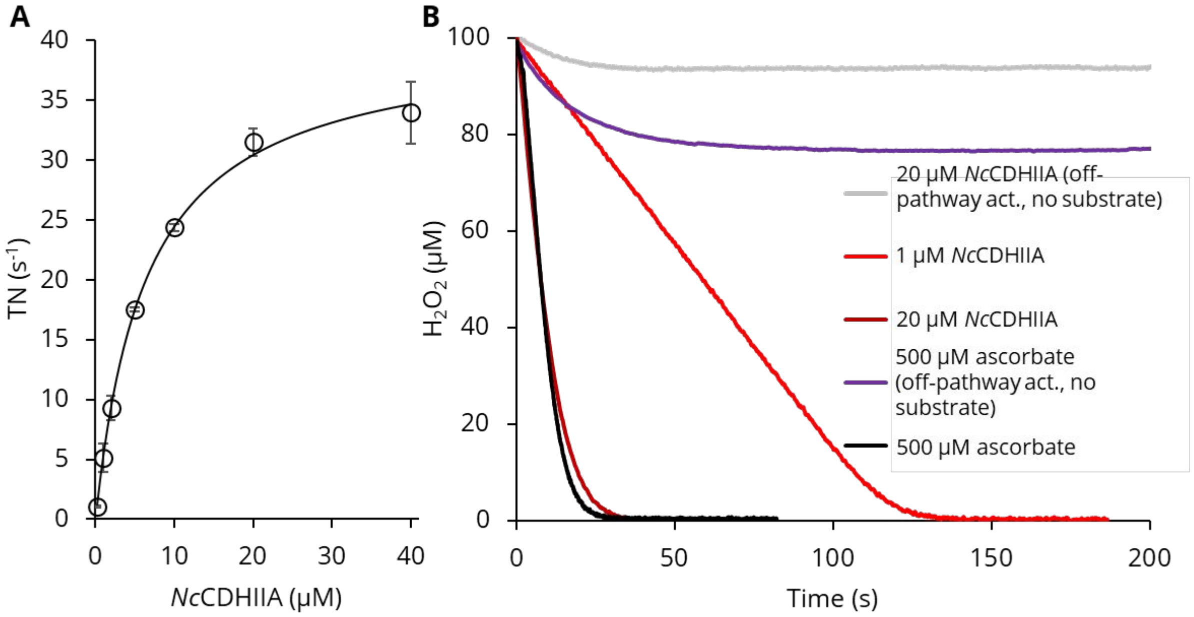

Figure S6. Comparison of *Nc*AA9C activity using either ascorbate or *Nc*CDHIIA for reduction. The dataset was recorded using standard assay conditions. (**A**) *Nc*CDHIIA was added in different concentrations to find the saturating CDH concentration for *Nc*AA9C acting on 4 g L^{-1} xyloglucan. The cytochrome domain of *Nc*CDHIIA was fully reduced by incubating the enzyme with 1 mM cellobiose for 30 s, before adding *Nc*AA9C to the reaction. The initial rates obtained for different *Nc*CDHIIA concentrations can be fitted to a hyperbolic function which results in a half saturating concentration of 6.5 ± 0.4 mM and a $TN_{max,app}$ of 40 ± 1 s^{-1}, which is in good agreement with the $TN_{max,app}$ determined for ascorbate (39 ± 1 s^{-1}, **Table S4**). (**B**) Comparison of LPMO peroxygenase and off-pathway activity in the presence of ascorbate or *Nc*CDHIIA. Due to the oxidase activity of 20 µM *Nc*CDHIIA, i.e. production of H_2O_2 resulting from oxidation of cellobiose, the off-pathway consumption of H_2O_2 by *Nc*AA9C in buffer appears "lower" compared to the reaction with ascorbate. However, catalysis of *Nc*AA9C acting on xyloglucan, fueled either by ascorbate or *Nc*CDHIIA used at saturating concentrations results in very similar H_2O_2 depletion curves, which shows that catalysis is independent of the used electron donor.

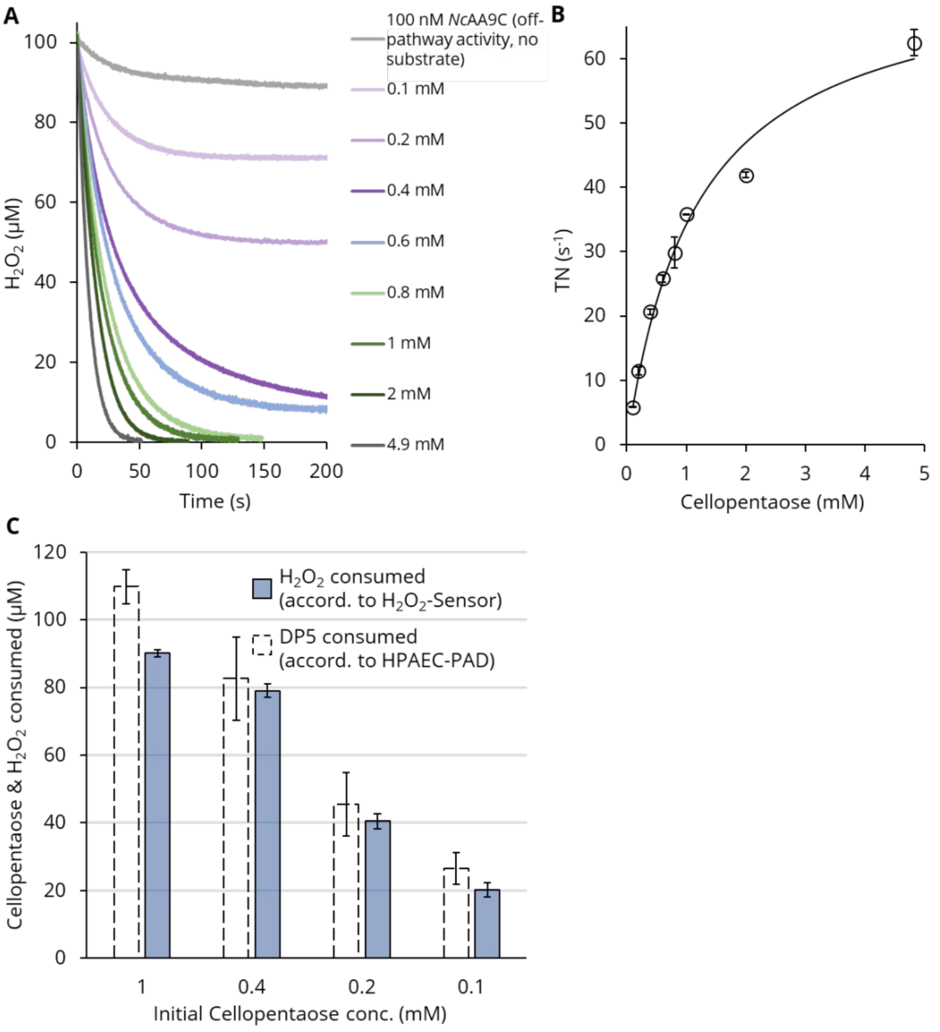

Figure S7. Cellopentaose conversion by *Nc*AA9C. (**A**) Time courses for H_2O_2 consumption by *Nc*AA9C in the presence of different cellopentaose concentrations. The experiments were done with 100 nM *Nc*AA9C and standard assay conditions (30 mM sodium acetate buffer, pH 6.0 and 100 mM KCl, 30 °C). (**B**) The dependence of initial catalytic rates of *Nc*AA9C acting on cellopentaose. The initial linear 15-20 s of the H_2O_2 time traces were fit with an R^2 of 0.98 or higher and used to calculate turnover numbers (corrected for off-pathway activity and v_{sb}). The data were fitted to a hyperbolic function ($R^2 = 0.99$), which can be used to extrapolate a $TN_{max,app}$ of 75 ± 5 s^{-1} and a $K_{M,app}$ of 1.2 ± 0.2 mM. (**C**) Verification of the cellopentaose:H_2O_2 stoichiometry by comparing the amount of cellopentaose consumed in the reactions of Panel A, as assessed by HPAEC-PAD (dashed bars), with H_2O_2 consumption, as depicted in panel A. For determination of product formation, reactions were stopped at different time points and the concentration of remaining cellopentaose was quantified. An exemplary chromatogram is displayed in **Figure S8**. The reactions with 0.1, 0.2 and 0.4 mM cellopentaose were stopped after 200 s by incubation at 90 °C for 10 min. The reaction with 1 mM cellopentaose was stopped after 130 s also by incubation at 90 °C for 10 min. The graph shows that the amount of consumed cellopentaose corresponds well with the amount of H_2O_2 consumed, confirming the 1:1 stochiometry. Importantly, the shown data for consumption of H_2O_2 and degradation of cellopentaose are from the same reaction and therefore allow direct comparison of co-substrate utilization and concomitant glycosidic bond cleavage.

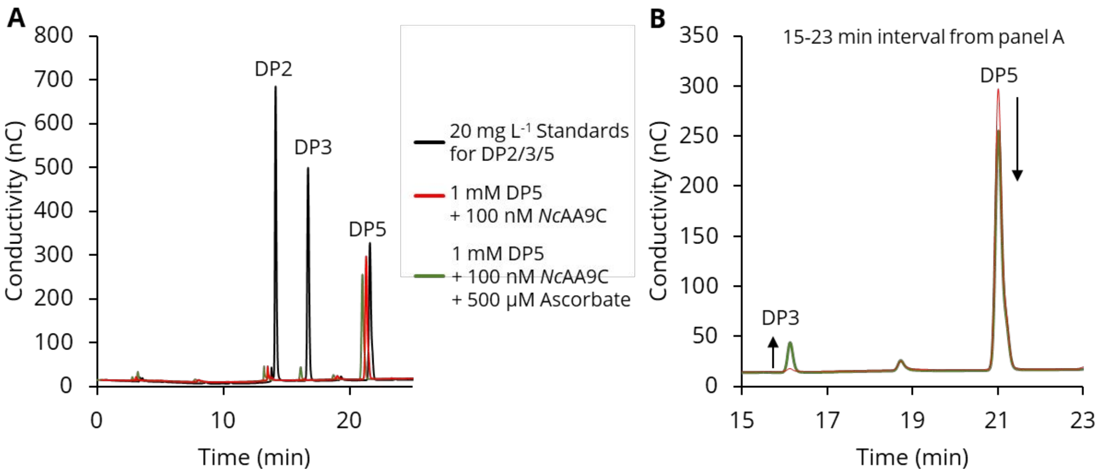

Figure S8. HPAEC-PAD chromatograms showing cello-oligosaccharide standards (DP2, 3 and 5) and *Nc*AA9C catalyzed cleavage of cellopentaose. (**A**) The chromatogram was obtained for a sample taken from a reaction featuring a 1 mM initial cellopentaose (DP5) concentration and 100 µM H_2O_2 (**Figure S7A**). The blank sample (red) was retrieved before addition of ascorbate. The next sample (green) was taken 130 s after starting the reaction by subsequent addition of 500 µM ascorbate and the LPMO. Both samples were diluted 50-fold prior to analysis. The 20 mg L^{-1} standards for cellobiose (DP2), cellotriose (DP3) and cellopentaose (DP5) were applied as a combined standard. The consumption of DP5 was quantified with standards in the range of 5 to 30 mg L^{-1}. The chromatograms are shown with a 0.3 min time-offset and show that when starting with 100 µM H_2O_2 and 1 mM substrate, about 10 % of the substrate (i.e., 100 µM) has been converted after 130 s, as expected (**Figure S7A**). (**B**) Section of the chromatograms shown in panel A (13–23 min interval) illustrate the decrease in the cellopentaose (DP5) concentration and the concomitant increase in cellotriose (DP3) concentration due to the LPMO catalyzed reaction.

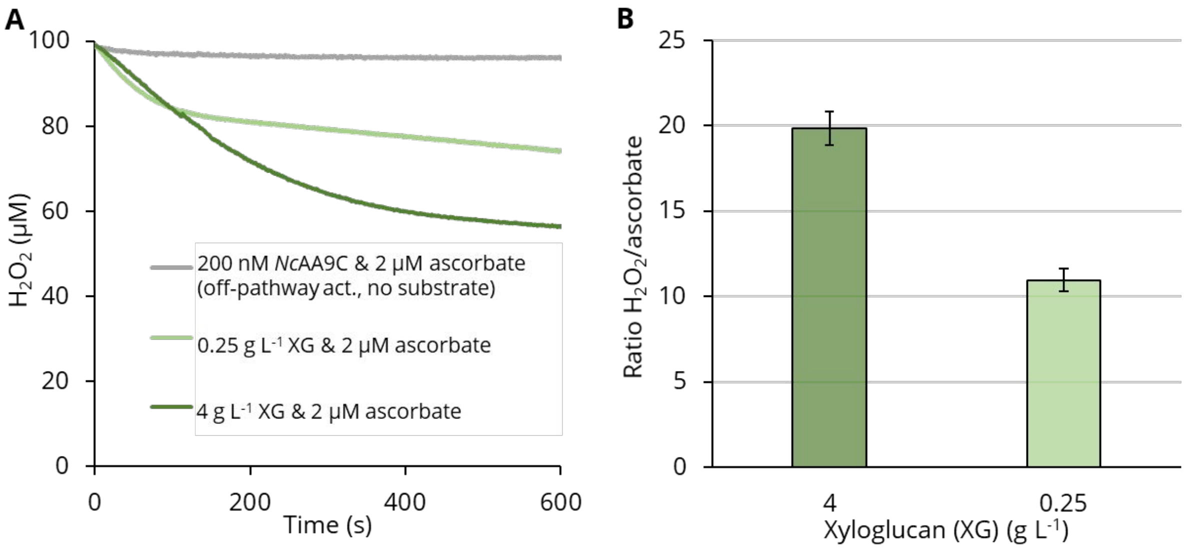

Figure S9. Ratio of consumed H_2O_2 molecules per consumed ascorbate molecule. The dataset was recorded using standard assay conditions (30 mM sodium acetate, pH 6.0, containing 100 mM KCl, 30 °C), with the following exceptions: (**A**) To determine the stoichiometry, the reaction was run with 200 nM *Nc*AA9C and a very low, 2 µM, ascorbate concentration. The low ascorbate concentration was chosen because it is not sufficient to enable full consumption of the supplied 100 µM H_2O_2. After H_2O_2 consumption was complete (600 s), the remaining H_2O_2 concentration was used to calculate the ratio of moles of consumed H_2O_2 to moles of consumed ascorbate, shown in panel (**B**), with correction for the off-pathway consumption of H_2O_2 (grey curve). The reaction of *Nc*AA9C acting on xyloglucan stopped once ascorbate was completely consumed, as indicated by the flattening of the time course. On the other hand, at saturating ascorbate concentrations (500 µM), the *Nc*AA9C catalyzed reaction consumes all of the added H_2O_2, and at a higher rate, as shown in **Figure 1D**. Panel (**B**) shows that the ratio of H_2O_2 molecules per consumed ascorbate decreases at lower substrate concentrations (0.25 g L^{-1}), showing that less efficient substrate binding leads to an increased futile H_2O_2 turnover, which again leads to higher reductant consumption. A comparable ratio of 18 cleavages per ascorbate molecule was previously reported for *Sm*AA10A acting on chitin [73].

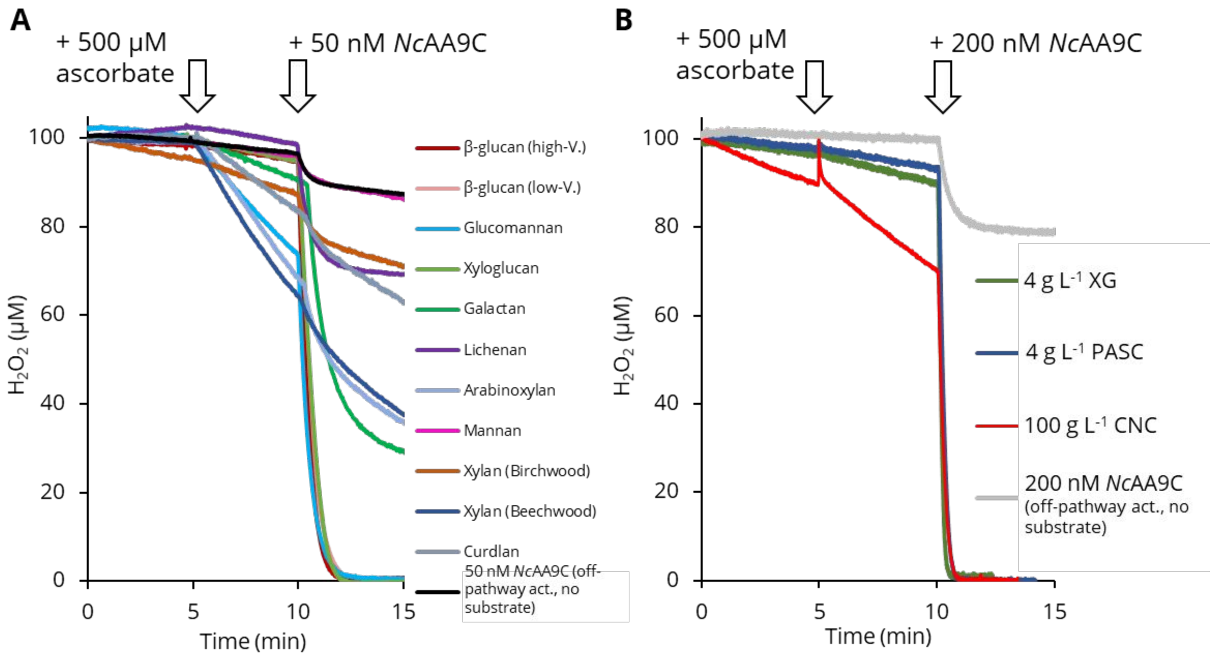

Figure S10. Determination of *Nc*AA9C activity on hemicellulose and cellulose substrates. The substrate screening reactions were conducted using standard assay conditions (hemicelluloses and PASC were used at 4 g L^{-1}, CNC at 100 g L^{-1}) and are divided into 3 parts, each consisting of a 5-min time period in which the change in the H_2O_2 concentration was recorded. First, the change in H_2O_2 concentration was studied in the presence of the potential substrate (0–5 min) to determine the influence of the substrate on the abiotic H_2O_2 consumption rate. In the second part, 500 µM ascorbate was added to the electrochemical cell (5–10 min), which in all cases increased H_2O_2 consumption; this phase provided the substrate background, v_{sb} (µM s^{-1}). In the third part, *Nc*AA9C was added to a final concentration of either (**A**) 50 nM or (**B**) 200 nM (10–15 min). The slopes obtained for the first two parts are summarized in **Table S7**, and the turnover numbers derived from the background-corrected reaction rates (v_{enz}) are summarized in **Table S6**.

Explained in more detail, the obtained rates for the LPMO reactions, v_{obs}, were converted to enzyme rates (v_{enz}) according to $v_{enz} = v_{obs} - v_{off\text{-}path} - v_{sb}$. The rate for the background reaction with the studied substrate and reductant (v_{sb}) was obtained from the second part of the progress curves, as outlined above. This is called substrate background correction and its magnitude depended on the studied system. The progress curves illustrate that the backgrounds for some hemicellulosic substrates (e.g. xylan from beechwood) were higher than for cellulosic substrates. The rate for the off-pathway activity of the LPMO ($v_{off\text{-}path}$) was derived from the third part of the reaction without substrate in the third part. The catalytic activities of all other LPMOs included in this study were assessed using the same experimental conditions, but were only active on xyloglucan, PASC and CNC. Their catalytic performance on these substrates is summarized in **Figure S3**.

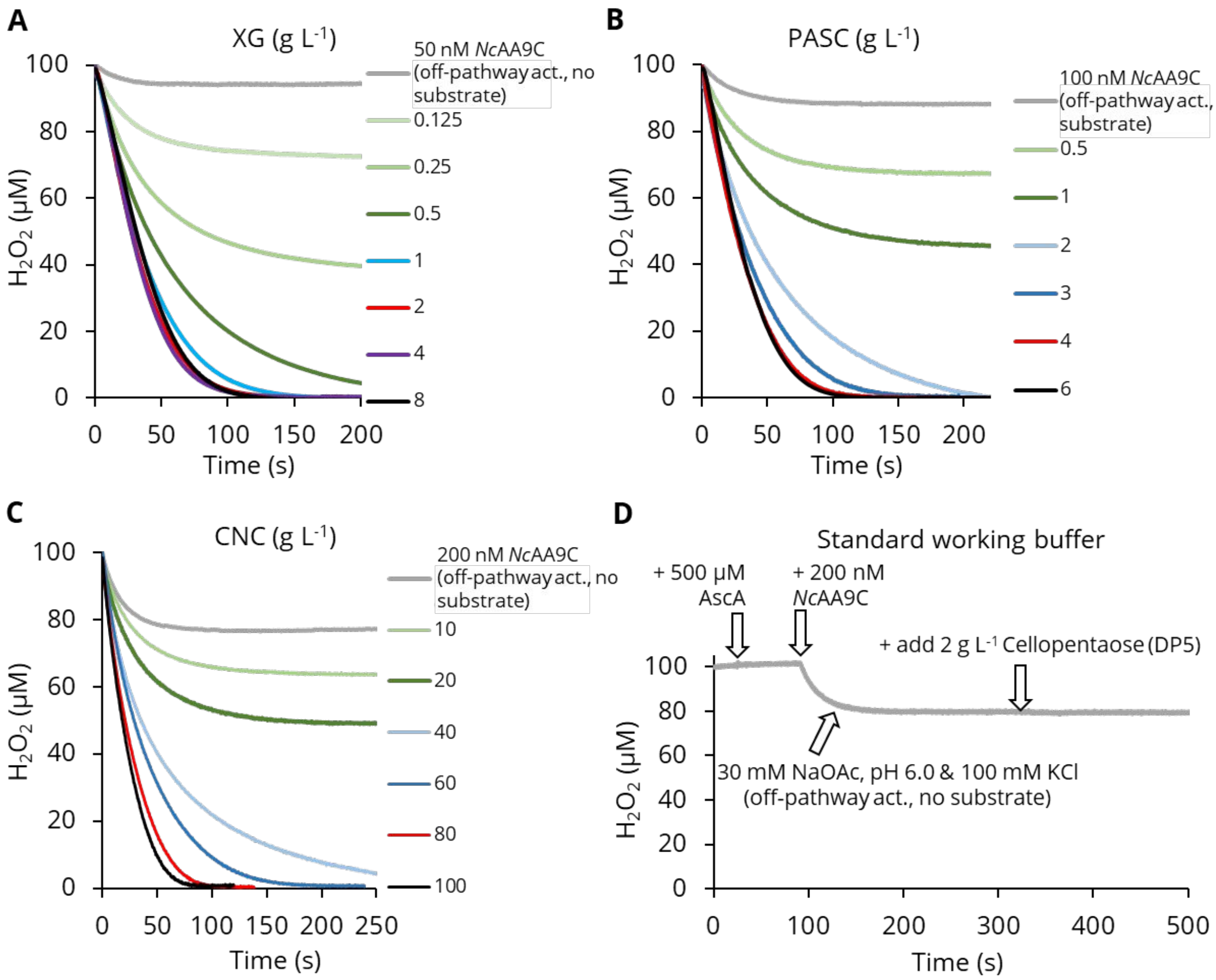

Figure S11. H_2O_2 consumption traces for *Nc*AA9C acting on different concentrations of (**A**) xyloglucan (50 nM *Nc*AA9C), (**B**) PASC (100 nM *Nc*AA9C) and (**C**) CNC (200 nM *Nc*AA9C). The reactions were performed under standard assay conditions (500 µM ascorbate, 30 mM sodium acetate buffer, pH 6.0 with 100 mM KCl at 30 °C). The time traces shown are the average of three independent measurements. The enzyme concentration was varied for the different substrates to obtain comparable reaction rates. The initial rates were obtained by linear fitting the 10 – 25 s part of the progress curves and were corrected for the substrate dependent background and the off-pathway activity of *Nc*AA9C as explained in detail in the Materials and methods and the legend of **Figure S10**. The derived turnover numbers are shown in the main text. All three panels illustrate the importance of high substrate concentrations to maintain *Nc*AA9C peroxygenase activity. Lower substrate concentrations result in enzyme inactivation, as indicated by the slowdown of the enzyme's H_2O_2 consumption rate before all available H_2O_2 is consumed. (**D**) Control experiment performed to validate that the slowdown and eventual cessation of H_2O_2 consumption is due to irreversible inactivation of *Nc*AA9C. The reaction was performed in standard assay buffer (30 mM sodium acetate, pH 6.0 containing 100 mM KCl) and started by the addition of 500 µM ascorbate followed by the addition of 200 nM *Nc*AA9C to monitor the off-pathway activity of LPMO in the absence of substrate. H_2O_2 consumption stopped some 50 s after addition of the LPMO. Subsequent addition of 2 g L^{-1} cellopentaose (DP5) directly into the measurement cell did not result in any change in the H_2O_2 concentration.

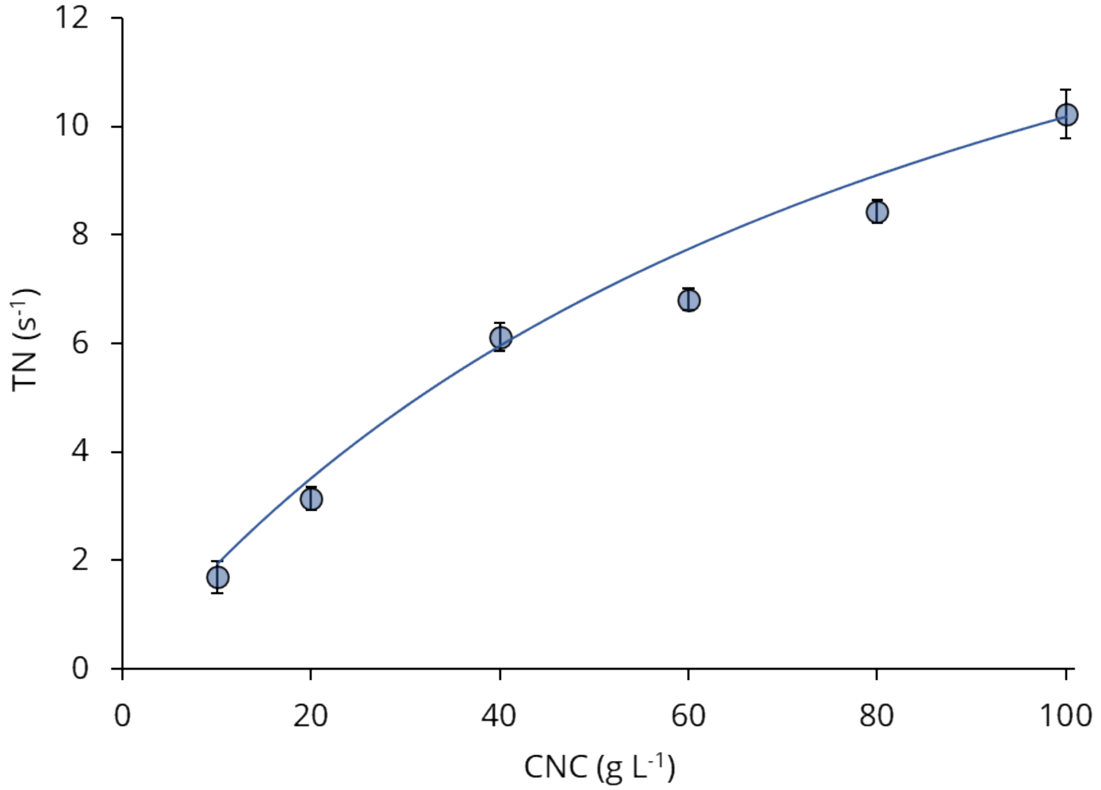

Figure S12. Saturation kinetics of *Nc*AA9C acting on different concentrations of CNC. The raw data are shown in **Figure S11C** and the $K_{M,app}$ obtained by fitting to a hyperbola function is given in **Table S8** and in the main text.

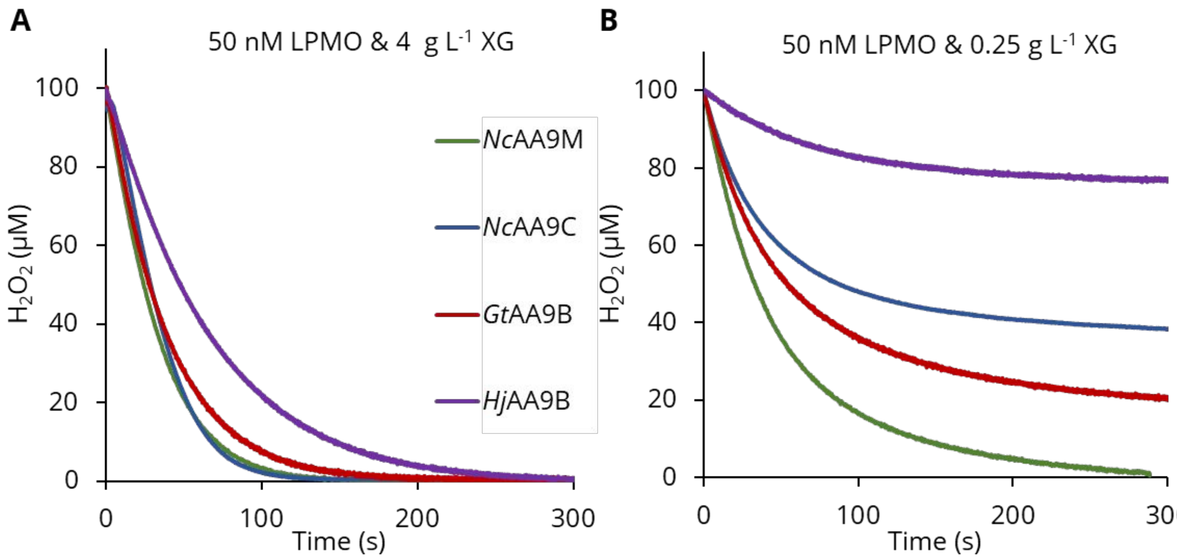

Figure S13. H_2O_2 time traces for four xyloglucan-active LPMOs, *Nc*AA9M, *Nc*AA9C, *Gt*AA9B, *Hj*AA9B. The reactions were performed under standard assay conditions containing 50 nM LPMO. The time traces shown are an average of three independent measurements. (**A**) Reactions with 4 g L^{-1} xyloglucan; similar rates were obtained for *Nc*AA9M, *Nc*AA9C and *Gt*AA9B acting on xyloglucan (TN 30–35 s^{-1}). The calculated turnover numbers and the corresponding residual activities are shown in **Figure S3**. (**B**) Reactions with 0.25 g L^{-1} xyloglucan; these reactions reveal difference between the LPMOs. *Nc*AA9M, and to some extent *Gt*AA9B, are still able to reach quite high turnover numbers despite the low substrate concentration. This could be explained by their higher tolerance to xylose substitutions on the xyloglucan backbone, which has been previously documented for *Nc*AA9M [57] and a homologue of *Gt*AA9B [56] and which would increase the effective substrate concentration.

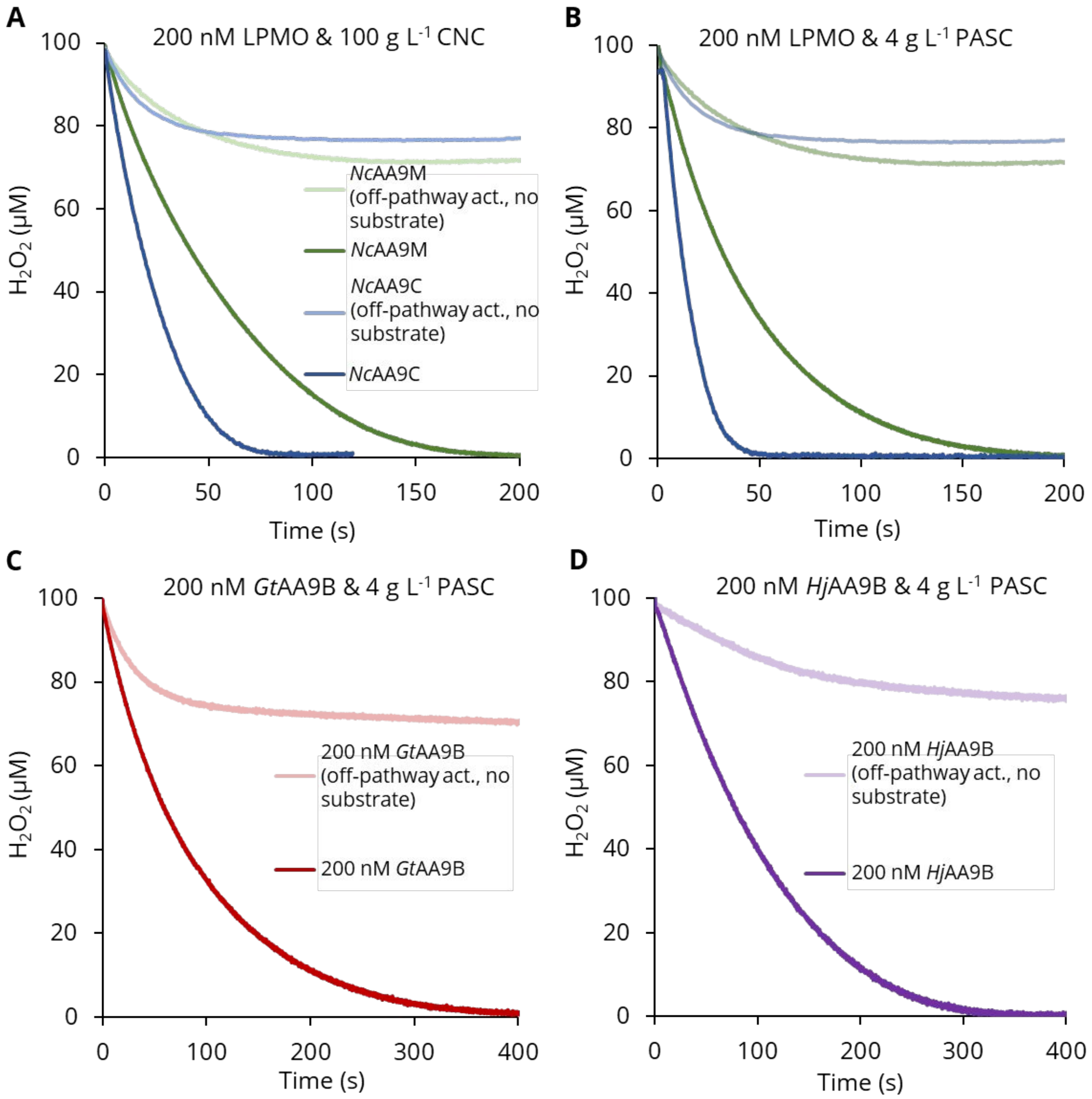

Figure S14. H_2O_2 time traces for C4-oxidizing LPMOs acting on cellulose. Experiments were performed under standard assay conditions with PASC or CNC as substrates (200 nM LPMO, 500 µM ascorbate; except for *Hj*AA9B where 2 mM ascorbate was used to achieve full saturation). (**A**) *Nc*AA9C and *Nc*AA9M acting on 100 g L^{-1} CNC, (**B**) *Nc*AA9C and *Nc*AA9M acting on 4 g L^{-1} PASC, (**C**) *Gt*AA9B acting on 4 g L^{-1} PASC and (**D**) *Hj*AA9B acting on 4 g L^{-1} PASC. The derived turnover numbers and the residual activities are summarized in **Figure S3**.

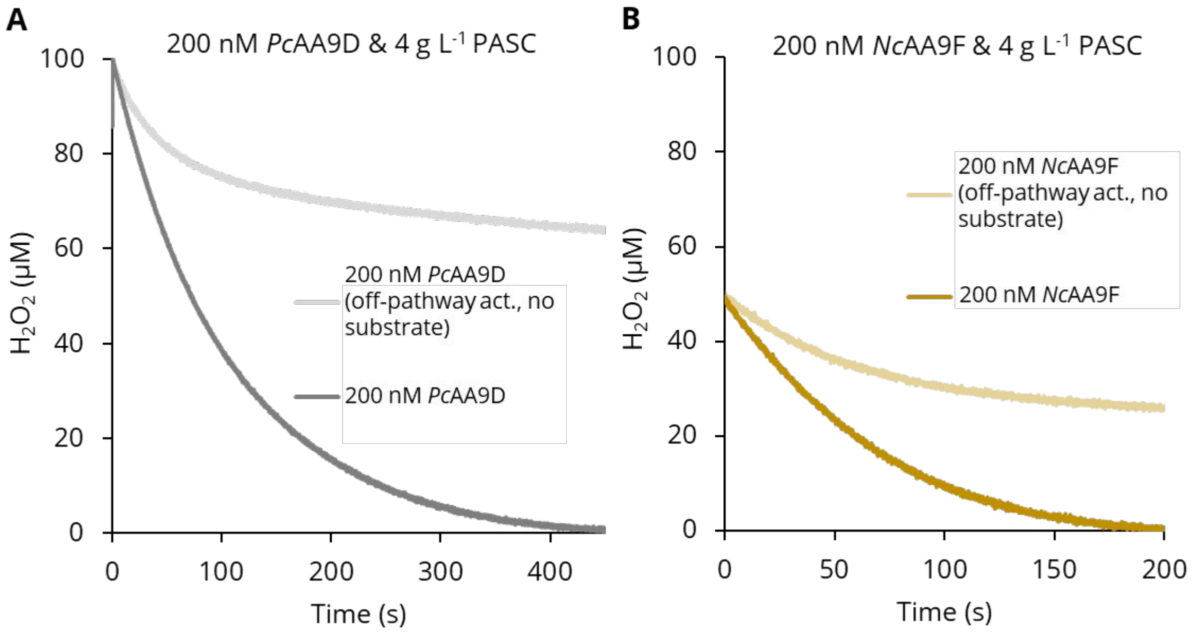

Figure S15. H_2O_2 time traces for C1-oxidizing LPMOs acting on cellulose. Experiments were performed under standard assay conditions with PASC as substrate (200 nM LPMO, 500 µM ascorbate; except for *Nc*AA9F where 2 mM ascorbate was used to achieve full saturation and where the starting H_2O_2 concentration was only 50 mM, because of rapid inactivation at 100 µM H_2O_2). (**A**) *Pc*AA9D acting on 4 g L^{-1} PASC, (**B**) *Nc*AA9F acting on 4 g L^{-1} PASC. The derived turnover numbers and residual activities are summarized in **Figure S3**.

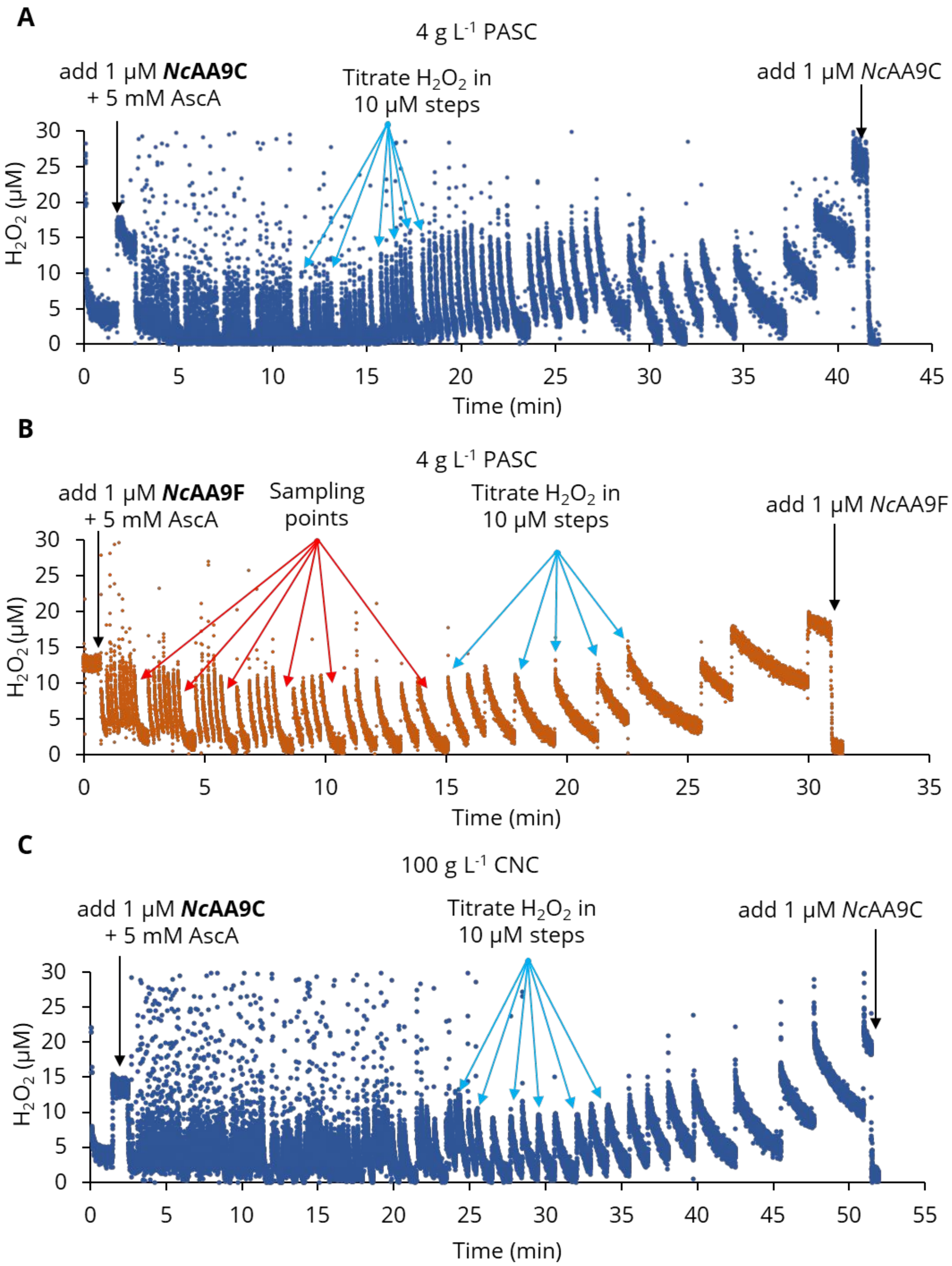

Figure S16. Raw data for the determination of total turnover numbers for LPMOs acting on PASC or CNC. All experiments were conducted using standard assay conditions, however the LPMO concentration was 1 µM of LPMO and we used a high ascorbate concentration (5 mM) to ensure that the reductant concentration was not limiting. The ascorbate stock was prepared in 30 mM sodium acetate buffer and adjusted to pH 6.0. The co-substrate, H_2O_2, was added in 10 µM steps. Each addition was followed by waiting until the H_2O_2 was completely consumed, after which another 10 µM of H_2O_2 was added, and this was repeated until only a fraction of the initial LPMO activity remained. The H_2O_2 titration steps are exemplarily shown

by a bundle of blue arrows. Due to the gradual decrease in LPMO activity, the time passing between the injections of 10 µM H_2O_2 increases over time, as is clearly visible in the right half of the Figures. The H_2O_2 titration steps are summed up to the total turnover number (TTN) summarized in **Table S9**. The obtained summed up H_2O_2 turnovers were corrected for the substrate dependent background consumption of H_2O_2 over the course of the experiment. The observed turnovers include productive turnovers of the peroxygenase activity, as well as off-pathway consumption of H_2O_2 eventually leading to complete loss in activity. The initial rate obtained after approximately every 5th titration step was used to estimate the amount of residual activity left, by comparing to the initial rate obtained in the first H_2O_2 titration step (=100 %). These residual activities are plotted as a function of H_2O_2 turnover (n) in **Figure 3** of the main text. When the residual enzyme activity was close to zero, 1 µM fresh LPMO was added to the reactions, which, in all cases, led to immediate depletion of remaining H_2O_2, showing that the decrease in LPMO activity was not caused by substrate or reductant depletion. (**A**) *Nc*AA9C-catalyzed turnover of PASC. (**B**) *Nc*AA9F-catalyzed turnover of PASC. During this experiment, 500 µL aliquots were collected at ten different time points to determine the total amount of C1- oxidized products formed using HPAEC-PAD analysis. The collected samples were heat inactivated at 90 °C for 10 min. The respective sampling points are indicated by a bundle of red arrows. For clarity, sampling time points after 15 min are not indicated. Progress curves based on H_2O_2 consumption data and the total amount of off-line detected C1-oxidized products are shown in **Figure 3A** of the main text and confirm that H_2O_2 consumption correlates with substrate oxidation**.** Exemplary chromatograms for offline detection of C1-oxidized products of this experiment are shown in **Figure S17**. (**C**) *Nc*AA9C-catalyzed turnover of CNC.

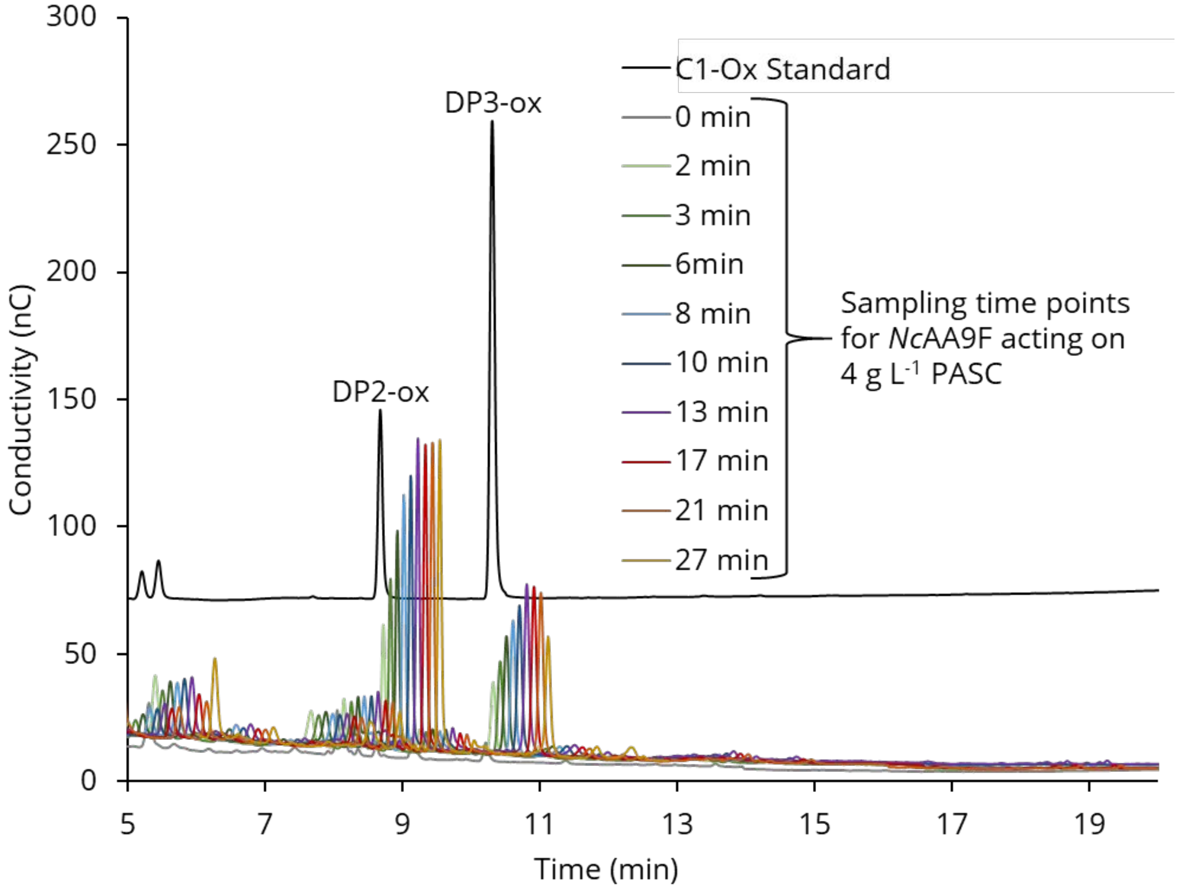

Figure S17. HPAEC-PAD analysis of C1-oxidized products generated by *Nc*AA9F acting on 4 g L^{-1} PASC. The displayed chromatograms (plotted with a 0.1 min offset) show the total amount of oxidized sites generated by *Nc*AA9F. Polymeric and oligomeric material in the reaction mixtures was hydrolyzed in a single reaction by incubating the reaction suspension for 48 h at 40 °C and 1000 rpm with 5 µM *Thermobifida fusca* Cel6A to hydrolyze soluble products and remaining PASC to native and oxidized dimers (DP2) and trimers (DP3). To obtain time points, the *Nc*AA9F reactions were sampled after which enzyme activity was stopped by heat inactivation (10 min at 90 °C). H_2O_2 titration and consumption data for this experiment are shown in **Figure S16B**. The H_2O_2 titration data and the levels of C1-oxidized products are compared in **Figure 3A** of the main text.

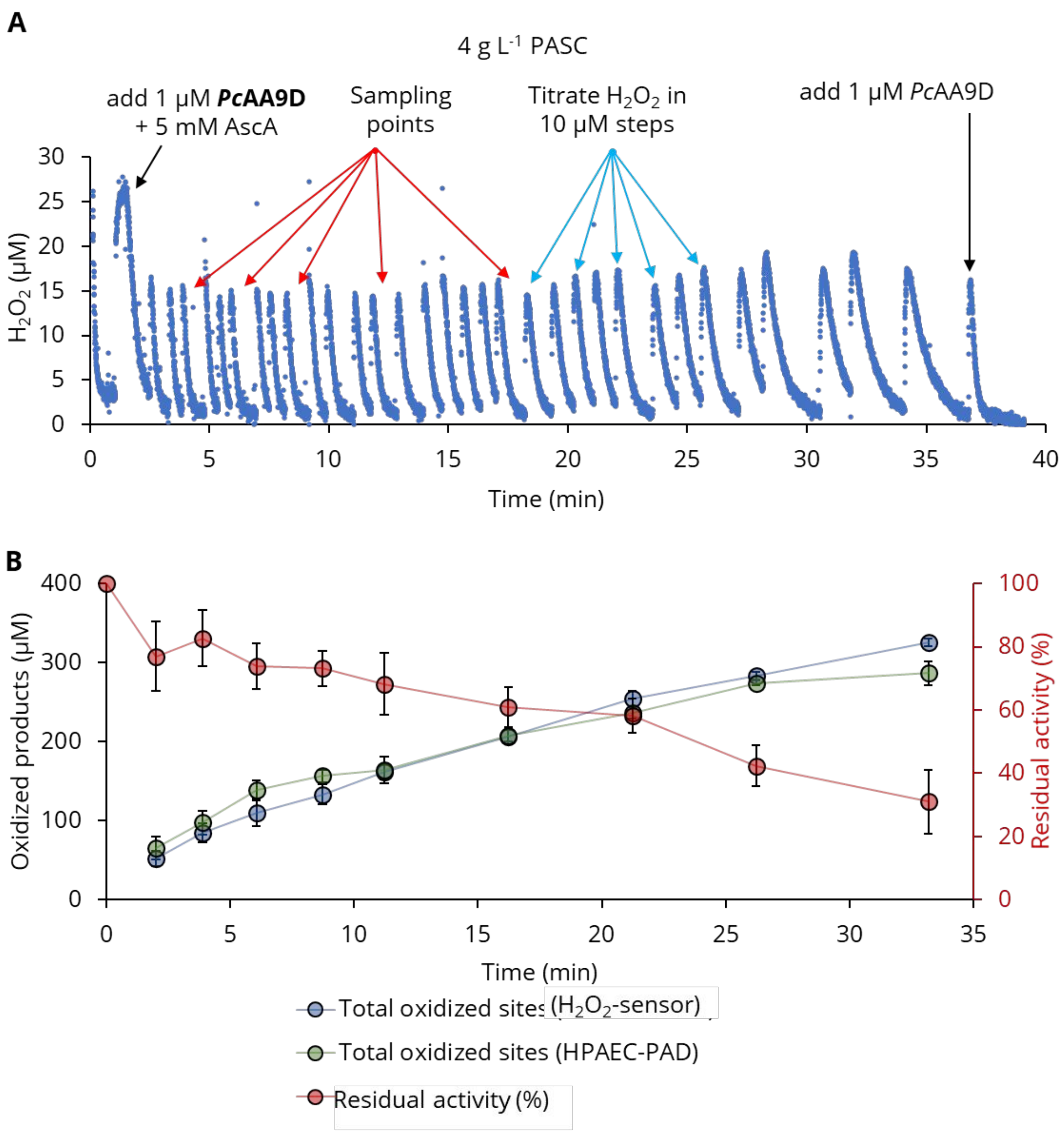

Figure S18. Determination of the total turnover number for *Pc*AA9D acting on 4 g L^{-1} PASC and the stoichiometry of oxidized products versus H_2O_2 consumption. (A) Raw H_2O_2 titration data obtained for 1µM *Pc*AA9D acting on 4 g L^{-1} PASC. The experiment was conducted as described in the legend of **Figure S16**, i.e., using standard assay conditions, but with 5mM ascorbate. The H_2O_2 was titrated in 10 µM steps. During this titration experiment, 500 µL aliquots were collected, heated at 90 °C for 10 min to inactivate the enzyme, and subsequently analyzed for the amount of oxidized products using a cellulase treatment followed by HPAEC-PAD, as described in the legend of **Figure S16**. Exemplary sampling points are indicated by a bundle of red arrows. (**B**) Correlation of H_2O_2 consumption according to the titration experiment shown in (**A**) and the amount of generated C1-oxidized products (sum of DP2-ox and DP3-ox) formed according to HPAEC-PAD analysis. Panel **(B)** also shows residual activities that were determined regularly, as described in the legend of **Figure S16** and illustrated by the exemplary bundle of red arrows shown in panel **A**. The residual activity as a function of H_2O_2 turnover and the TTN coming out of this experiment are shown in **Figure 3**

of the main text and **Table S9**. See the legend of **Figure S16** for further details and considerations.

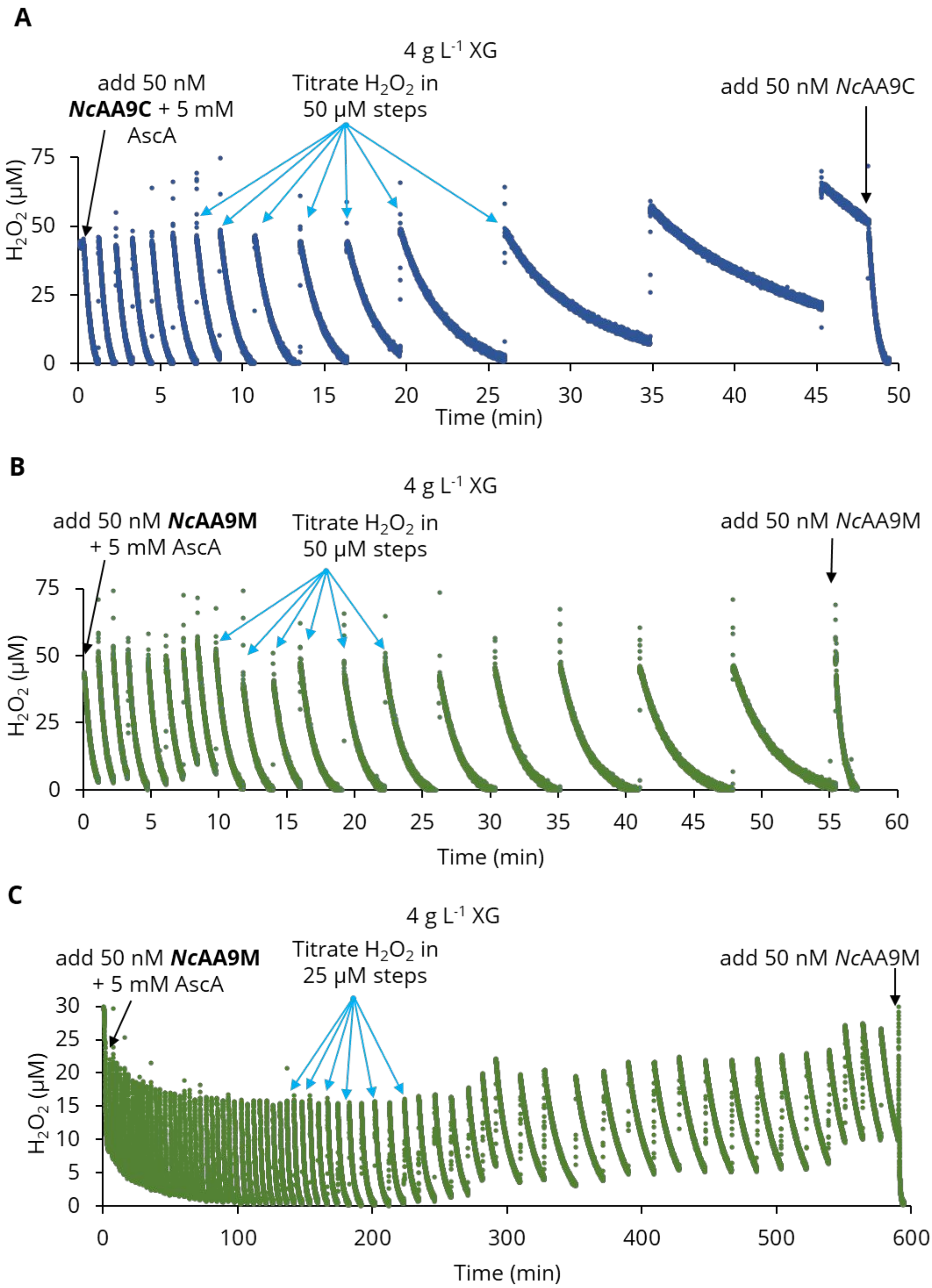

Figure S19. Raw data for the determination of total turnover numbers for LPMOs acting on xyloglucan. The experiments were conducted under standard conditions using 50 nM *Nc*AA9C or *Nc*AA9M and 4 g L^{-1} xyloglucan as substrate. Five mM ascorbate was used to ensure the presence of sufficient reducing agent throughout the measurement. The ascorbate stock solution was prepared in 30 mM sodium acetate and adjusted to pH 6.0. H_2O_2 was titrated in with 50 µM (Panels **A** and **B**) or 25 µM (Panel **C**) additions. The individual titration steps were summed up and corrected with the substrate dependent consumption of H_2O_2 over the course of the experiment to determine the total turnover numbers, which are summarized in

Table S9. Residual activities were determined as described in the legend of **Figure S16** and are reported in **Figure 3** of the main manuscript. Turnover of H_2O_2 includes productive turnovers of the peroxygenase activity as well as non-productive futile turnovers eventually leading to a loss of activity.

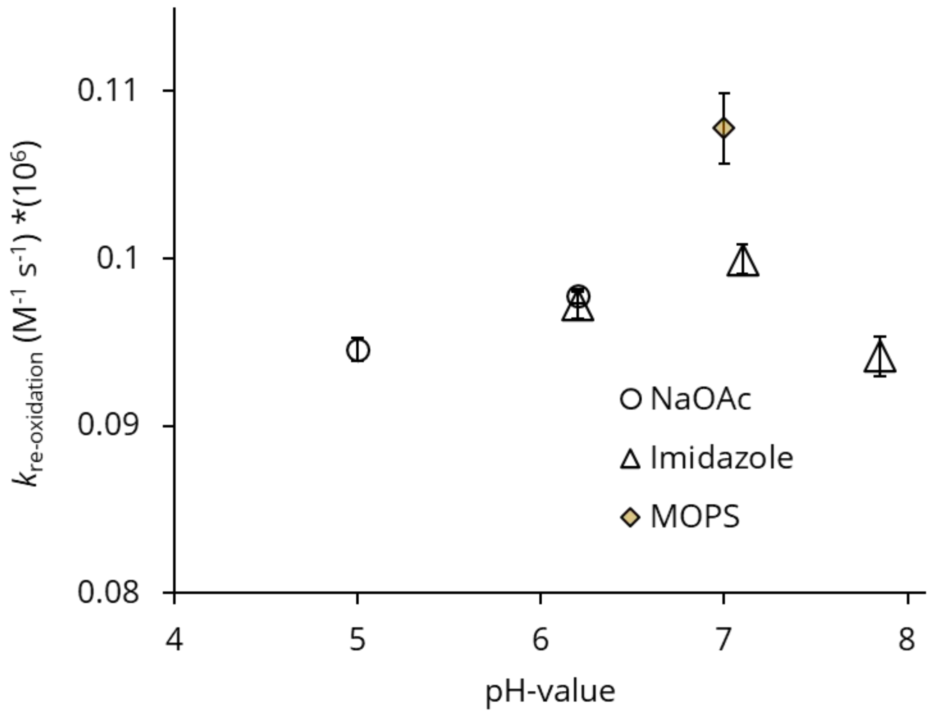

Figure S20: The pH-dependency of the second-order reoxidation rate of *Nc*AA9C by H_2O_2 in absence of substrate. Reoxidation rates were recorded in 50 mM sodium acetate (pH 5.0 & 6.0), imidazole-HCl (6.0 – 8.0), or MOPS-HCl (7.0) buffer at 30 °C. The second-order rate for re-oxidation of *Nc*AA9C-Cu(I) by H_2O_2 shows no pH-dependency, fluctuating at about 100,000 M^{-1} s^{-1} between pH 5.0 and 8.0. While this may seem in contrast with the pH-activity profiles described in **Figure 4** of the main text, the low pH-dependency of the rate of reoxidation by H_2O_2 cannot be extrapolated to what happens during productive catalysis, since binding of the substrate has a massive effect on H_2O_2 turnover, increasing it by as much as 50-fold [28].

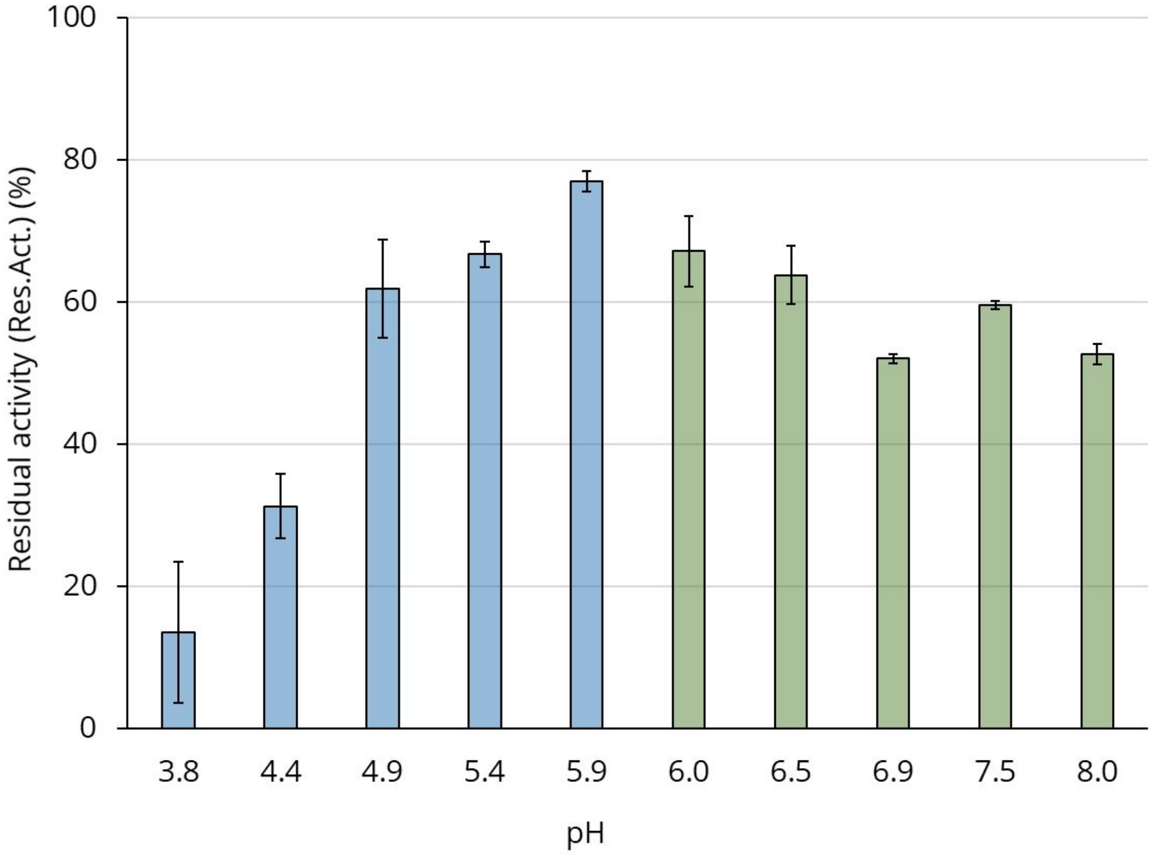

Figure S21. Residual activities obtained from measurements of the pH-activity profile shown in Figure 4A for *Nc*AA9C acting on 4 g L^{-1} xyloglucan. Two different buffers were used to cover the pH range: 30 mM sodium acetate buffer from pH 4.0 to 5.9 (blue bars) and 30 mM imidazole chloride buffer from pH 6.0 to 8.0 (green bars). All buffers contained 100 mM KCl. The pH-activity profile was obtained by increasing the ascorbate concentration at lower pH due to the reduced ability of ascorbate to reduce the LPMO, as explained in the main text. The ascorbate concentration at each pH was 10-fold the half-saturating concentration, which was estimated from the determined half-saturating concentrations at pH 4.0, 5.0, 6.0 and 7.0 (**Figure 4C**). The residual activities were determined for each pH and apply to the residual activity after one measurement cycle starting with 100 µM H_2O_2, as outlined in the legend of **Figure 1** of the main manuscript and the Experimental Procedures. In short, after the first reaction cycle, the same amount of H_2O_2 was added again and the time trace was recorded to determine the residual initial rate. The displayed pH-values are the true values measured at the end of the measurement, i.e. after the second cycle was completed.

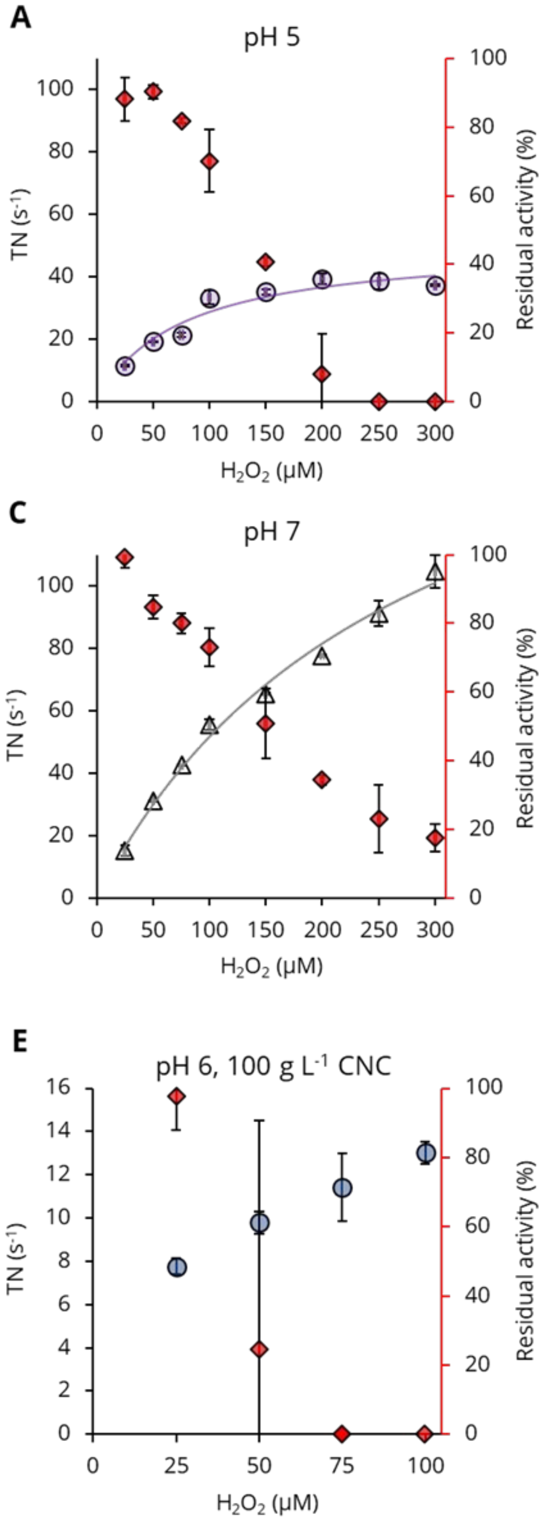

Figure S22. H_2O_2, pH, substrate and inactivation-dependent kinetics for 50 nM *Nc*AA9C acting on 4 g L^{-1} xyloglucan or 100 g L^{-1} CNC. The TN values shown in panels **A-D** also appear in **Figure 4D** of the main manuscript. The data set was recorded using standard assay conditions, but the initial H_2O_2 concentration was varied between 25 and 300 µM. The pH-range was covered by using a 30 mM sodium acetate buffer for pH 5.0 and 6.0, while an imidazole chloride buffer was used for pH 7.0 and pH 8.0. The ascorbate stock solution was

pre-buffered to the respective pH of the experiment. In order to only vary one parameter at a time, the ascorbate concentration was kept constant at 3 mM throughout all experiments. This concentration corresponds to 10-times the half saturating concentration for ascorbate at pH 5 (at this pH, the half saturating concentration is at its highest when considering the pH 5 – 8 range). This ascorbate concentration should ensure full reduction of the LPMO at all tested pH-values. For each H_2O_2 concentration, the residual activity was determined after the initial amount of H_2O_2 had been consumed. To do so, 25-300 µM H_2O_2 was added for a second time after reaching the baseline (i.e. 0 µM H_2O_2) and the second cycle of H_2O_2 depletion was followed until the baseline was reached again (where the latter was not always achieved due to enzyme inactivation). Residual activities were calculated as the ratio of the initial reaction rates derived from the second and first reaction cycles of the experiments, as described in Experimental Procedures and the legend of **Figure 1** of the main text. The first 15 s of the 1st and the 2nd cycle of all measurements were used to determine the initial rate. The obtained kinetic constants are summarized in **Table S10**.

(**A** – **D**) Activity of *Nc*AA9C with increasing H_2O_2 concentrations at pH 5.0, 6.0, 7.0 and 8.0. While the used H_2O_2 concentrations were almost saturating at pH 5.0 and 6.0, saturation was less pronounced at pH 7.0 and 8.0. Since enzyme inactivation, expectedly, becomes more prominent at higher H_2O_2 concentrations, and despite the fact that the reported rates are initial rates derived from reasonably straight initial parts of the H_2O_2 depletion curve, it is not clear whether the observed saturation is due to a true kinetic saturation (e.g. in the form of a half saturating concentration or $K_{M,app.}$) or due to overlaying effects related to self-inactivation.

(**E**) To find out if the observed apparent saturation depends on the substrate, we repeated the experiment at pH 6.0 using 100 g L^{-1} CNC as the substrate for 50 nM *Nc*AA9C. The H_2O_2 concentration was varied between 25 and 100 µM. The reactions with 75 and 100 µM H_2O_2 led to full enzyme inactivation after one reaction cycle and for this reason higher H_2O_2 concentrations were not tested. The dataset shows a small increase in initial reaction rate at increasing H_2O_2 concentration, with no signs of saturation and early onset of enzyme inactivation. This different behavior with cellulose, compared to xyloglucan, underpins the impact of the substrate on LPMO catalysis and stability. Looking at panels A-E, it is worth noting that the expected low micromolar concentrations of H_2O_2 in natural ecosystems may lead to stable LPMO reactions with appreciable rate, but are far from saturating.

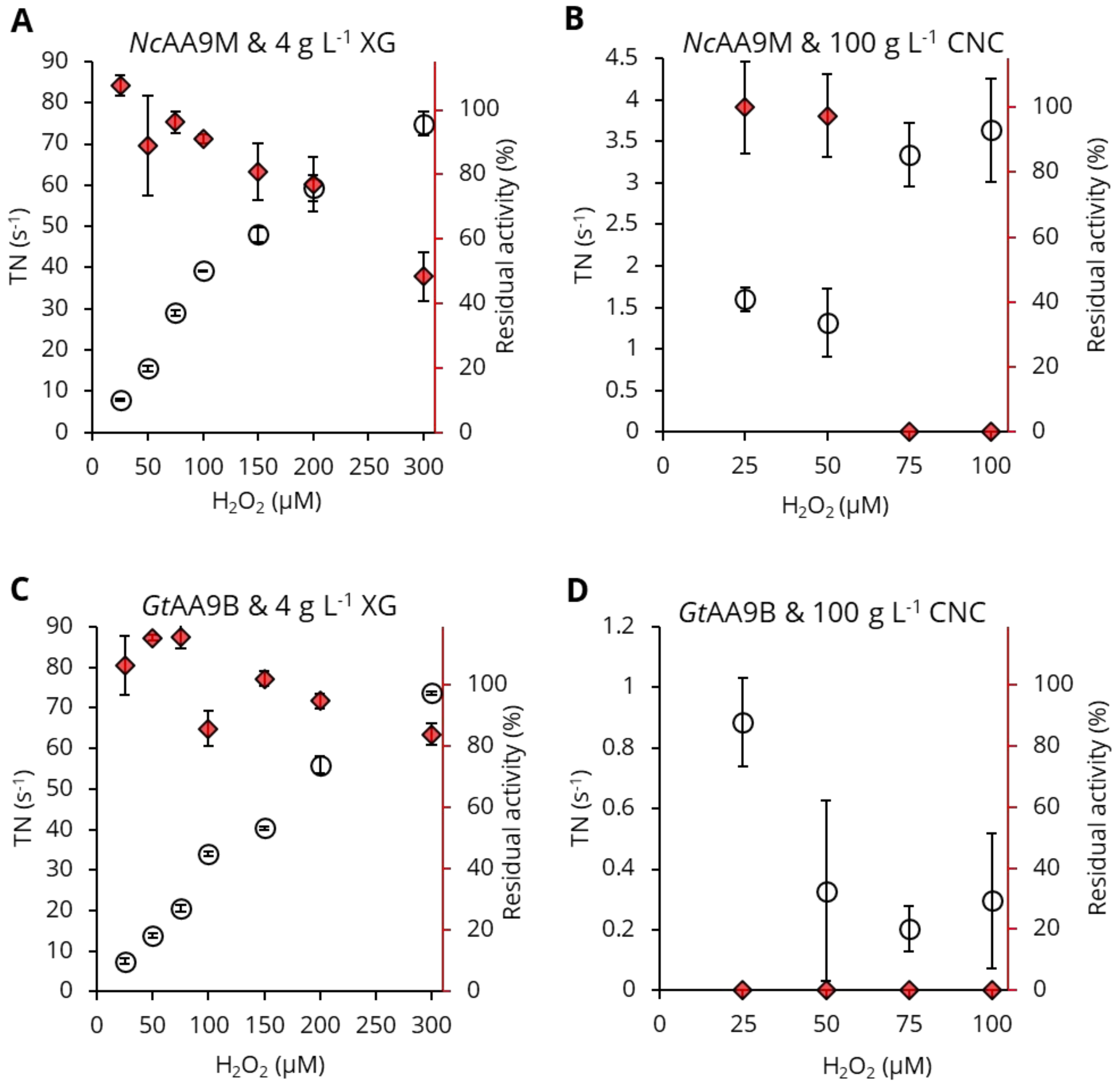

Figure S23. H_2O_2, substrate and inactivation-dependent kinetics of 50 nM *Nc*AA9M and *Gt*AA9B acting on 4 g L^{-1} xyloglucan or 100 g L^{-1} CNC. The data set was recorded using standard assay conditions (30 mM sodium acetate containing 100 mM KCl, pH 6.0, 500 µM ascorbate, 30 °C), but the initial H_2O_2 concentration was varied between 25 and 300 µM. Residual activities after one reaction cycle were determined as described in the legend of **Figure S22**.

(**A-B**) H_2O_2 and substrate (xyloglucan vs. CNC) dependent kinetics of *Nc*AA9M. Turnover numbers for *Nc*AA9M acting on xyloglucan increase almost linearly between 25 and 300 µM H_2O_2 and residual activities only decrease to half of the initial activity at the highest H_2O_2 concentration. No apparent saturation could be observed; therefore, no apparent catalytic rate constants were derived from this dataset. For CNC the situation is very different, i.e. lower rates and more inactivation, much alike what was observed for *Nc*AA9C (**Figure S22B, E**)

(**C-D**) The same experiment was conducted using *Gt*AA9B and for this LPMO the difference between xyloglucan and CNC was even bigger. Activity on xyloglucan increased almost linearly between 25 and 300 µM H_2O_2, with remarkably little enzyme inactivation, whereas the apparent activity on CNC was low at all tested H_2O_2 concentrations, with rapid enzyme

inactivation. These results for *Gt*AA9B underpin the huge impact of the substrate on LPMO performance. While a good soluble, rapidly diffusing substrate like, in this case, xyloglucan leads to fast and stable reactions at almost any H_2O_2 concentration, enzyme performance in the presence of CNC is very different.

Supplementary tables

Table S1. Summary of published traits of the LPMOs used in this study.

LPMO	Other names	Organism	Uniprot ID	PDB code	Substrates[1]	Regioselectivity	References
***Nc*AA9C (+CBM1)**	PMO-02916<br>GH61-3<br>NCU02916<br>*Nc*LPMO9C	*Neurospora crassa* OR74A	Q7SHI8	4D7U<br>4D7V	PASC, RAC, MCC, CMC, cello-oligosaccharides (DP4-6), steam-exploded spruce, xyloglucan (Tamarind), un-substituted β-D-glucans: glucomannan (Konjac), mixed linkage (β-1-4/1-3) β-D-glucan (barley), lichenan (Islandic moss)	C4	9,33,34,54,65,74–76
***Nc*AA9M**	PMO-3<br>GH61-13<br>NcPMO-3<br>NCU07898<br>*Nc*LPMO9M	*Neurospora crassa* OR74A	Q7SA19	4EIS	RAC, CMC, PASC, xyloglucan (Tamarind)	C1/C4	1,57,65,77
***Nc*AA9F**	PMO-03328<br>LPMO -03328<br>GH61-6<br>NCU03328<br>*Nc*LPMO9F	*Neurospora crassa* OR74A	Q8WZQ2	4QI8	MCC, PASC, xylan (Beechwood, only in combination with PASC)	C1	10,34,78
***Gt*AA9B**	Cel61G<br>*Gt*GH61<br>*Gt*LPMO9B	*Gloeophyllum trabeum* KUC	F8T947	n.a.	PASC, xyloglucan (Tamarind)	C1/C4	56,59
***Hj*AA9B**	*Hj*LPMO9B<br>*Hj*GH61B<br>Cel61B	*Hypocrea jecorina QM6a*	Q7Z9M7	2VTC	not determined		64
***Pc*AA9D**	GH61D<br>*Pc*GH61D<br>*Pc*LPMO9D	*Phanerochaete chrysosporium K-3*	H1AE14	4B5Q	PASC, MCC	C1	32,66,79,80

Abbreviations: DP, degree of polymerization; PASC, phosphoric acid swollen cellulose; RAC, regenerated amorphous cellulose; CMC, carboxymethyl cellulose; MCC, microcrystalline cellulose; n.a., not available. [1]Note that substrates that are not listed may not have been tested.

Table S2. H_2O_2 sensor performance in buffer and different substrate-containing solutions or suspensions. The data shown were obtained from experiments using *Nc*AA9C acting under standard assay conditions on different xyloglucan, PASC or CNC concentrations in 30 mM sodium acetate buffer pH 6.0 containing 100 mM KCl. The calibration data was obtained from experiments with a final H_2O_2 concentration of 100 µM and for each substrate a newly prepared electrode was used. Depending on the studied system, the number of experiments (n) performed with the same H_2O_2 sensor varied between 3 and 21. The shown numbers were averaged over the number of measurements performed in the respective system. To evaluate the effect of pH on the performance of the H_2O_2 sensor, a single sensor was used between pH 4.0 and 8.0 in the presence of 4 g L^{-1} xyloglucan. To cover the pH-range a 30 mM NaOAc buffer was for pH 4.0 & 5.0, whereas a 30 mM imidazole-chloride buffer was used for pH 6.0–8.0. Importantly, neither different pH values nor changing substrate concentrations affected the quick response time of <2s of the H_2O_2 sensor.

The coefficient of determination refers to the linearity of the obtained calibration function. The limit of quantification was determined by dividing 10 times the standard deviation of the baseline (nA cm^{-2}) with the sensitivity (nA μM^{-1} cm^{-2}) of the calibration function.

	**sensitivity (nA μM^{-1} cm^{-2})**	**coefficient of determination (R^2)**	**limit of quantification (LoQ) (µM)**
**buffer (n = 3)**	260 ± 30	0.9999	5.6 ± 1.2
**xyloglucan (0.125–8 g L^{-1}) (n = 21)**	220 ± 10	0.9996	4.6 ± 1.2
**PASC (0.5–6 g L^{-1}) (n=18)**	240 ± 20	0.9996	4.5 ± 1.0
**CNC (10–100 g L^{-1}) (n=18)**	220 ± 5	0.9998	4.6 ± 0.7
**H_2O_2 sensor performane between pH 4.0–8.0 (4 g L^{-1} xyloglucan, n=3 per pH)**			
**pH 4.0**	240 ± 40	0.9992	4.3±0.2
**pH 5.0**	231±6	0.9998	5.4±0.4
**pH 6.0**	244±6	0.9997	6.6±1.0
**pH 7.0**	239±8	0.9999	6.8±1.5
**pH 8.0**	220±7	0.9994	7.5±0.5

Table S3. Rates of catalysis for *Nc*AA9C acting on various (hemi-)cellulosic substrates (xyloglucan, PASC, CNC and MCC). Either ascorbate or *Nc*CDHIIA was used as reductant, as indicated in the Table. Rates were determined at standard assay conditions. All measured rateswere corrected for the substrate dependent background and the off-pathway peroxidase activity of the LPMO, which was recorded in reactions lacking substrate (indicated by 0 g L^{-1} xyloglucan). For the reaction with xyloglucan and 1 µM *Nc*CDHIIA the rate of this off-pathway activity was excessively low and no correction was needed. The initial rates for the off-pathway activity were fit with $R^2 \geq 0.92$, rates for the peroxygenase reaction were fit with an $R^2 \geq 0.98$. Some of the underlying raw-data is shown in the main text and in **Figure S6**. The fully corrected initial rates v_{enz} (µM s^{-1}) were converted to turnover numbers (TN, s^{-1}) that are independent of the LPMO concentration. The reported TNs were derived from 3 independent experiments and are provided with the standard deviation. Note that the reported TNs on xyloglucan are independent of the enzyme concentration, demonstrating a linear relationship between the enzyme dosage and H_2O_2 turnover (i.e., a linear relationship between v_{enz} and the enzyme dosage, which translates in equal TNs at all dosages).

***Nc*AA9C (nM)**	**reductant (µM)**	**xyloglucan (g L^{-1})**	**TN (s^{-1})**	**fit interval ($R^2 \geq 0.92$ / 0.98) *t* (s)**
**50**	500 (ascorbate)	4	32.7 ± 0.2	1-25
**50**	500 (ascorbate)	0	3.4 ± 0.3	1-10
**100**	500 (ascorbate)	4	30.1 ± 1.1	1-20
**100**	500 (ascorbate)	0	3.7 ± 0.2	1-10
**200**	500 (ascorbate)	4	31.9 ± 1.5	1-10
**200**	500 (ascorbate)	0	3.8 ± 0.8	1-10
**200**	20 (*Nc*CDHIIA)	4	31.9 ± 0.5	1-10
**200**	20 (*Nc*CDHIIA)	0	2.0 ± 0.5	1-10
**200**	1 (*Nc*CDHIIA)	4	5 ± 1	1-100
		**insoluble substrates (g L^{-1})**		
**200**	500 (ascorbate)	4 (PASC)	16.9 ± 1.3	1-10
**200**	500 (ascorbate)	100 (CNC)	10.5 ± 0.5	1-10
**200**	500 (ascorbate)	100 (MCC)	4.3 ± 0.6	1-10

Table S4. Half-saturating concentration for ascorbate and the extrapolated maximal turnover number ($TN_{max,app}$) of C4-oxidizing LPMOs (*Nc*AA9C, *Nc*AA9M, *Gt*AA9B, *Hj*AA9B; 50 nM) acting on 4 g L^{-1} xyloglucan and C1-oxidizing LPMOs (*Nc*AA9F, *Pc*AA9D; 200 nM) acting on 4 g L^{-1} PASC in 30 mM sodium-acetate buffer pH 6.0 at 30 °C. Underlying data points and fits are shown in **Figure S5**.

	**Half-saturating concentration for ascorbate (µM)**	**$TN_{max,app}$ (s^{-1})**
***Nc*AA9C (XG)**	70 ± 6	39 ± 1
***Nc*AA9M (XG)**	30 ± 5	41 ± 2
***Gt*AA9B (XG)**	9 ± 2	36 ± 1
***Hj*AA9B (XG)**	150 ± 30	36 ± 1
***Nc*AA9F (PASC)**	180 ± 10	2 ± 1
***Pc*AA9D (PASC)**	80 ± 20	1.7 ± 0.1

Table S5. Correlation of H_2O_2 consumed by *Nc*AA9F acting on 4 g L^{-1} PASC and concomitant formation of C1-oxidized products in the same reaction according to HPAEC-PAD analysis. The quantified C1-oxidized dimers and trimers derived from samples obtained during the TTN experiment were summed up to obtain the total amount of oxidized products (soluble and insoluble). The data displayed here are also shown in the main text. The data obtained during the H_2O_2 titration experiment was corrected with the substrate dependent consumption of H_2O_2. Therefore, oxidized products according to the H_2O_2 sensor include productive turnovers of the peroxygenase reaction and non-productive turnovers eventually leading to inactivation of the enzyme. The difference between oxidized products according to HPAEC-PAD analysis and the H_2O_2 sensor at later sampling points may be explained by an increasing off-pathway consumption of H_2O_2. Importantly, both measurements show good agreement at earlier time points (until 10 min).

	**H_2O_2 sensor**		**HPAEC-PAD analysis**
**time (min)**	total oxidized products (µM)	residual activity (%)	total oxidized products (µM)
**0**	0	100	0
**2**	95 ± 5	100 ± 6	130 ± 20
**3**	163 ± 5	90 ± 20	180 ± 20
**6**	220 ± 20	80 ± 20	213 ± 2
**8**	270 ± 10	65 ± 8	250 ± 5
**10**	296 ± 5	48 ± 7	270 ± 3
**13**	350 ± 20	32 ± 7	300 ± 20
**17**	380 ± 20	14 ± 5	310 ± 10
**21**	390 ± 30	7 ± 4	310 ± 30
**27**	400 ± 30	1 ± 5	298 ± 5

Table S6. Ranked activity of *Nc*AA9C acting on hemicelluloses and cellulosic substrates. All measurements were conducted using standard assay conditions (500 µM ascorbate, 100 µM H_2O_2, 30 mM sodium acetate buffer, pH 6.0, 100 mM KCl, at 30 °C). The substrate concentration was 4 g L^{-1} for all substrates, except for CNC and MCC (100 g L^{-1}). For hemicelluloses and cellopentaose, 50 nM *Nc*AA9C was used, while 200 nM *Nc*AA9C was used for CNC and MCC. The hemicellulosic substrates and cellopentaose are ranked according to the obtained turnover numbers, followed by amorphous and crystalline cellulose substrates. All other studied LPMOs were also tested in reactions with all the listed substrates, but were inactive on all substrates except xyloglucan, PASC and CNC. Their catalytic performance on these substrates is shown in **Figure S3**.

substrate	TN (s^{-1})	solubility
**cellopentaose**	62.5 ± 2.0	fully soluble
**β-glucan (high viscosity)**	42.2 ± 0.8	partially soluble
**β-glucan (low viscosity)**	41.7 ± 2.1	partially soluble
**glucomannan**	38.7 ± 3.0	fully soluble
**xyloglucan**	32.7 ± 0.2	fully soluble
**galactan**	15.1 ± 0.2	fully soluble
**lichenan**	10.5 ±1.4	partially soluble
**xylan (Beechwood)**	0	fully soluble
**xylan (Birchwood)**	0	fully soluble
**arabinoxylan**	0	fully soluble
**curdlan**	0	partially soluble
**mannan**	0	partially soluble
**PASC**	16.9 ± 1.3	not soluble
**CNC**	10.5 ± 0.5	not soluble
**MCC**	4.3 ± 0.6	not soluble

Table S7. Abiotic substrate-dependent change in H_2O_2 concentration. The experiment was performed using standard assay conditions at pH 6.0. In the first part of the experiment (0–5 min), the change in H_2O_2 concentration in the presence of substrate was recorded, followed by recording the change in H_2O_2 concentration in the presence of 500 µM ascorbate (5–10 min). This is termed the substrate dependent background (v_{sb}), which was used to correct the observed initial rates (v_{obs}), as described in the Experimental Procedures. The slopes were determined by linear regression. The raw data is displayed in **Figure S10**. Hemicelluloses and PASC were used at a concentration of 4 g L^{-1}, CNC and MCC at 100 g L^{-1}. The substrate dependent background (v_{sb}) in experiments with xyloglucan at non-standard pH conditions (pH 4.0, 5.0, 7.0, and 8.0), higher ascorbate (0.005 - 12 mM) and H_2O_2 concentrations (25- 300 µM) varied between 0.02 to 0.08 µM s^{-1}. It was routinely measured and used for the calculation of v_{enz}.

	**slope after addition of 100 µM H_2O_2 (µM s^{-1})**	**slope after subsequent addition of 500 µM ascorbate (µM s^{-1}) (v_{sb})**
**cellopentaose**	n.d.	n.d.
**β-glucan (high viscosity)**	0.002 ± 0.002	0.01 ± 0.01
**β-glucan (low viscosity)**	0.003 ± 0.001	0.004 ± 0.008
**glucomannan**	0.009 ± 0.002	0.09 ± 0.01
**xyloglucan**	0.009 ± 0.005	0.02 ± 0.01
**galactan**	-0.002 ± 0.001	0.03 ± 0.01
**lichenan**	-0.002 ± 0.005	0.02 ± 0.01
**xylan (Birchwood)**	0.013 ± 0.003	0.02 ± 0.01
**xylan (Beechwood)**	0.0002 ± 0.0022	0.11 ± 0.01
**arabinoxylan**	0.004 ± 0.002	0.11 ± 0.01
**mannan**	0.001 ± 0.002	0.01 ± 0.01
**curdlan**	0.001 ± 0.002	0.06 ± 0.01
**PASC**	0.009 ± 0.001	0.02 ± 0.01
**CNC**	0.017 ± 0.014	0.06 ± 0.01
**MCC**	n.d.	0.11 ± 0.08
**buffer** (30 mM sodium acetate, pH 6.0, containing 100 mM KCl)	0.003 ± 0.001	0.01 ± 0.01

Table S8. Comparison of the apparent dissociation constant of the enzyme-substrate complex ($K_{S,\mathrm{app}}$) in the presence of saturating amounts of reductant, and extrapolated maximal turnover numbers ($TN_{\mathrm{max,app}}$) for different LPMOs acting on different substrates. The $K_{\mathrm{M,app}}$ for cellopentaose reported for *Nc*AA9C is also given in millimolar concentration in parentheses. The underlying datasets are shown in **Figure 2B** of the main text and in **Figure S7A**.

LPMO	substrate	$K_{\mathrm{M,app}}$ (g L^{-1})	$TN_{\mathrm{max,app}}$ (s^{-1})
***Nc*AA9C**	xyloglucan	0.18 ± 0.03	34.8 ± 1.6
	glucomannan	3.8 ± 0.4	77 ± 3
	cellopentaose (mM)	0.9 ± 0.2 (1.2 ± 0.2)	75 ± 5
	PASC	2.3 ± 0.5	24 ± 2
	CNC	110 ± 4	21 ± 4
***Nc*AA9M**	xyloglucan	0.06 ± 0.01	36.7 ± 0.4
	PASC	3.5 ± 0.6	8 ± 1
	CNC	n.d.	n.d.

Table S9. Total turnover numbers (TTN) of LPMOs acting on various substrates. The substrates used were xyloglucan (50 µM H_2O_2 titration steps, except *Nc*AA9M which was also tested with 25 µM H_2O_2 titrations steps), PASC and CNC (10 µM H_2O_2 titration steps). The experiments were conducted under standard assay conditions using 50 nM LPMO for xyloglucan or 1 µM LPMO for PASC and CNC. To ensure an excess of reducing agent, the ascorbate concentration was 5 mM. The ascorbate stock solution was buffered to pH 6.0 to avoid pH variations in the reaction after its addition. The obtained turnover of co-substrate for individual titration steps was summed up to the total turnover numbers and corrected for the substrate dependent background consumption of H_2O_2 during the course of the experiment. The reported TTNs include productive turnovers of the peroxygenase reaction as well as off-pathway consumption of H_2O_2 eventually leading to self-inactivation indicated by a decrease in residual activity. The residual activity of the LPMOs was calculated from the slope of the last titration step (before terminating the measurement) and compared to the slope of the first titration step (=100 % activity). The initial rates used to calculate residual activities were only corrected for the substrate dependent background consumption of H_2O_2 as described in the Experimental Procedures and in the main text. The respective TTNs and residual activities were extracted from the titration data-sets of three independent experiments. After stopping the experiments, as a control, the same concentration of LPMO was added again to verify that the observed reduced LPMO rate was not caused by substrate or reductant depletion (which was indeed the case for all reported experiments). Note that the reported TTNs are minimum values, especially in cases where residual activity of the LPMOs was high when the experiment was terminated. The progress of these reactions (residual activity plotted as a function of H_2O_2 turnover) is shown in the main text, together with data obtained by off-line analysis of the amount of generated C1-oxidized products.

	**Xyloglucan**		**PASC**		**CNC**	
	**TTN (n)**	**Res. Act. (%)**	**TTN (n)**	**Res. Act. (%)**	**TTN (n)**	**Res. Act. (%)**
***Nc*AA9C**	13000 ± 2000	8 ± 2	1000 ± 50	10 ± 10	1320 ± 4	9 ± 7
***Nc*AA9M**	19300 ± 200	14 ± 1	540 ± 80	22 ± 4	1000 ± 40	80 ± 10
***Nc*AA9M**	25 µM steps: 32000± 2000	20 ± 10				
***Gt*AA9B**	15300 ± 600	33 ± 6	n.d.		800 ± 15	90 ± 20
***Nc*AA9F**	inactive		400 ± 30	1 ± 5	n.d.	
***Pc*AA9D**	inactive		325 ± 5	30 ± 10	n.d.	

Table S10. The effect of pH on the apparent saturation kinetic behavior of the enzyme-substrate, enzyme-reductant, and enzyme-H_2O_2 interaction and the extrapolated maximal turnover number ($TN_{max,app}$) of *Nc*AA9C acting on xyloglucan. Note that, while the interaction of *Nc*AA9C with xyloglucan is described with the apparent steady-state enzyme-substrate complex equilibrium constant ($K_{M,app}$), this terminology cannot be used for ascorbate and H_2O_2. Since ascorbate is not involved in catalysis (in every catalytic cycle), but only in priming the peroxygenase reaction, defining a half-saturating concentration is more suitable. In the case of H_2O_2, the data shown in **Figure S22** indicates that inactivation may have a substantial influence on the observed saturation behavior. Therefore, defining a half-saturating concentration is more appropriate than reporting a true kinetic affinity constant. Two overlapping buffers were used to cover the range between pH 4.0–8.0. Sodium acetate buffer (30 mM) was used between pH 4.0 and 6.0 while 30 mM imidazole chloride buffer was used between pH 6.0 and 8.0. Importantly, the pH-profile in the main text shows that the obtained turnover numbers at pH 6.0 (overlapping pH-profile), are independent of the used buffer system and that imidazole does not affect catalysis of *Nc*AA9C. All experiments were conducted varying only one parameter at the time and otherwise using standard assay conditions (50 nM *Nc*AA9C). The half saturating concentration for ascorbate was determined using 100 µM H_2O_2 and 4 g L^{-1} xyloglucan. Based on this data set, the ascorbate concentration was increased to 3 mM (10x half-saturating concentration at pH 5.0) to determine the $K_{M,app}$ for xyloglucan and the half-saturating concentration for H_2O_2. The underlying data to obtain half-saturating concentrations and the $K_{M,app}$ are shown in the main text.

	Half-saturating concentration for ascorbate (µM)	$TN_{max,app}$ (s^{-1})
**pH 4.0 (acetate)**	890 ± 140	37 ± 2
**pH 5.0 (acetate)**	240 ± 40	37 ± 2
**pH 6.0 (acetate)**	66 ± 6	39 ± 1
**pH 7.0 (imidazole)**	17 ± 2	55 ± 1

	$K_{M,app}$ xyloglucan (g L^{-1})	$TN_{max,app}$ (s^{-1})
**pH 5.0 (acetate)**	0.32 ± 0.03	34 ± 1
**pH 6.0 (acetate)**	0.21 ± 0.03	37 ±2
**pH 6.0 (imidazole)**	0.23 ± 0.03	37 ± 2
**pH 7.0 (imidazole)**	0.27 ± 0.05	57 ± 3

	Half-saturating concentration for H_2O_2 (µM)	$TN_{max,app}$ (s^{-1})
**pH 5.0 (acetate)**	80 ± 20	51 ± 5
**pH 6.0 (acetate)**	105 ± 20	72 ± 6
**pH 7.0 (imidazole)**	280 ± 50	190 ± 18
**pH 8.0 (imidazole)**	220 ± 30	162 ± 10

Supplementary References only found in Supporting Information

(72) Grönqvist, S.; Viikari, L.; Niku-Paavola, M. L.; Orlandi, M.; Canevali, C.; Buchert, J. Oxidation of Milled Wood Lignin with Laccase, Tyrosinase and Horseradish Peroxidase. *Appl. Microbiol. Biotechnol.* **2005**, 67, 489–494.

(73) Kuusk, S.; Kont, R.; Kuusk, P.; Heering, A.; Sørlie, M.; Bissaro, B.; Eijsink, V. G. H.; Väljamäe, P. Kinetic Insights into the Role of the Reductant in H_2O_2-Driven Degradation of Chitin by a Bacterial Lytic Polysaccharide Monooxygenase. *J. Biol. Chem.* 2019, 294(5), 1516-1528.

(74) Borisova, A. S.; Isaksen, T.; Dimarogona, M.; Kognole, A. A.; Mathiesen, G.; Várnai, A.; Røhr, Å. K.; Payne, C. M.; Sørlie, M.; Sandgren, M.; Eijsink, V. G. H. Structural and Functional Characterization of a Lytic Polysaccharide Monooxygenase with Broad Substrate Specificity. *J. Biol. Chem.* **2015**, 290, 22955–22969.

(75) Courtade, G.; Wimmer, R.; Røhr, Å. K.; Preims, M.; Felice, A. K. G.; Dimarogona, M.; Vaaje-Kolstad, G.; Sørlie, M.; Sandgren, M.; Ludwig, R.; Eijsink, V. G. H.; Aachmann, F. L. Interactions of a Fungal Lytic Polysaccharide Monooxygenase with β-Glucan Substrates and Cellobiose Dehydrogenase. *Proc. Natl. Acad. Sci.* **2016**, 113, 5922–5927.

(76) Kracher, D.; Andlar, M.; Furtmüller, P. G.; Ludwig, R. Active-Site Copper Reduction Promotes Substrate Binding of Fungal Lytic Polysaccharide Monooxygenase and Reduces Stability. *J. Biol. Chem.* **2018**, 293, 1676–1687.

(77) Vu, V. V.; Beeson, W. T.; Phillips, C. M.; Cate, J. H. D.; Marletta, M. A. Determinants of Regioselective Hydroxylation in the Fungal Polysaccharide Monooxygenases. *J. Am. Chem. Soc.* **2014**, 136, 562–565.

(78) Tan, T. C.; Kracher, D.; Gandini, R.; Sygmund, C.; Kittl, R.; Haltrich, D.; Hällberg, B. M.; Ludwig, R.; Divne, C. Structural Basis for Cellobiose Dehydrogenase Action during Oxidative Cellulose Degradation. *Nat. Commun.* **2015**, 6, 7542.

(79) Vuong, T. V.; Liu, B.; Sandgren, M.; Master, E. R. Microplate-Based Detection of Lytic Polysaccharide Monooxygenase Activity by Fluorescence-Labeling of Insoluble Oxidized Products. *Biomacromolecules* **2017**, 18, 610–616.

(80) Wu, M.; Beckham, G. T.; Larsson, A. M.; Ishida, T.; Kim, S.; Payne, C. M.; Himmel, M. E.; Crowley, M. F.; Horn, S. J.; Westereng, B.; Igarashi, K.; Samejima, M.; Ståhlberg, J.; Eijsink, V. G. H.; Sandgren, M. Crystal Structure and Computational Characterization of the Lytic Polysaccharide Monooxygenase GH61D from the Basidiomycota Fungus *Phanerochaete chrysosporium*. *J. Biol. Chem.* **2013**, 288, 12828.
